# A Cohort Study of the Patterns of Third Molar Impaction in Panoramic Radiographs in Saudi Population

**DOI:** 10.2174/1874210601711010648

**Published:** 2017-12-26

**Authors:** Mahmoud Al-Dajani, Anas O Abouonq, Turki A Almohammadi, Mohammed K Alruwaili, Rayan O Alswilem, Ibrahim A Alzoubi

**Affiliations:** 1 Henry M. Goldman School of Dental Medicine, Boston University, Boston, U.S.A.; 2 College of Dentistry, Aljouf University, Al-Jawf, Saudi Arabia

**Keywords:** Epidemiology, Impacted tooth, Oral surgery, Prevalence, Saudi Arabia, Third molar

## Abstract

**Objectives:**

To evaluate the epidemiological patterns of third molar impaction in a cohort of patients living in the north of Saudi Arabia.

**Materials and Methods:**

A retrospective cohort study comprised of analysing 2550 Orthopantomograms (OPGs) belonging to patients who attended Aljouf University College of Dentistry between September 2013 and December 2015. OPGs were examined to determine the frequency of third molar impaction, their levels of eruption and angulations. Mixed effects logistic regression analysis was performed to calculate adjusted odds ratios. Data were weighted by age and sex based on population regional estimates.

**Results:**

1551 patients (60.8%) with a mean age of 33.5 years-old (95%CI: 32.9 to 34) demonstrated 2650 impacted third molars. Third molars were more likely present in patients aged from 20 to 39 years-old (*p*<0.001); and in mandible more than maxilla (*p*<0.001). It showed highest vertical impaction and higher impaction rate in mandible than maxilla. Level A impaction was the most common among other levels by 1365 (53.5%). Vertical impaction was the most common pattern (1354 patients; 53.1%). Mesioangular impaction ranked second in mandible, while distoangular impaction ranked second in maxilla. There was no statistically significant difference between males and females concerning impaction frequency, depth levels and angulations.

**Conclusion:**

Impacted third molars is still a public health concern among youth and young adults. Vertically impacted mandibular third molars with their occlusal plane at the same level as the occlusal plane of adjacent tooth is the most prevalent pattern of third molar impaction in the northern region of Saudi Arabia.

## 1 INTRODUCTION

Removal of impacted third molars or “wisdom teeth” is a common surgical procedure performed in dental clinics. Impaction refers to the pathologic condition in which the normal eruption is hindered preventing the affected tooth from reaching a functional position in the dental arch within a predictable timeframe. The earliest recorded case of impacted third molar in human history was discovered in the mandible of 13,000- to 15,000-year-old ‘Magdalenian Girl’ that lived during the Magdalenian cultural period (18,000–10,000 BC) [1].

Several local and systematic causes can substantially lead to the impaction of third molars. Normal eruption can be distorted or even prevented by the presence of local physical barriers such as an adjacent tooth, dense overlying bone or excessive soft tissue.

Third molar is the most frequently impacted tooth. The reported prevalence of third molar impaction reveals worldwide variability ranging from 30.3% to 68.6% [2-9] This variability can be explained by the variances in race and ethnicity, and their effects on epidemiological characteristics of third molar impaction. While few studies examined some patterns of third molar impaction in the western, central and southern regions of Saudi Arabia [6, 7], none of these studies assessed the patterns of third molar impaction in the north of Saudi Arabia. Hence, the main purpose of this study was to evaluate the epidemiological patterns of third molar impaction in a cohort of patients living in the northern region of Saudi Arabia.

## 2 MATERIALS AND METHODS

### 2.1 Study Design, Participants and Setting

A retrospective cohort study was conducted. Authors examined Orthopantomograms (OPGs) belonging to patients aged 18 years old or older, who attended the clinics at the College of Dentistry, Aljouf University, between September 2013 and December 2015.

### 2.2 Case Definition

For a third molar to be considered impacted, it must satisfy two conditions: (1) the roots of third molar are completely formed except for horizontally or transversely impacted molars; and (2) there is no functional occlusion on the third molar occlusal surface. Horizontally or transversely impacted molars, which were unlikely to erupt, were included even if their roots were not completely formed.

### 2.3 Inclusion and Exclusion Criteria

The inclusion criteria were: (1) patient of 18 years old or older; and (2) presence of an OPG in patient record. On the other hand, the exclusion criteria were: (1) poor quality OPG; (2) incomplete patient record; (3) presence of any craniofacial anomalies, congenital deformities or syndromes; (4) previous history of orthodontic treatment; and (5) presence of any cyst, tumor, or other pathological condition in the molar area.

### 2.4 Impaction Depth and Angulation Classifications

Two main classifications will be applied in classifying impacted third molars in this study: (1) Pell and Gregory Classification; and (2) Winter's classification. According to Pell and Gregory Classification, impacted third molars are classified into three levels based on the depth of impaction relative to the adjacent tooth. These levels are: (1) Level A, where the occlusal plane of impacted third molar is at the same level as the occlusal plane of adjacent tooth; (2) Level B, where the occlusal plane of impacted third molar is between the occlusal plane and the cervical line of adjacent tooth; and (3) Level C, where the occlusal plane of impacted third molar is apical to the cervical line of adjacent tooth. On the other hand, Winter's classification describes the inclination of impacted third molar in relevant to the long axis of second molar. Based on Winter's classification, the angulation of impacted third molar can be defined as: Mesioangular (Ma), Distoangular (Da), Vertical (V), Horizontal (H) or Transverse (T).

### 2.5 Examiners and Standardisation Session

The examiners were four fifth-year dental students. To achieve calibration and build examiner consensus, examiners were trained and calibrated in a standardisation session held one week before the start of study. Under the direct supervision of first author, each examiner evaluated independently 100 OPGs for impacted third molars, and rated their levels of eruption as well as their angulations. A random sample of twenty OPGs were re-examined unknowingly by the examiners to assess intra-examiner reliability. Whenever a diagnostic disagreement was encountered, examiners discussed these cases and established consensus about the diagnosis. In determining the level of agreement for intra- and inter-examiner reliability, kappa analysis was performed because of the nominal categorical value of variables. In this study, a score of 0.70 or higher was considered clinically acceptable.

### 2.6 OPG Assessment and Data Collection

The four examiners were placed in separate rooms and blinded to each other’s results. The OPGs were distributed between the four examiners in random order. All OPGs were reviewed in a dark room with an x-ray viewer.

OPGs were evaluated to determine: (1) the frequency of third molar impaction; (2) levels of eruption according to Pell and Gregory Classification for the depth of impaction; and (3) angulations according to Winter's classification for the inclination of third molar in relevant to the long axis of second molar.

### 2.7 Data Analyses

All data were written using standardised form during the evaluation session. Then, all collected data were entered twice into Epi Data by two assistants in order to eliminate entry errors. All analyses were performed using SPSS package, version 23.0 (*SPSS*, Inc, Chicago, IL).

Data were summarized using univariate descriptive statistics such as frequency, mean, proportion, standard deviation and 95% confidence intervals (95%CIs) as appropriate. Bivariate inferential statistics were used to detect the relationship between third molar level of eruption or angulation on one side and independent variables on the other side. Due to the possible lack of statistical independence, mixed effects logistic regression analysis was performed to understand the relationship between dependent and independent variables (Appendix). This mixed model was used since there was some sort of clustering in the data. Unadjusted Odds Ratios (ORs) and adjusted ORs as well as their statistical significance were calculated.

To achieve population representative outcomes, data were weighted by age and sex based on population regional estimates obtained from the Saudi Central Department for Statistics and Information. The level of significance was set at (0.05).

### 2.8 Ethical Approval

This study was a retrospective evaluation of radiographs. As per privacy and confidentiality, data were anonymously collected without any identifiers. Ethical approval was obtained from the Research Ethics Board at Aljouf University.

## 3 RESULTS

### 3.1 Intra- and Inter-Examiner Reliability

The overall intra- and inter-examiner reliability showed outstanding reliability (κ>0.80). All κ values had a *p* value < 0.001. Levels of intra-examiner agreement were 0.85. The Kappa scores of inter-examiner agreement among the four examiners ranged from 0.78 to 0.85 for third molar level of eruption, and from 0.77 to 0.84 for angulation.

### 3.2 Sample Characteristics

Out of 2746 OPGs, 2550 met all the inclusion and exclusion criteria, while 196 patients were excluded at the start of this study. Between September 2013 and December 2015, the study sample included 2550 patients with mean age of 35.8 years-old (95% CI: 35.3 to 36.3). 1651 males (64.8%; mean age 35.9, 95% CI: 34.9 to 36.7) and 899 females (35.2%; mean age 35.8, 95% CI: 35.2 to 36.5) were included. Table **1** illustrates the demographic characteristics of the studied sample.

### 3.3 Third Molar Impaction Experience

**Fig. 1:**
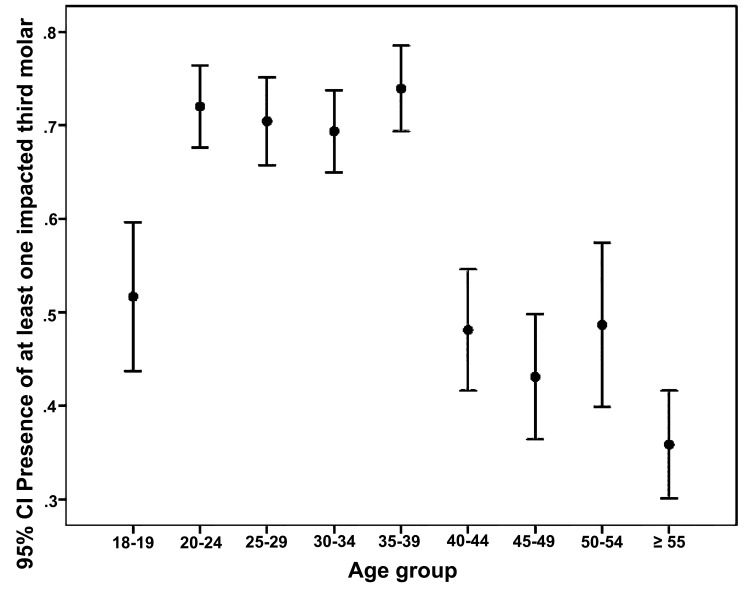
Error bars for the relationship between the presence of impacted third molar and patient’s age (N=2550).

**Fig. (2):**
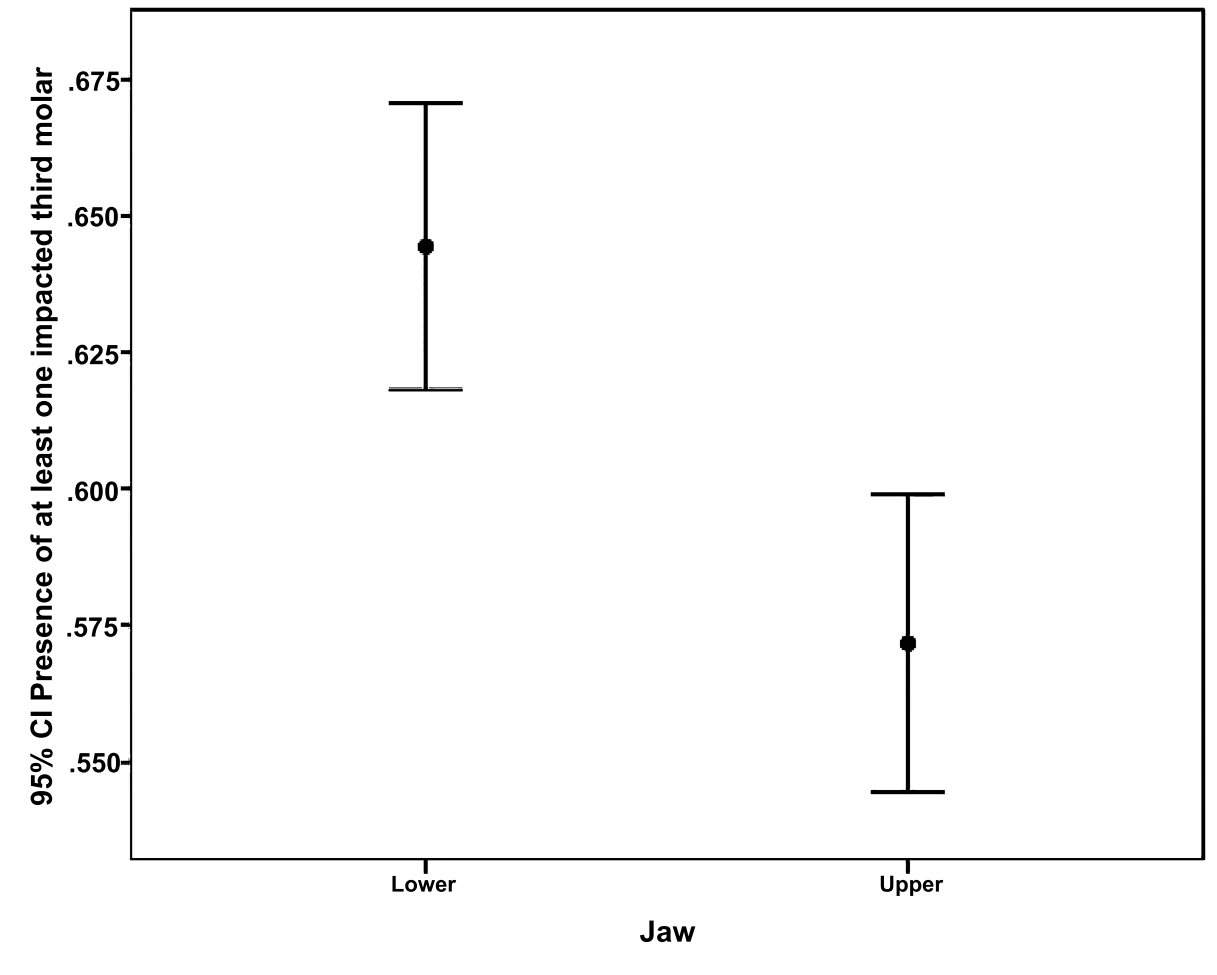
Error bars for the relationship between the presence of impacted third molar and affected jaw (N=2550).

1551 patients (60.8%) with a mean age of 33.5 years-old (95% CI: 32.9 to 34) demonstrated 2650 impacted third molars. Third molars were more likely present in patients aged from 20 to 39 years-old (*p*<0.001, chi-square test; (Fig. **1**)); and in mandible more than maxilla (*p*<0.001, chi-square test; (Fig. **2**)). The difference in third molar impaction frequency between males and females were not statistically significant (*p*=0.508, chi-square test).

Table **2** illustrates the association between sex, age or affected jaw and the prevalence of impacted third molars. As per the mixed effects binary logistic regression analysis, individuals aged from 20 to 39 had a significantly two times higher prevalence of impacted third molars than the others did, after adjusting for all covariates. Adjusted ORs for the four age groups 20-24, 25-29, 30-34 and 35-39 were 2.79, 2.32, 2.21 and 2.95, respectively (*p*≤0.001). Impacted third molars revealed a significantly higher prevalence in the mandible rather than the maxilla (adjusted OR=1.38, 95% CI: 1.12 to 1.70, *p*=0.002). However, the differences between males and females were not statistically significant (adjusted OR=1.074, 95% CI: 0.85 to 1.36, *p*=0.544).

### 3.4 Depth of Third Molar

Table **3** reveals the distribution of patients who has at least one impacted third molar based on impaction depth according to sex, age and affected jaw. Level A impaction was the most common among other levels by 1365 (53.5%) in both mandible and maxilla, while levels C and B were less common. Level A was more prevalent in patients aged from 20 to 39 years-old (*p*<0.001), as level B and C were more common in those aged from 20 to 29 years-old (*p*<0.001). All levels A, B and C were significantly more prevalent in mandible than maxilla (*p*<0.05).

**Fig. (3):**
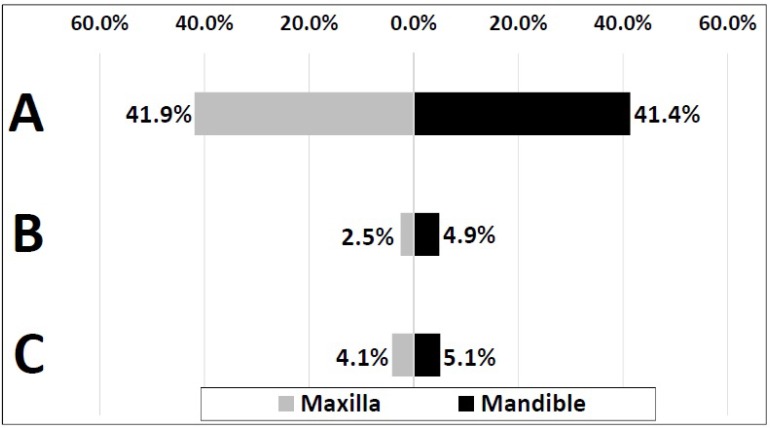
Distribution of impacted third molars based on the depth of impaction (Pell and Gregory Classification) and affected jaw (N=2650).

Out of 2650 impacted third molars, proportions of molars exhibiting level A, level B or level C were 83.3%, 7.5% and 9.2%; respectively. Level A was the most common pattern of impaction in both mandible and maxilla, while levels B and C were less observed (Fig. **3**).

Fig. (**4**) demonstrates 95% confidence interval error bars for the relationship between the depth of impaction levels A, B and C, and patient’s age. The highest prevalence of level A pattern was among patients aged from 20 to 39 years-old, while the lowest prevalence was among those aged 40 years-old and over (*p*<0.001, chi-square test). Level B pattern was most common among patients aged from 20 to 24 years-old (*p*<0.001, chi-square test). Level C has significantly highest prevalence among patients aged from 18 to 29 years-old (*p*<0.001, chi-square test).

Table **4** shows the results of mixed effects logistic regression for patients based on the impaction depth of impacted third molar. Level A impaction was significantly most prevalent among youth and young adults aged from 20 to 39 (*p*<0.005). The three levels of impaction were more encountered in the mandible (*p*<0.05). There was no statistically significant difference between males and females concerning impaction depth levels.

### 3.5 Angulation of Third Molar

Table **5** discloses the distribution of patients who has at least one impacted third molar based on third molar inclination (Winter's classification) according to sex, age and affected jaw. Overall, V impaction was the most common pattern (1354 patients; 53.1%), while T impaction was the least common (6 patients; 0.2%).

**Fig. (5):**
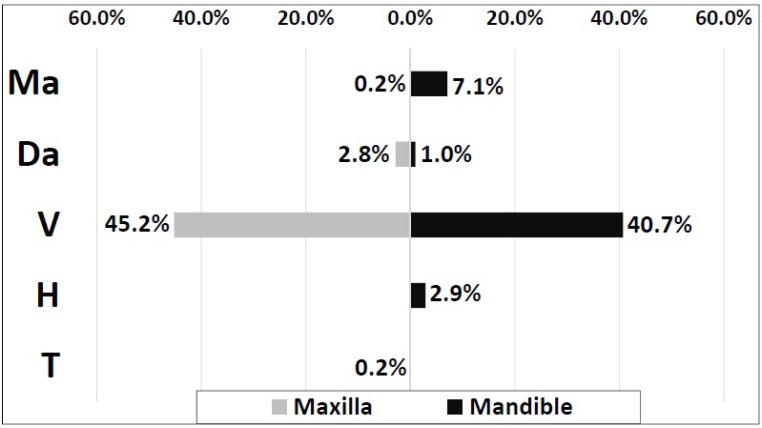
Distribution of impacted third molars based on their angulations (Winter's classification) and affected jaw (N=2650).

Out of 2650 impacted third molars, proportions of molars exhibiting the Ma, Da, V, H or T impaction were 7.3%, 3.7%, 85.9%, 2.9% and 0.2%; respectively. V impaction was the most common pattern of impaction in both mandible (40.7%) and maxilla (45.2%), while T angulation was the least observed in both jaws. In the impacted lower third molars, Ma and Da angulations came second and third (7.1% and 1%; respectively). In upper third molars, Da angulation came second (2.8%) (Fig. **5**).

Fig. (**6**) demonstrates 95% confidence interval error bars for the relationship between the third molar angulation, and patient’s age. The highest prevalence of Ma impaction was among youth (*p*<0.05, chi-square test). V impaction was most common among patients aged from 20 to 39 years-old (*p*<0.05, chi-square test).

Table **6** shows the results of mixed effects logistic regression for patients based on the inclination of impacted third molar. T impaction did not appear in the table since all its adjusted ORs were not statistically significant. V impaction was significantly most prevalent among youth and young adults (aged 20 to 39 years-old), while least prevalent among those aged 55 years-old and over. Ma impaction was four times more prevalent in mandible (adjusted OR=3.88, 95% CI: 2.68 to 5.61, *p*<0.001). H impaction was two times more prevalent in mandible (adjusted OR=2.38, 95% CI: 1.59 to 3.57, *p*<0.001), whereas Da impaction was more prevalent in the maxilla. There was no statistically significant difference between males and females in all inclination patterns.

## 4 DISCUSSION

The aim of this study was to evaluate the epidemiological patterns of third molar impaction in a cohort of patients living in the northern region of Saudi Arabia (N=2550). This study gave population-based estimates about the prevalence of impacted third molars in the studied population, and most importantly revealed the distribution of impacted molars by their impaction depth and angulation according to age, sex and affected jaw (maxilla or mandible).

Training and calibrating examiners in a standardization session allowed achieving an outstanding overall intra- and inter-examiner reliability (κ>0.80). As well, weighting data by sex and age, using population estimates obtained from the Saudi Central Department for Statistics and Information, permitted concluding population representative estimates. Applying stringent methodological approaches assured the accuracy and generalizability of our study results.

Approximately more than half of patients (60.8%) with a mean age of 33.5 years-old revealed possessing at least one impacted third molar. Impacted third molars were more prevalent in mandible than maxilla. Individuals aged from 20 to 39 had a significantly two times higher prevalence of impacted third molars than the others did. The higher prevalence of impacted third molars among youth and young adults is a public health concern, in which early diagnosis and prophylactic interventions can be of great benefit in preventing negative consequences on dental and general health [10]

Some studies found females to be more frequently affected by impacted third molars [9, 11], while others found more impacted third molars in males [12, 13] Among these inconsistent sexual predilections in third molar impaction, our study discovered no statistically significant difference in the prevalence of impacted third molar between males and females. To put it differently, males and females are both susceptible to having an impacted third molar, whereas sex does not appear to be a potential risk factor for impaction incidence.

The depth of impaction relevant to adjacent tooth is considered one of the key predictors in anticipating the difficulty of surgical intervention [14] For example, extracting a level A third molar can be easier than level B molar, and level B molar can be easier than level C molar. More than the half of impacted third molars (53.5%) were having their occlusal plane at the same level as the occlusal plane of adjacent tooth (level A). Level A impaction was significantly most prevalent among youth and young adults aged from 20 to 39. However, there was no statistically significant difference between males and females concerning impaction depth levels.

Angulation of third molar is another factor to consider when predicting the difficulty of surgical intervention. V impaction was the most common pattern in maxilla and mandible. Ma impaction ranked second in mandible, while Da impaction ranked second in maxilla. Previous studies showed some inconsistency. Ma impaction was the most common pattern in some of these studies [9, 13, 15], while others were in agreement with our study and stated that V impaction was the most common [16, 17]

V impaction was significantly most prevalent among youth and young adults (aged 20 to 39 years-old), while least prevalent among those aged 55 years-old and over. Ma and H impactions were more prevalent in mandible than maxilla, whereas Da impaction was more prevalent in the maxilla. There was no statistically significant difference between males and females in all inclination patterns.

Being the most common pattern of impacted molars, level A impacted mandibular third molars with V angulation are at highest risk for developing pericoronitis [14, 17]. To avoid the severe consequences of pericoronitis, prophylactic interventions targeting these third molars can be very viable in preventing any possible harm or even loss.

As a rule, all impacted teeth should be evaluated for definitive treatment, which include either observation, sustained oral hygiene improvements, operculectomy, surgical extraction or even ortho-surgically assisted eruption. Before making treatment decisions on retaining or removing the impacted teeth, dentists should depend on clinical and radiographic examinations using their knowledge, training and expertise [18].

Panoramic radiographs are the “workhorse” in impacted third molar surgery, and in the authors’ opinion, they should be obtained for all surgical cases. They conveniently unveil a large area of the dental and facial tissues in one view. OPGs assist dentist not only in reaching a diagnosis, but also in making clinical decisions concerning surgical interventions near vital structures. For example, OPGs allow better judgement of the closeness of third molar roots to the inferior alveolar nerve, the approximate proximity of maxillary tooth roots to the sinus, as well as any potential pathology that would otherwise be missed in more focused fields.

Dental impaction is a frequent phenomenon, and the number of patients referred to dentist and oral surgeons with impacted third molars is increasing every year. The OPG can give important anatomical and pathological information pertinent to dental and maxillofacial structures. The OPG is commonly considered the initial modality for the evaluation of impacted teeth.

Never the least, several factors contribute to the incidence of dental impaction such as the lack of space on the distal side of second molars. Future studies should focus on exploring factors that can prevent normal eruption. Knowing all these factors is vital in predicting dental impaction and consequently preventing its occurrence.

## CONCLUSION

Impacted third molars is still a public health concern among youth and young adults. Individuals between the age of 18 and 29 should be examined to weight the risks or benefits for extracting the impacted third molar. Vertically impacted mandibular third molars with their occlusal plane at the same level as the occlusal plane of adjacent tooth is the most prevalent pattern of third molar impaction in the northern region of Saudi Arabia.

## Figures and Tables

**Fig. (4):**
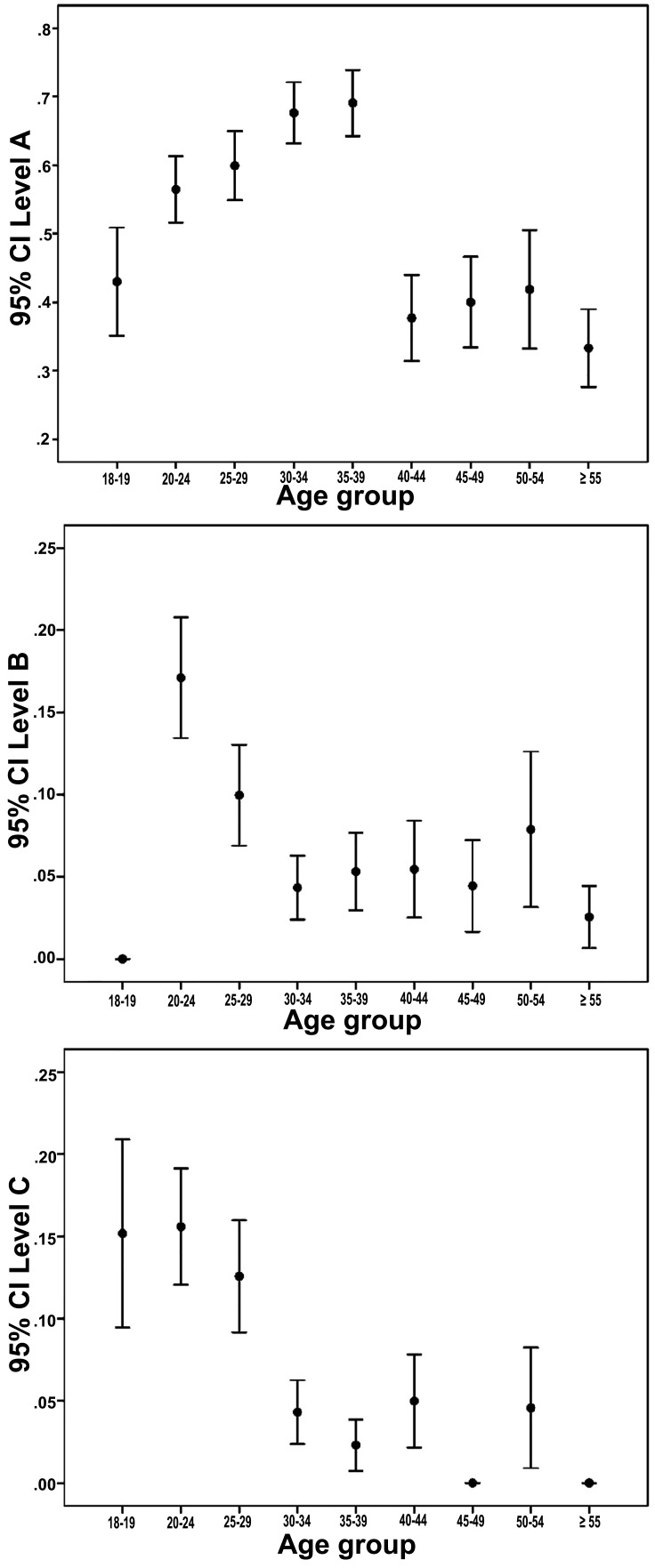
Error bars for the relationship between the depth of impaction levels A, B and C, and patient’s age (N=2550).

**Fig. (6):**
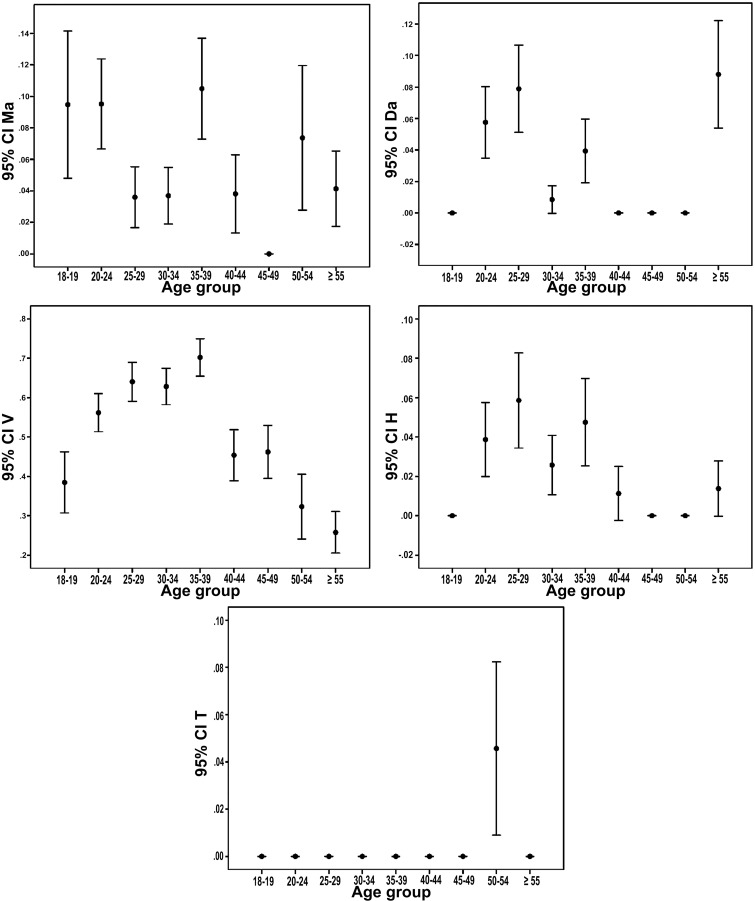
Error bars for the relationship between the third molar angulation and patient’s age (N=2550).

**Table 1:** Demographic characteristics.

-	-	**No.**	**%**	**Mean Age** **(Years)**	**95% Confidence** **Interval**
**Total**		2550	100.0	35.S8	(35.3, 36.3)
**Sex**	Male	1651	64.8	35.9	(34.9, 36.7)
	Female	899	35.2	35.8	(35.2, 36.5)
**Age**	18-19	154	6.1	18.6	(18.5, 18.6)
	20-24	407	16.0	22.6	(22.5, 22.7)
	25-29	366	14.4	26.6	(26.4, 26.7)
	30-34	425	16.7	31.6	(31.5, 31.8)
	35-39	356	14.0	36.2	(36.1, 36.4)
	40-44	231	9.1	41.4	(41.2, 41.6)
	45-49	213	8.4	45.6	(45.4, 45.7)
	50-54	128	5.0	53.0	(52.8, 53.2)
	≥55	269	10.5	63.8	(63.2, 64.4)

**Table 2:** Bivariate and mixed effects binary logistic regression analyses for patients with and without impacted third molars (N=2550).

-	-	**With Impacted Third Molar**		**No Impacted Third Molar**		-	-	-	-	-	-
		**N**	**%**	**N**	**%**	**Unadjusted OR**	**95% CI**	***p***	**Adjusted OR**	**95% CI**	***p***
**Sex**	Male	1012	65.2	639	64.0	0.95	(0.8, 1.12)	0.508	1.074	(0.85, 1.36)	0.544
	Female	539	34.8	360	36.0	Reference			Reference		
**Age**	18-19	80	5.2	75	7.5	Reference			Reference		
	20-24	293	18.9	114	11.4	2.41	(1.64, 3.53)	0.000	2.79	(1.63, 4.77)	0.000
	25-29	258	16.6	108	10.8	2.23	(1.51, 3.29)	0.000	2.32	(1.41, 3.79)	0.001
	30-34	295	19.0	130	13.0	2.12	(1.45, 3.09)	0.000	2.21	(1.38, 3.55)	0.001
	35-39	263	17.0	93	9.3	2.65	(1.79, 3.94)	0.000	2.95	(1.78, 4.87)	0.000
	40-44	111	7.2	120	12.0	0.87	(0.58, 1.3)	0.493	0.87	(0.51, 1.49)	0.608
	45-49	92	5.9	121	12.1	0.71	(0.47, 1.07)	0.104	0.66	(0.37, 1.18)	0.158
	50-54	62	4.0	66	6.6	0.89	(0.55, 1.42)	0.614	0.86	(0.49, 1.52)	0.607
	≥55	96	6.2	172	17.2	0.52	(0.35, 0.78)	0.002	0.49	(0.29, 0.82)	0.007
**Jaw**	Lower	825	53.2	455	45.5	1.36	(1.16, 1.59)	0.000	1.38	(1.12, 1.70)	0.002
	Upper	726	46.8	544	54.5	reference			reference		

OR, odds ratio; CI, confidence interval

Mixed effects regression was used to model the binary outcome variable

**Table 3:** Distribution of patients based on the depth of impaction (Pell and Gregory Classification) according to sex, age and affected jaw (N=2550).

-	-	**Level A**			**Level B**			**Level C**		
		**N**	**%**	***p***	**N**	**%**	***p***	**N**	**%**	***p***
**Total (N=2550)**		1365	53.5%		182	7.1%		177	6.9%	
**Sex**	Male	889	65.1%		122	67.0%		106	59.9%	0.161
	Female	476	34.9%	0.675	60	33.0%	0.507	71	40.1%	
**Age group**	18-19	66	4.8%	<0.001	0	0.0%	<0.001	23	13.0%	<0.001
	20-24	230	16.9%		70	38.5%		64	36.2%	
	25-29	220	16.1%		36	19.8%		46	26.0%	
	30-34	288	21.1%		18	9.9%		18	10.2%	
	35-39	246	18.0%		19	10.4%		8	4.5%	
	40-44	87	6.4%		13	7.1%		12	6.8%	
	45-49	85	6.2%		9	4.9%		0	0.0%	
	50-54	53	3.9%		10	5.5%		6	3.4%	
	≥55	89	6.5%		7	3.8%		0	0.0%	
**Jaw**	Lower	714	52.3%	0.019	115	63.2%	<0.001	108	61.0%	0.003
	Upper	650	47.7%		67	36.8%		69	39.0%	

**Table 4:** Mixed effects logistic regression results for patients based on the third molar depth of impaction (N=2550).

-	-	**A**	-	-	**B**	-	-	**C**	-	-
		**OR**	**95% CI**	***p***	**OR**	**95% CI**	***p***	**OR**	**95% CI**	***p***
**Sex**	Male	1.04	(0.83, 1.3)	0.761	1.24	(0.85, 1.82)	0.268	0.96	(0.66, 1.38)	0.817
	Female	Reference			Reference			Reference		
**Age Group**	18-19	Reference			Reference			Reference		
	20-24	1.86	(1.10, 3.14)	0.020	8.66	(2.81, 26.65)	0.000	1.29	(0.69, 2.40)	0.425
	25-29	2.08	(1.28, 3.38)	0.003	3.80	(1.26, 11.47)	0.018	0.80	(0.45, 1.43)	0.452
	30-34	2.95	(1.84, 4.74)	0.000	1.96	(0.64, 6.01)	0.239	0.32	(0.17, 0.61)	0.000
	35-39	3.28	(2.00, 5.39)	0.000	2.30	(0.75, 7.07)	0.146	0.25	(0.13, 0.50)	0.000
	40-44	0.81	(0.47, 1.39)	0.448	2.28	(0.69, 7.61)	0.179	0.35	(0.16, 0.77)	0.009
	45-49	0.83	(0.46, 1.49)	0.532	2.01	(0.60, 6.70)	0.257	0.16	(0.06, 0.42)	0.000
	50-54	0.88	(0.50, 1.55)	0.646	2.82	(0.83, 9.50)	0.095	0.41	(0.18, 0.92)	0.032
	≥55	0.62	(0.37, 1.05)	0.076	1.57	(0.48, 5.12)	0.457	0.16	(0.07, 0.38)	0.000
**Jaw**	Lower	1.27	(1.04, 1.56)	0.020	1.54	(1.13, 2.09)	0.006	1.36	(1.01, 1.85)	0.045
	Upper	Reference			Reference			Reference		
OR, adjusted odds ratio; CI, confidence interval.										

Mixed effects regression was run separately for each dependent variable.

**Table 5:** Distribution of patients based on the inclination of third molar to the long axis of second molar (Winter's classification) according to sex, age and affected jaw (N=2550).

-	-	**Ma**			**Da**			**V**			**H**			**T**		
		**N**	**%**	***p***	**N**	**%**	***p***	**N**	**%**	***p***	**N**	**%**	***p***	**N**	**%**	***p***
**Total (N=2550)**		149	5.8%		94	3.7%		1354	53.1%		72	2.8%		6	0.2%	
**Sex**	Male	106	71.1%		60	63.8%		906	66.9%		45	62.5%		0	0.0%	0.001
	Female	43	28.9%	0.088	34	36.2%	0.850	448	33.1%	0.015	27	37.5%	0.684	6	100.0%	
**Age group**	18-19	15	10.1%	<0.001	0	0.0%	<0.001	59	4.4%	<0.001	0	0.0%	<0.001	0	0.0%	<0.001
	20-24	39	26.2%		23	24.5%		229	16.9%		16	22.2%		0	0.0%	
	25-29	13	8.7%		29	30.9%		235	17.4%		21	29.2%		0	0.0%	
	30-34	16	10.7%		4	4.3%		267	19.7%		11	15.3%		0	0.0%	
	35-39	37	24.8%		14	14.9%		250	18.5%		17	23.6%		0	0.0%	
	40-44	9	6.0%		0	0.0%		105	7.8%		3	4.2%		0	0.0%	
	45-49	0	0.0%		0	0.0%		99	7.3%		0	0.0%		0	0.0%	
	50-54	9	6.0%		0	0.0%		41	3.0%		0	0.0%		6	100.0%	
	≥55	11	7.4%		24	25.5%		69	5.1%		4	5.6%		0	0.0%	
**Jaw**	Lower	144	96.6%	<0.001	25	26.6%	<0.001	665	49.2%	0.270	71	100.0%	<0.001	0	0.0%	0.014
	Upper	5	3.4%		69	73.4%		688	50.8%		0	0.0%		6	100.0%	

**Table 6:** Mixed effects logistic regression results for patients based on the third molar inclination (N=2550).

-	-	**Ma**	-	-	**Da**	-	-	**V**	-	-	**H**	-	-
		**OR**	**95% CI**	***p***	**OR**	**95% CI**	***p***	**OR**	**95% CI**	***p***	**OR**	**95% CI**	***p***
**Sex**	Male	1.34	(0.91, 1.97)	0.134	1.05	(0.70, 1.59)	0.807	1.31	(1.05, 1.65)	0.019	0.99	(0.64, 1.52)	0.952
	Female	Reference			Reference			Reference			Reference		
**Age Group**	18-19	Reference			Reference			Reference			Reference		
	20-24	1.21	(0.56, 2.62)	0.629	2.77	(0.86, 8.98)	0.089	2.71	(1.60, 4.59)	0.000	2.08	(0.64, 6.78)	0.223
	25-29	0.51	(0.24, 1.10)	0.086	3.07	(1.01, 9.37)	0.049	2.85	(1.75, 4.66)	0.000	2.41	(0.79, 7.34)	0.122
	30-34	0.52	(0.25, 1.08)	0.079	1.16	(0.36, 3.72)	0.801	2.69	(1.68, 4.31)	0.000	1.53	(0.50, 4.71)	0.455
	35-39	1.26	(0.64, 2.48)	0.505	1.92	(0.62, 6.00)	0.260	4.00	(2.42, 6.60)	0.000	2.13	(0.70, 6.48)	0.184
	40-44	0.53	(0.22, 1.28)	0.160	1.00	(0.26, 3.89)	0.997	1.29	(0.75, 2.21)	0.359	1.21	(0.33, 4.41)	0.773
	45-49	0.30	(0.11, 0.80)	0.016	1.00	(0.27, 3.79)	0.998	1.28	(0.71, 2.29)	0.407	1.00	(0.27, 3.70)	1.000
	50-54	0.76	(0.32, 1.81)	0.535	1.01	(0.24, 4.21)	0.993	0.66	(0.37, 1.19)	0.164	1.00	(0.24, 4.08)	0.999
	≥55	0.56	(0.26, 1.22)	0.145	3.39	(1.11, 10.39)	0.033	0.49	(0.29, 0.83)	0.008	1.26	(0.38, 4.16)	0.704
**Jaw**	Lower	3.88	(2.68, 5.61)	0.000	0.66	(0.46, 0.94)	0.023	0.90	(0.74, 1.10)	0.306	2.38	(1.59, 3.57)	0.000
	Upper	Reference			Reference			Reference			Reference		

OR, adjusted odds ratio; CI, confidence interval.

Mixed effects regression was run separately for each dependent variable.
