# Supplementary material for: A Cohort Study of the Patterns of Third Molar Impaction in Panoramic Radiographs in Saudi Population
Source: Open Dent J. 2017 Dec 26;11:648–60. doi: 10.2174/1874210601711010648 (PMC5750684; doi:10.2174/1874210601711010648)
Supplement: Supplementary file 1 [file TODENTJ-11-648_SD1.pdf]

Model Summary

Target: Level A

**Target**		Level A
**Probability Distribution**		Binomial
**Link Function**		Logit
**Information Criterion**	**Akaike Corrected**	10,544.242
	**Bayesian**	10,550.024

Information criteria are based on the -2 log pseudo likelihood (10,542.241) and are used to compare models. Models with smaller information criterion values fit better. When comparing models using pseudo likelihood values, caution should be used because different data transformations may be used across the models.

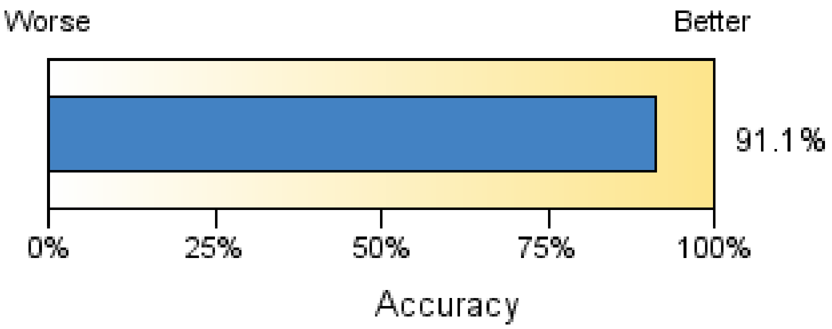

Data Structure

Target: Level A

	Subjects	Target
	Patient ID	Level A
Data For First Subject	1	0
Total Number of Levels	1979	

Classification

Target:Level A

Overall Percent Correct =91.1%

Observed	Predicted	
	1	0
1	97.3%	2.7%
0	16.2%	83.8%

Row Percent

- 100.00
- 80.00
- 60.00
- 40.00
- 20.00
- 0.00

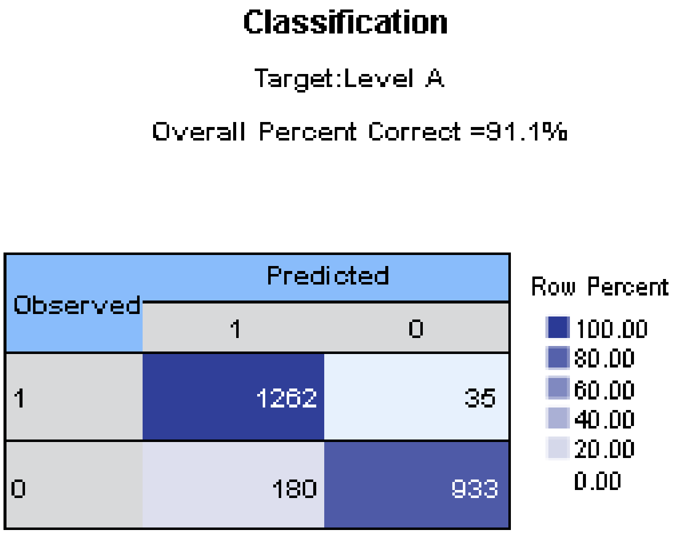
Classification
Target:Level A
Overall Percent Correct =91.1%
Predicted
Observed
1
0
1
1262
35
0
180
933
Row Percent
100.00
80.00
60.00
40.00
20.00
0.00

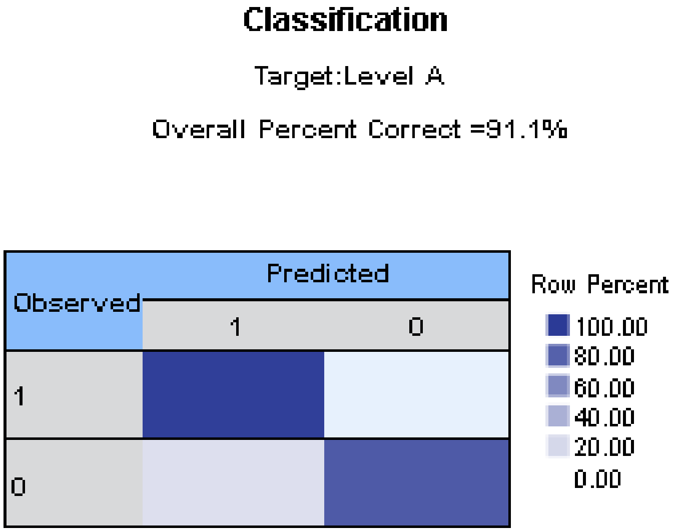
Classification
Target:Level A
Overall Percent Correct =91.1%
Predicted
Observed
1
0
1
0
Row Percent
100.00
80.00
60.00
40.00
20.00
0.00

Classification

Target:Level A

Overall Percent Correct =91.1%

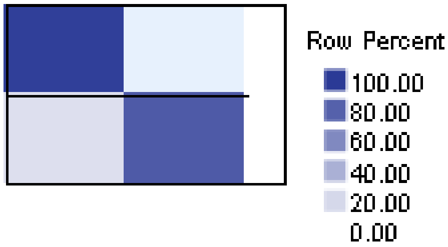

Fixed Effects

Target:Level A
Reference Category:0

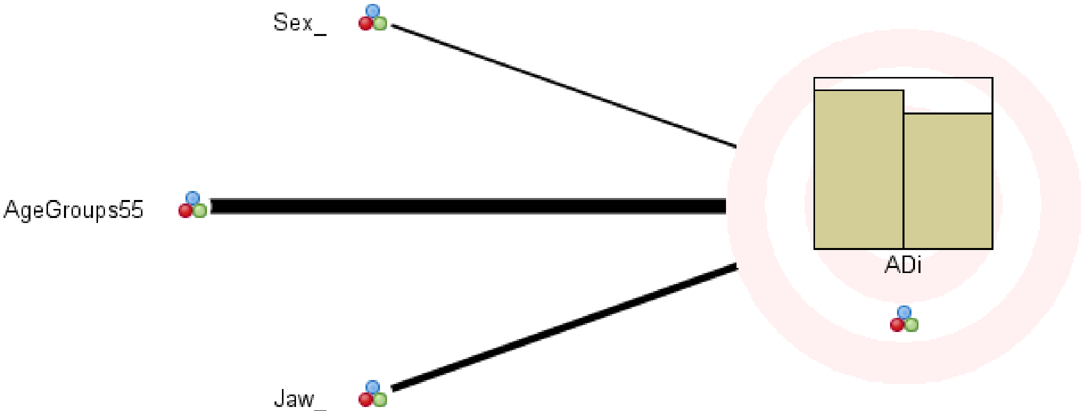

Fixed Effects

Target:Level A
Reference Category:0

Source	F	df1	df2	Sig.
Corrected Model	13.786	10	2,399	.000

Probability distribution:Binomial
Link function:Logit

Fixed Effects

Target:Level A
Reference Category:0

Source	F	df1	df2	Sig.
Corrected Model ▼	13.786	10	2,399	.000
Sex_	0.093	1	2,399	.761
AgeGroups55	16.648	8	2,399	.000
Jaw_	5.456	1	2,399	.020

Probability distribution:Binomial
Link function:Logit

Fixed Coefficients

Target:Level A
Reference Category:0

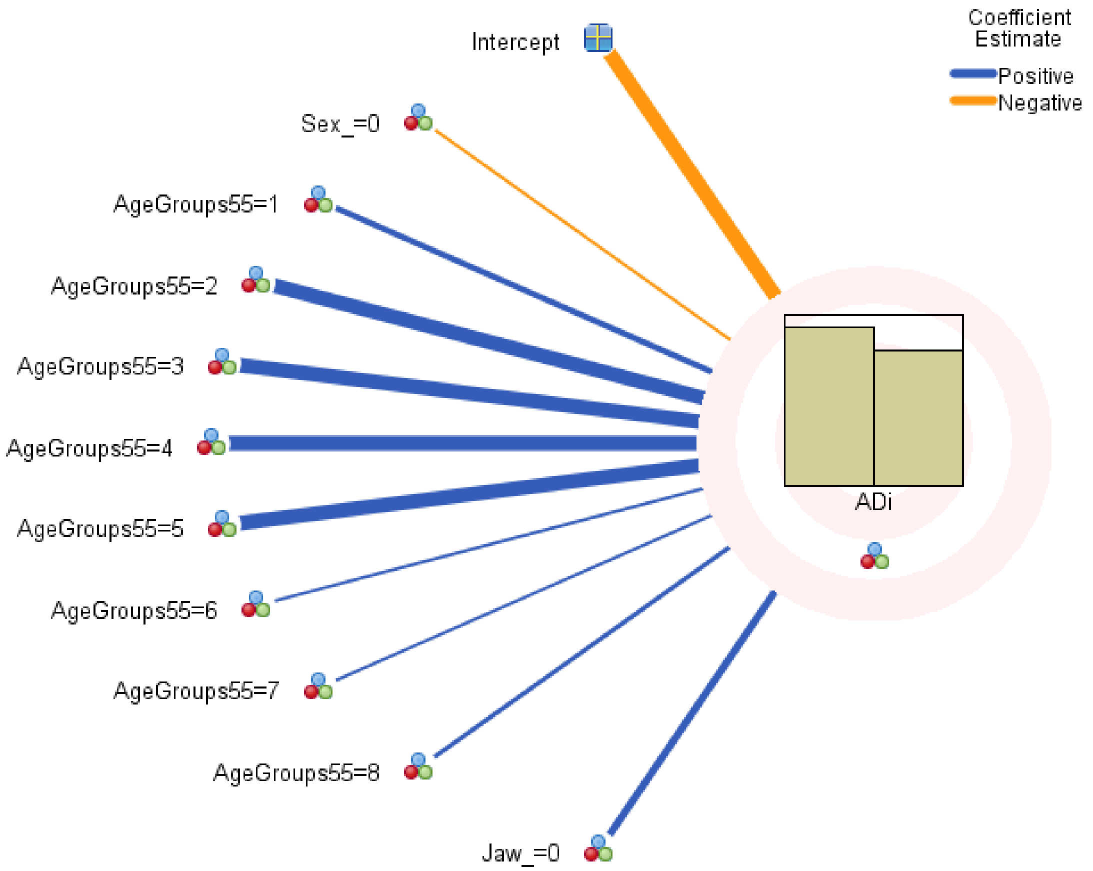

Fixed Coefficients

Target:Level A
Reference Category:0

Model Term	Coefficient	Sig.	Exp(Coefficient)
Intercept	-0.889	.000	0.411
Sex_=0	-0.035	.761	0.965
Sex_=1	0[a]		
AgeGroups55=1	0.473	.076	1.605
AgeGroups55=2	1.093	.000	2.982
AgeGroups55=3	1.203	.000	3.331
AgeGroups55=4	1.556	.000	4.740
AgeGroups55=5	1.661	.000	5.264
AgeGroups55=6	0.263	.272	1.301
AgeGroups55=7	0.286	.281	1.331
AgeGroups55=8	0.339	.185	1.404
AgeGroups55=9	0[a]		
Jaw_=0	0.240	.020	1.272
Jaw_=1	0[a]		

Probability distribution:Binomial
Link function:Logit

[a] This coefficient is set to zero because it is redundant.

Fixed Coefficients

Target:Level A
Reference Category:0

Model Term	Coefficient	Sig.
Intercept	-0.889	.000
Sex_=0	-0.035	.761
Sex_=1	0[a]	
AgeGroups55=1	0.473	.076
AgeGroups55=2	1.093	.000
AgeGroups55=3	1.203	.000
AgeGroups55=4	1.556	.000
AgeGroups55=5	1.661	.000
AgeGroups55=6	0.263	.272
AgeGroups55=7	0.286	.281
AgeGroups55=8	0.339	.185
AgeGroups55=9	0[a]	
Jaw_=0	0.240	.020
Jaw_=1	0[a]	

Probability distribution:Binomial
Link function:Logit

[a]This coefficient is set to zero because it
redundant.

Fixed Coefficients

Target:Level A
Reference Category:0

Model Term	Coefficient	Std.Error	t	Sig.	Exp(Coefficient)
Intercept	-0.889	0.175	-5.081	.000	0.411
Sex_=0	-0.035	0.116	-0.304	.761	0.965
Sex_=1	0[a]				
AgeGroups55=1	0.473	0.267	1.775	.076	1.605
AgeGroups55=2	1.093	0.228	4.798	.000	2.982
AgeGroups55=3	1.203	0.206	5.837	.000	3.331
AgeGroups55=4	1.556	0.198	7.868	.000	4.740
AgeGroups55=5	1.661	0.213	7.801	.000	5.264
AgeGroups55=6	0.263	0.240	1.098	.272	1.301
AgeGroups55=7	0.286	0.265	1.078	.281	1.331
AgeGroups55=8	0.339	0.256	1.325	.185	1.404
AgeGroups55=9	0[a]				
Jaw_=0	0.240	0.103	2.336	.020	1.272
Jaw_=1	0[a]				

Probability distribution:Binomial
Link function:Logit

[a] This coefficient is set to zero because it is redundant.

Fixed Coefficients

Target:Level A
Reference Category:0

Model Term	95% Confidence Interval for Exp (Coefficient)	
	Lower	Upper
Intercept	0.292	0.579
Sex_=0	0.768	1.213
Sex_=1		
AgeGroups55=1	0.952	2.706
AgeGroups55=2	1.908	4.660
AgeGroups55=3	2.223	4.991
AgeGroups55=4	3.216	6.986
AgeGroups55=5	3.467	7.991
AgeGroups55=6	0.813	2.082
AgeGroups55=7	0.791	2.240
AgeGroups55=8	0.850	2.320
AgeGroups55=9		
Jaw_=0	1.039	1.556
Jaw_=1		

Probability distribution:Binomial
Link function:Logit

[a] This coefficient is set to zero because it is redundant.

Fixed Coefficients

Target:Level A
Reference Category:0

Model Term	Coefficient	Std.Error	t	Sig.	95% Confidence Interval	
					Lower	Upper
Intercept	-0.889	0.175	-5.081	.000	-1.232	-0.546
Sex_=0	-0.035	0.116	-0.304	.761	-0.264	0.193
Sex_=1	0[a]					
AgeGroups55=1	0.473	0.267	1.775	.076	-0.050	0.996
AgeGroups55=2	1.093	0.228	4.798	.000	0.646	1.539
AgeGroups55=3	1.203	0.206	5.837	.000	0.799	1.608
AgeGroups55=4	1.556	0.198	7.868	.000	1.168	1.944
AgeGroups55=5	1.661	0.213	7.801	.000	1.243	2.078
AgeGroups55=6	0.263	0.240	1.098	.272	-0.207	0.733
AgeGroups55=7	0.286	0.265	1.078	.281	-0.234	0.807
AgeGroups55=8	0.339	0.256	1.325	.185	-0.163	0.842
AgeGroups55=9	0[a]					
Jaw_=0	0.240	0.103	2.336	.020	0.039	0.442
Jaw_=1	0[a]					

Probability distribution:Binomial
Link function:Logit

[a]This coefficient is set to zero because it is redundant.

Random Effect Covariances

Target: Level A

Covariance

Negative Positive

	Intercept
**Intercept**	1.231

Covariance Structure:Variance components
Subject Specification:ID

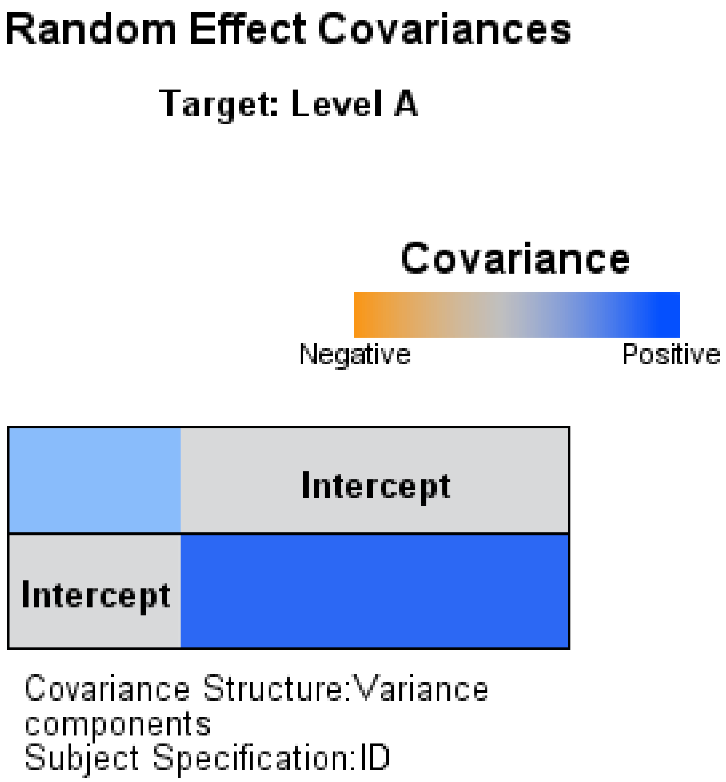
Random Effect Covariances
Target: Level A
Covariance
Negative
Positive
Intercept
Intercept
Covariance Structure:Variance components
Subject Specification:ID

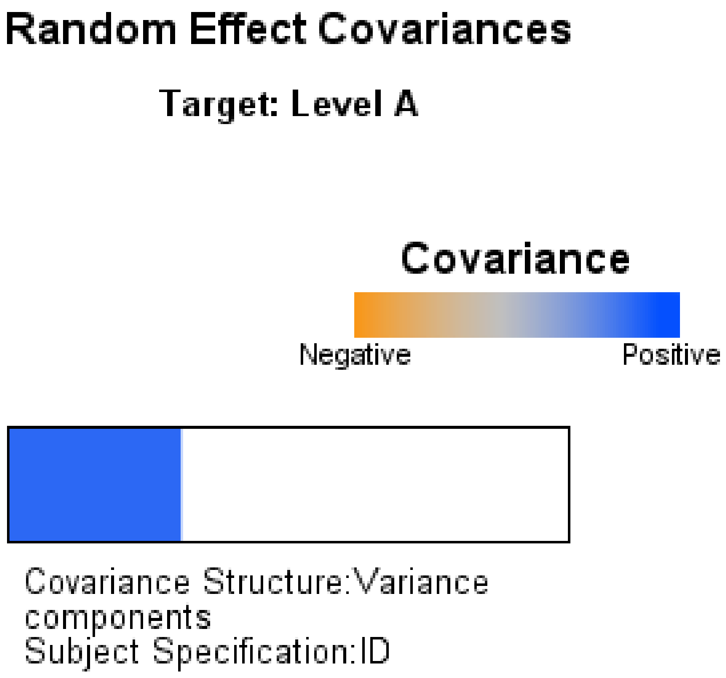
Random Effect Covariances
Target: Level A
Covariance
Negative
Positive
Covariance Structure:Variance components
Subject Specification:ID

Covariance Parameters

Target:Level A

**Covariance Parameters**	**Residual Effect**	0
	**Random Effects**	1
**Design Matrix Columns**	**Fixed Effects**	14
	**Random Effects**	1[a]
**Common Subjects**		1,979

Common subjects are based on the subject specifications for the residual and random effects and are used to chunk the data for better performance.

[a]This is the number of columns per common subject.

**Residual Effect**	**Estimate**	**Std.Error**	**Z**	**Sig.**	**95% Confidence Interval**	
					**Lower**	**Upper**
**Variance**	1.000	.	.	.	.	.

Covariance Structure:Scaled Identity
Subject Specification:(None)

Covariance Parameters

Target:Level A

**Covariance Parameters**	**Residual Effect**	0
	**Random Effects**	1
**Design Matrix Columns**	**Fixed Effects**	14
	**Random Effects**	1[a]
**Common Subjects**		1,979

Common subjects are based on the subject specifications for the residual and random effects and are used to chunk the data for better performance.

[a] This is the number of columns per common subject.

Random Effect	Estimate	Std.Error	Z	Sig.	95% Confidence Interval	
					Lower	Upper
**Var(Intercept)**	1.231	0.146	8.455	.000	0.976	1.552

Covariance Structure:Variance components
Subject Specification:ID

Estimated Means: Significant Effects

Target: Level A

Estimated means charts for significant effects (p<.05) are displayed. Up to ten effects are displayed, beginning with the top three-way effects. Effects shown contain categorical predictors only.

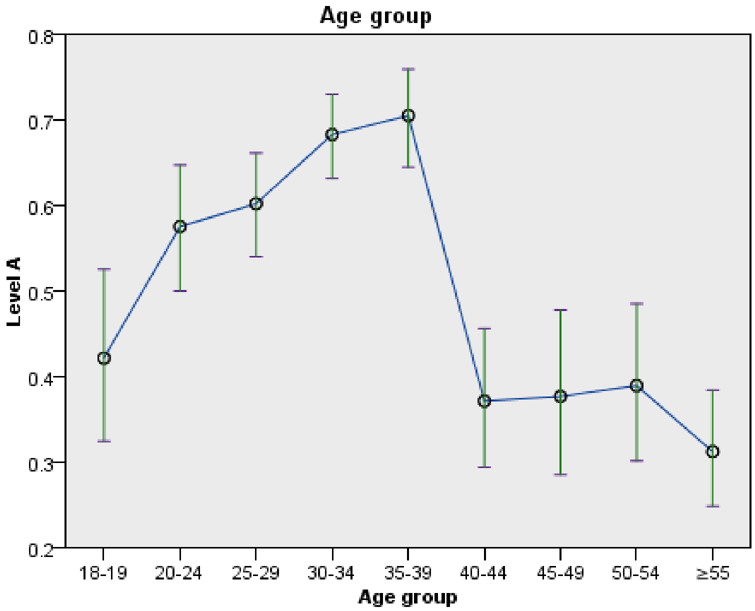

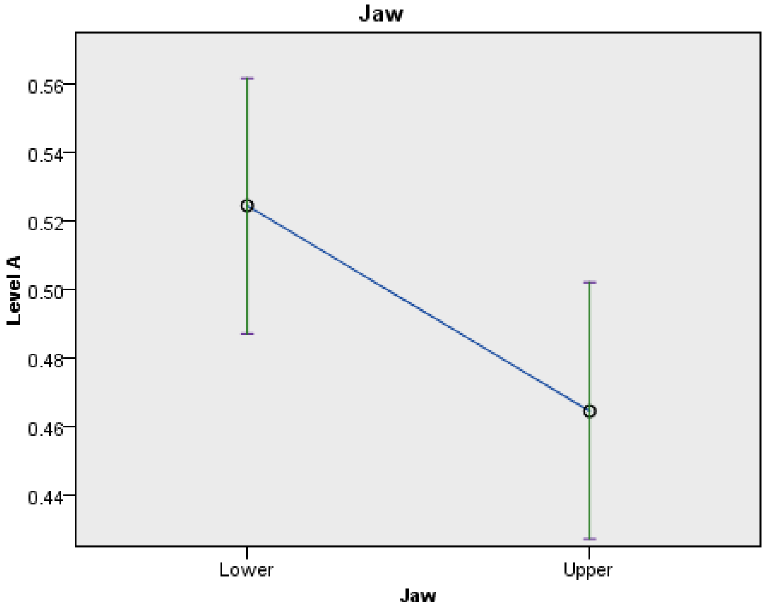

Generalized Linear Mixed Model

Generalized linear mixed models predict a continuous or categorical target based on one or more predictors. These models extend linear models to allow:

- Correlated data structures, including clustered and longitudinal designs.
- Non-normal targets such as binary, categorical, and count data.

- Data Structure
- Fields and Effects
  - Target
    - Target: ADi
    - Use number of trials as denominator: false
    - Customize reference category: true
    - Distribution: Binomial
    - Link function: Logit
  - Fixed Effects
  - Random Effects
- Build Options
- Training Summary

Model Summary

Target: Level B

**Target**		Level B
**Probability Distribution**		Binomial
**Link Function**		Logit
**Information Criterion**	**Akaike Corrected**	12,979.486
	**Bayesian**	12,985.267

Information criteria are based on the -2 log pseudo likelihood (12,977.484) and are used to compare models. Models with smaller information criterion values fit better. When comparing models using pseudo likelihood values, caution should be used because different data transformations may be used across the models.

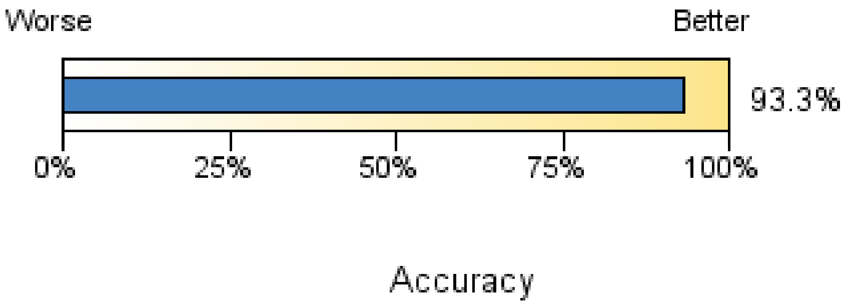

Data Structure

Target: Level B

	Subjects	Target
	Patient ID	Level B
Data For First Subject	1	0
Total Number of Levels	1979	

Classification

Target:Level B

Overall Percent Correct =93.3%

Observed	Predicted	
	1	0
1	0.0%	100.0%
0	0.0%	100.0%

Row Percent

100.00
80.00
60.00
40.00
20.00
0.00

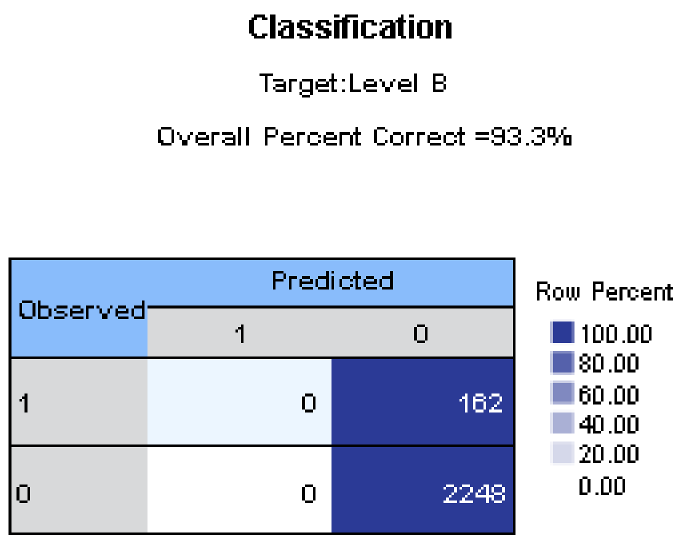
Classification
Target:Level B
Overall Percent Correct =93.3%
Predicted
Observed
1
0
1
0
162
0
0
2248
Row Percent
100.00
80.00
60.00
40.00
20.00
0.00

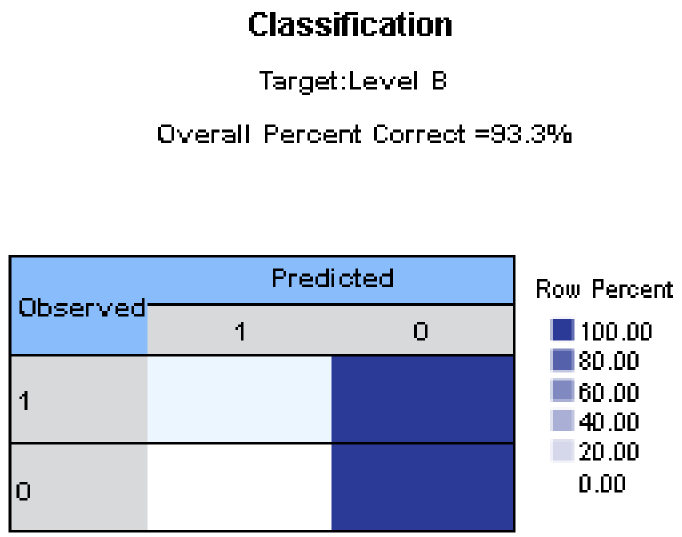
Classification
Target:Level B
Overall Percent Correct =93.3%
Predicted
Observed
1
0
1
0
Row Percent
100.00
80.00
60.00
40.00
20.00
0.00

Classification

Target:Level B

Overall Percent Correct =93.3%

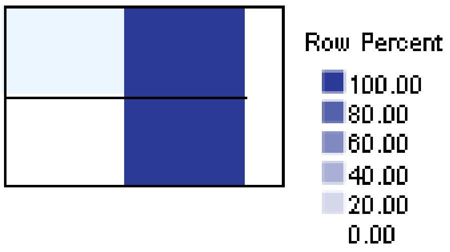

Fixed Effects

Target:Level B
Reference Category:0

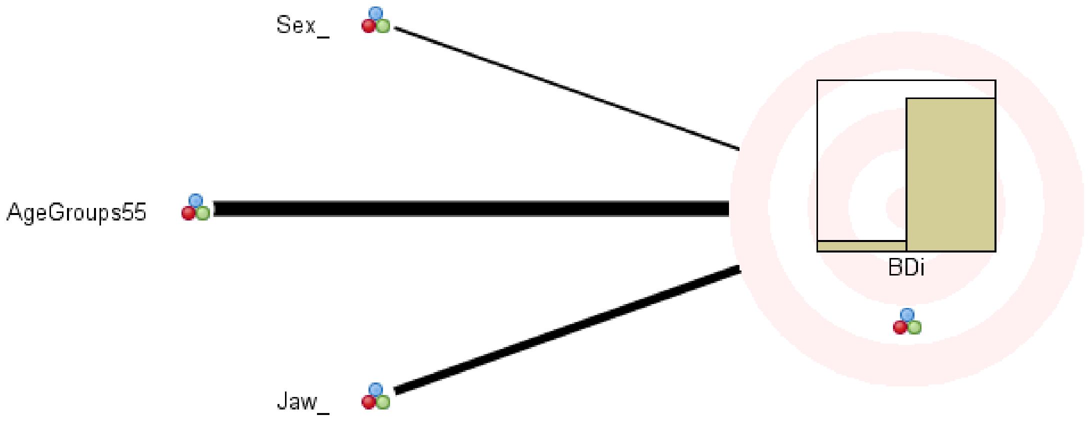

Fixed Effects

Target:Level B
Reference Category:0

Source	F	df1	df2	Sig.
Corrected Model	6.055	10	2,399	.000

Probability distribution:Binomial
Link function:Logit

Fixed Effects

Target:Level B
Reference Category:0

Source	F	df1	df2	Sig.
Corrected Model ▼	6.055	10	2,399	.000
Sex_	1.227	1	2,399	.268
AgeGroups55	5.684	8	2,399	.000
Jaw_	7.467	1	2,399	.006

Probability distribution:Binomial
Link function:Logit

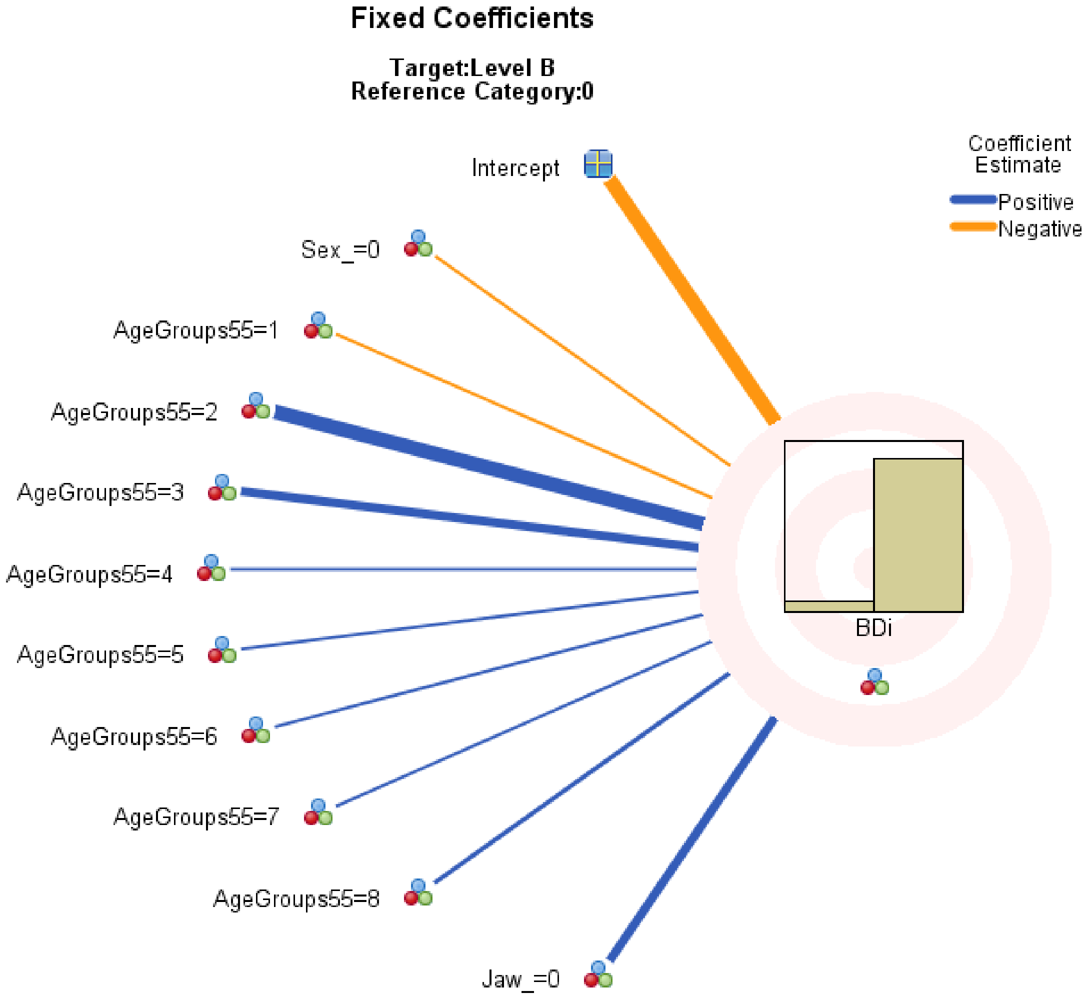

Fixed Coefficients
Target:Level B
Reference Category:0
Coefficient Estimate
Positive
Negative
Intercept
Sex_=0
AgeGroups55=1
AgeGroups55=2
AgeGroups55=3
AgeGroups55=4
AgeGroups55=5
AgeGroups55=6
AgeGroups55=7
AgeGroups55=8
Jaw_=0
BDi

Fixed Coefficients

Target:Level B
Reference Category:0

Model Term	Coefficient	Sig.	Exp(Coefficient)
Intercept	-3.220	.000	0.040
Sex_=0	-0.217	.268	0.805
Sex_=1	0[a]		
AgeGroups55=1	-0.450	.457	0.638
AgeGroups55=2	1.709	.000	5.524
AgeGroups55=3	0.885	.008	2.423
AgeGroups55=4	0.224	.519	1.251
AgeGroups55=5	0.383	.273	1.467
AgeGroups55=6	0.376	.363	1.457
AgeGroups55=7	0.248	.550	1.281
AgeGroups55=8	0.585	.166	1.796
AgeGroups55=9	0[a]		
Jaw_=0	0.429	.006	1.536
Jaw_=1	0[a]		

Probability distribution:Binomial
Link function:Logit

[a] This coefficient is set to zero because it is redundant.

Fixed Coefficients

Target:Level B
Reference Category:0

Model Term	Coefficient	Sig.
Intercept	-3.220	.000
Sex_=0	-0.217	.268
Sex_=1	0[a]	
AgeGroups55=1	-0.450	.457
AgeGroups55=2	1.709	.000
AgeGroups55=3	0.885	.008
AgeGroups55=4	0.224	.519
AgeGroups55=5	0.383	.273
AgeGroups55=6	0.376	.363
AgeGroups55=7	0.248	.550
AgeGroups55=8	0.585	.166
AgeGroups55=9	0[a]	
Jaw_=0	0.429	.006
Jaw_=1	0[a]	

Probability distribution:Binomial
Link function:Logit

[a] This coefficient is set to zero because it redundant.

Fixed Coefficients

Target:Level B
Reference Category:0

Model Term	Coefficient ▼	Std.Error	t	Sig.	Exp(Coefficient)
Intercept	-3.220	0.304	-10.600	.000	0.040
Sex_=0	-0.217	0.196	-1.108	.268	0.805
Sex_=1	0[a]				
AgeGroups55=1	-0.450	0.604	-0.744	.457	0.638
AgeGroups55=2	1.709	0.348	4.910	.000	5.524
AgeGroups55=3	0.885	0.334	2.652	.008	2.423
AgeGroups55=4	0.224	0.347	0.645	.519	1.251
AgeGroups55=5	0.383	0.350	1.096	.273	1.467
AgeGroups55=6	0.376	0.413	0.910	.363	1.457
AgeGroups55=7	0.248	0.414	0.598	.550	1.281
AgeGroups55=8	0.585	0.423	1.385	.166	1.796
AgeGroups55=9	0[a]				
Jaw_=0	0.429	0.157	2.733	.006	1.536
Jaw_=1	0[a]				

Probability distribution:Binomial
Link function:Logit

[a] This coefficient is set to zero because it is redundant.

Fixed Coefficients

Target:Level B
Reference Category:0

Model Term	95% Confidence Interval for Exp (Coefficient)	
	Lower	Upper
Intercept	0.022	0.072
Sex_=0	0.548	1.182
Sex_=1		
AgeGroups55=1	0.195	2.085
AgeGroups55=2	2.791	10.932
AgeGroups55=3	1.259	4.663
AgeGroups55=4	0.633	2.471
AgeGroups55=5	0.739	2.911
AgeGroups55=6	0.648	3.276
AgeGroups55=7	0.568	2.887
AgeGroups55=8	0.784	4.112
AgeGroups55=9		
Jaw_=0	1.129	2.091
Jaw_=1		

Probability distribution:Binomial
Link function:Logit

[a] This coefficient is set to zero because it is redundant.

Fixed Coefficients

Target:Level B
Reference Category:0

Model Term	Coefficient	Std.Error	t	Sig.	95% Confidence Interval	
					Lower	Upper
Intercept	-3.220	0.304	-10.600	.000	-3.816	-2.624
Sex_=0	-0.217	0.196	-1.108	.268	-0.601	0.167
Sex_=1	0[a]					
AgeGroups55=1	-0.450	0.604	-0.744	.457	-1.634	0.735
AgeGroups55=2	1.709	0.348	4.910	.000	1.026	2.392
AgeGroups55=3	0.885	0.334	2.652	.008	0.231	1.540
AgeGroups55=4	0.224	0.347	0.645	.519	-0.457	0.904
AgeGroups55=5	0.383	0.350	1.096	.273	-0.303	1.068
AgeGroups55=6	0.376	0.413	0.910	.363	-0.434	1.187
AgeGroups55=7	0.248	0.414	0.598	.550	-0.565	1.060
AgeGroups55=8	0.585	0.423	1.385	.166	-0.243	1.414
AgeGroups55=9	0[a]					
Jaw_=0	0.429	0.157	2.733	.006	0.121	0.738
Jaw_=1	0[a]					

Probability distribution:Binomial
Link function:Logit

[a] This coefficient is set to zero because it is redundant.

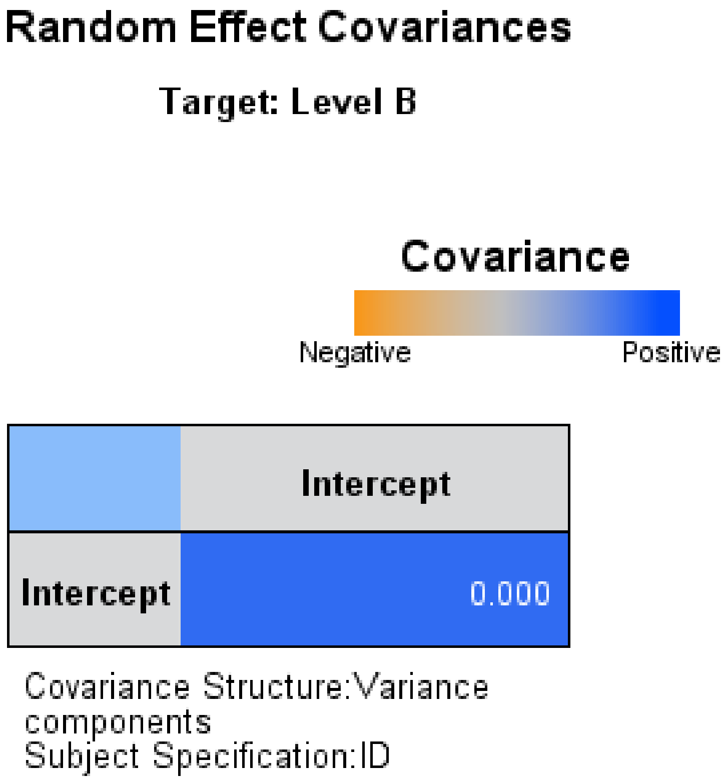
Random Effect Covariances
Target: Level B
Covariance
Negative
Positive
Intercept
Intercept
0.000
Covariance Structure:Variance components
Subject Specification:ID

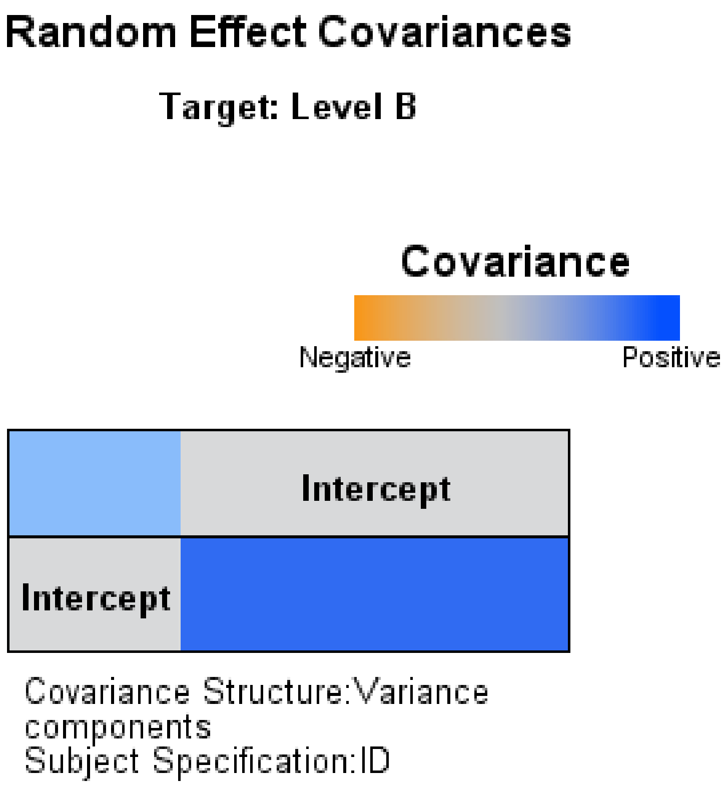
Random Effect Covariances
Target: Level B
Covariance
Negative
Positive
Intercept
Intercept
Covariance Structure:Variance components
Subject Specification:ID

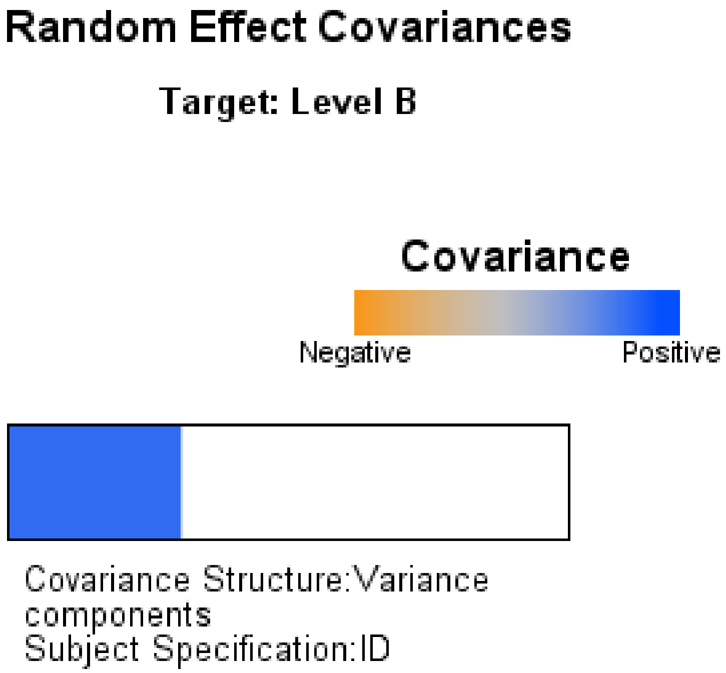
Random Effect Covariances
Target: Level B
Covariance
Negative
Positive
Covariance Structure:Variance components
Subject Specification:ID

Covariance Parameters

Target:Level B

**Covariance Parameters**	**Residual Effect**	0
	**Random Effects**	1
**Design Matrix Columns**	**Fixed Effects**	14
	**Random Effects**	1[a]
**Common Subjects**		1,979

Common subjects are based on the subject specifications for the residual and random effects and are used to chunk the data for better performance.

[a]This is the number of columns per common subject.

Residual Effect	Estimate	Std.Error	Z	Sig.	95% Confidence Interval	
					Lower	Upper
**Variance**	1.000	.	.	.	.	.

Covariance Structure:Scaled Identity
Subject Specification:(None)

Covariance Parameters

Target:Level B

Covariance Parameters	Residual Effect	0
	Random Effects	1
Design Matrix Columns	Fixed Effects	14
	Random Effects	1[a]
Common Subjects		1,979

Common subjects are based on the subject specifications for the residual and random effects and are used to chunk the data for better performance.

[a]This is the number of columns per common subject.

Random Effect	Estimate	Std.Error	Z	Sig.	95% Confidence Interval	
					Lower	Upper
Var(Intercept)	0.000[a]					

Covariance Structure:Variance components
Subject Specification:ID
[a]This parameter is redundant.

Estimated Means: Significant Effects

Target: Level B

Estimated means charts for significant effects (p<.05) are displayed. Up to ten effects are displayed, beginning with the top three-way effects. Effects shown contain categorical predictors only.

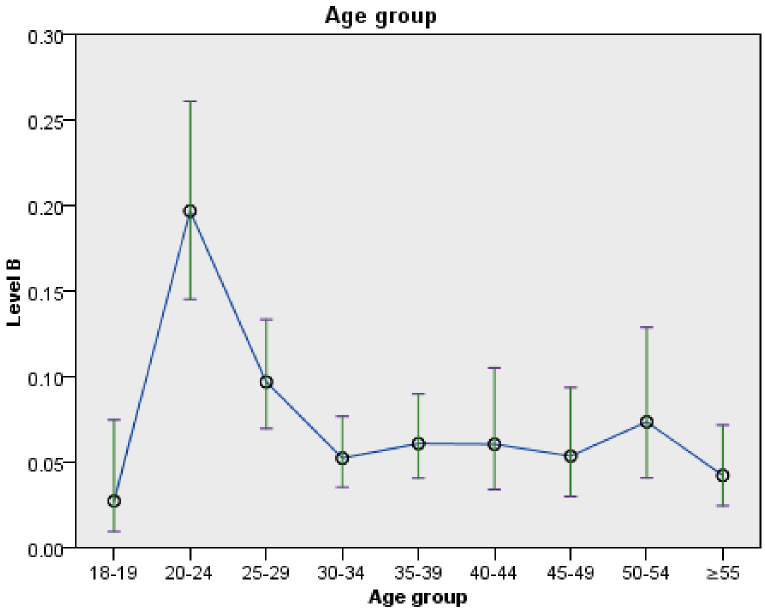

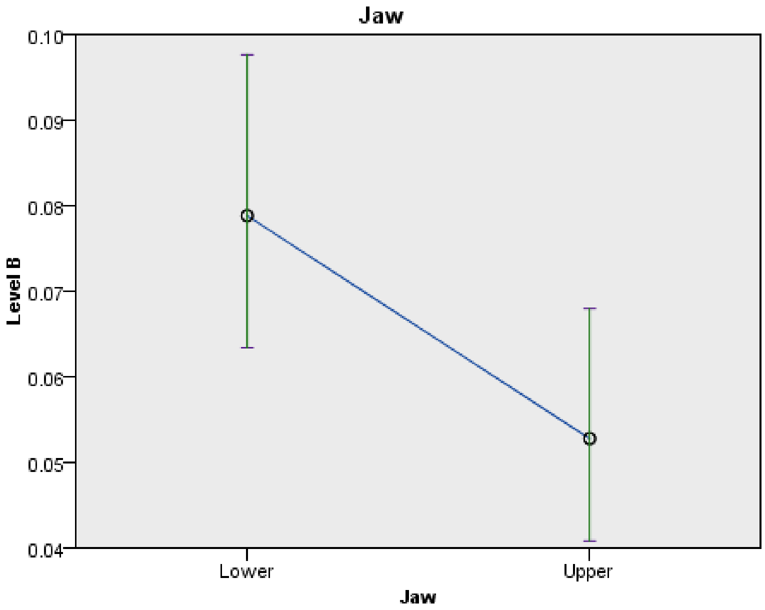

Generalized Linear Mixed Model

Generalized linear mixed models predict a continuous or categorical target based on one or more predictors. These models extend linear models to allow:

- Correlated data structures, including clustered and longitudinal designs.
- Non-normal targets such as binary, categorical, and count data.

- Data Structure
- Fields and Effects
  - Target
    - Target: BDi
    - Use number of trials as denominator: false
    - Customize reference category: true
    - Distribution: Binomial
    - Link function: Logit
  - Fixed Effects
  - Random Effects
- Build Options
- Training Summary

Model Summary

Target: Level C

**Target**		Level C
**Probability Distribution**		Binomial
**Link Function**		Logit
**Information Criterion**	**Akaike Corrected**	12,821.970
	**Bayesian**	12,827.751

Information criteria are based on the -2 log pseudo likelihood (12,819.968) and are used to compare models. Models with smaller information criterion values fit better. When comparing models using pseudo likelihood values, caution should be used because different data transformations may be used across the models.

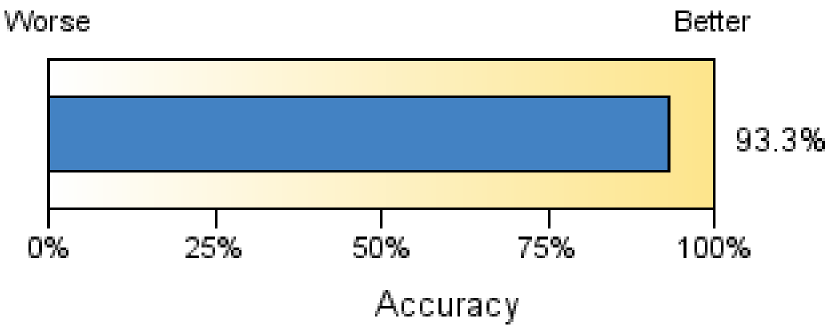

Data Structure

Target: Level C

	Subjects	Target
	Patient ID	Level C
Data For First Subject	1	0
Total Number of Levels	1979	

Classification

Target:Level C

Overall Percent Correct =93.3%

Observed	Predicted	
	1	0
1	0.0%	100.0%
0	0.0%	100.0%

Row Percent

100.00
80.00
60.00
40.00
20.00
0.00

Classification

Target:Level C

Overall Percent Correct =93.3%

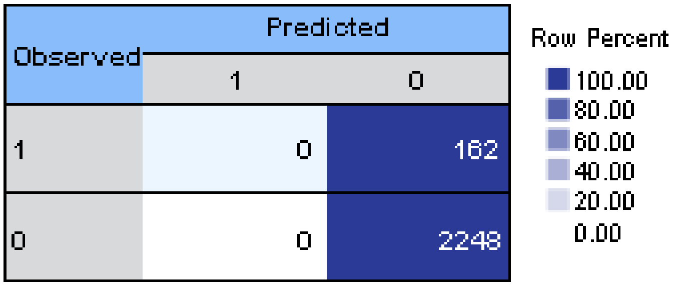

Classification

Target:Level C

Overall Percent Correct =93.3%

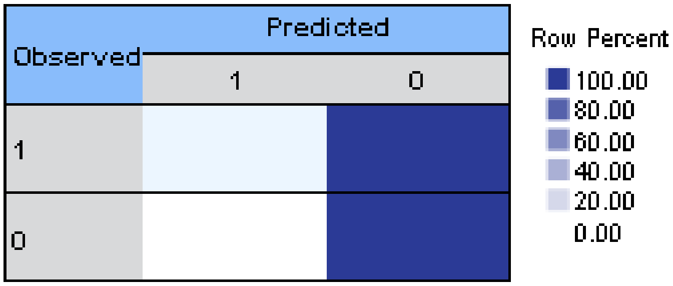

Classification

Target:Level C

Overall Percent Correct =93.3%

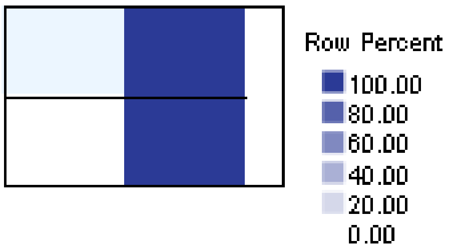

Fixed Effects

Target:Level C
Reference Category:0

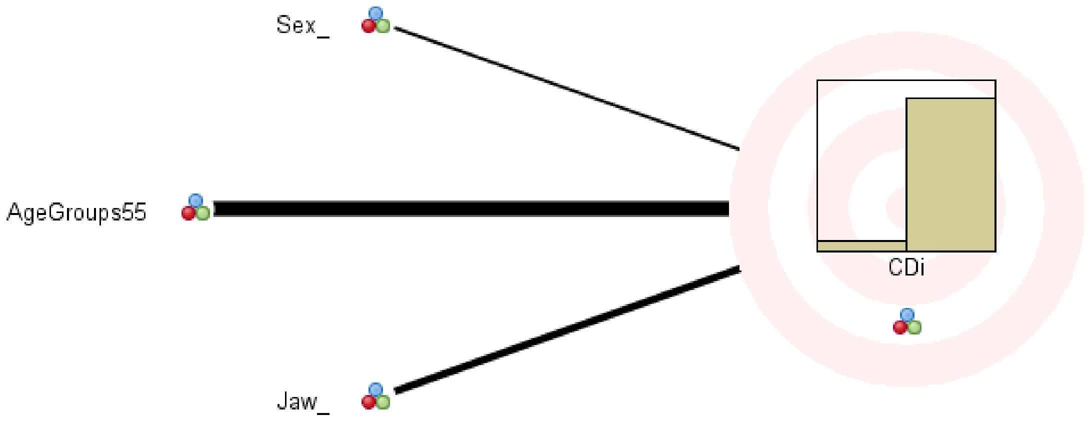

Fixed Effects

Target:Level C
Reference Category:0

Source	F	df1	df2	Sig.
Corrected Model	8.643	10	2,399	.000

Probability distribution:Binomial
Link function:Logit

Fixed Effects

Target:Level C
Reference Category:0

Source	F	df1	df2	Sig.
Corrected Model ▼	8.643	10	2,399	.000
Sex_	0.053	1	2,399	.817
AgeGroups55	8.673	8	2,399	.000
Jaw_	4.024	1	2,399	.045

Probability distribution:Binomial
Link function:Logit

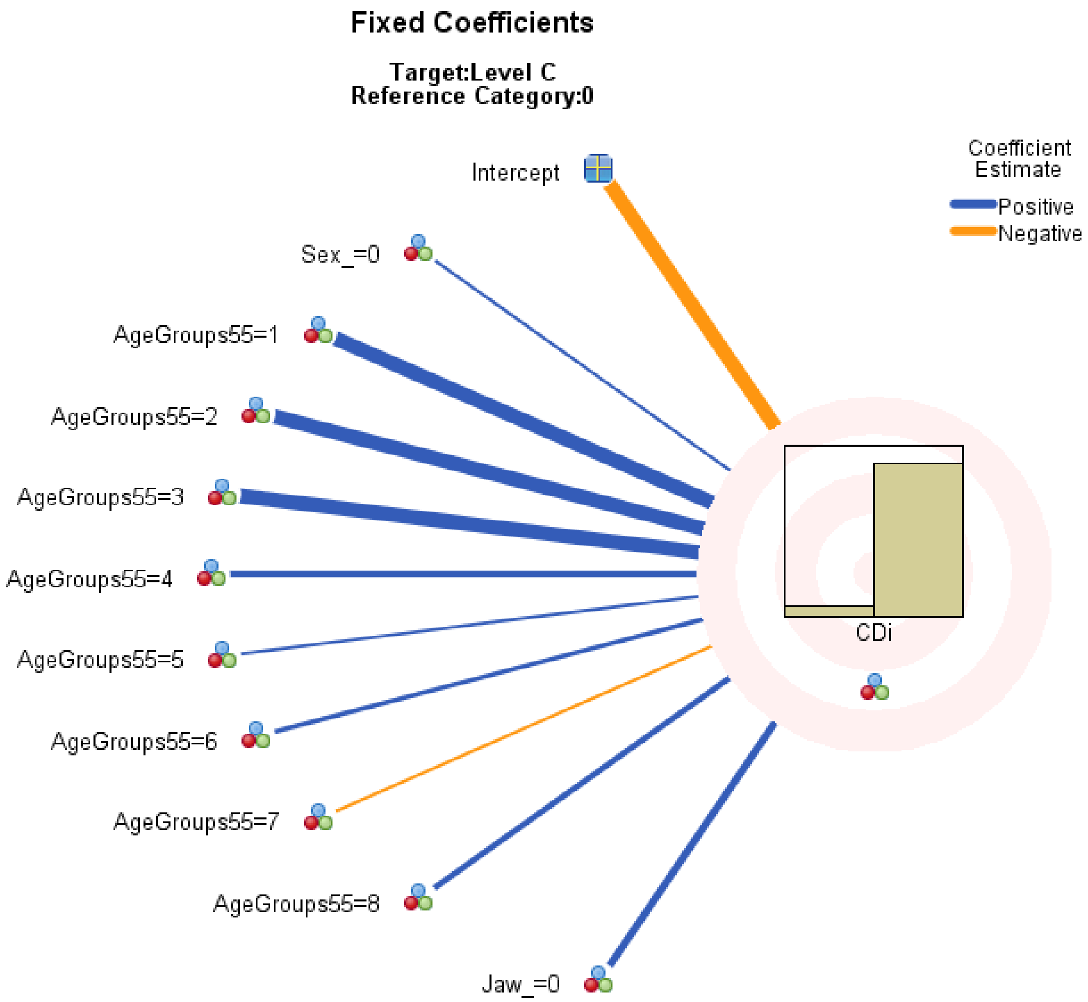
Fixed Coefficients
Target:Level C
Reference Category:0
Coefficient Estimate
Positive
Negative
Intercept
Sex_=0
AgeGroups55=1
AgeGroups55=2
AgeGroups55=3
AgeGroups55=4
AgeGroups55=5
AgeGroups55=6
AgeGroups55=7
AgeGroups55=8
Jaw_=0
CDi

Fixed Coefficients

Target:Level C
Reference Category:0

Model Term	Coefficient	Sig.	Exp(Coefficient)
Intercept	-3.697	.000	0.025
Sex_=0	0.043	.817	1.044
Sex_=1	0[a]		
AgeGroups55=1	1.811	.000	6.118
AgeGroups55=2	2.064	.000	7.879
AgeGroups55=3	1.587	.000	4.889
AgeGroups55=4	0.679	.091	1.972
AgeGroups55=5	0.433	.312	1.541
AgeGroups55=6	0.766	.101	2.151
AgeGroups55=7	-0.000	.999	1.000
AgeGroups55=8	0.926	.052	2.524
AgeGroups55=9	0[a]		
Jaw_=0	0.310	.045	1.364
Jaw_=1	0[a]		

Probability distribution:Binomial
Link function:Logit

[a] This coefficient is set to zero because it is redundant.

Fixed Coefficients

Target:Level C
Reference Category:0

Model Term	Coefficient	Sig.
Intercept	-3.697	.000
Sex_=0	0.043	.817
Sex_=1	0[a]	
AgeGroups55=1	1.811	.000
AgeGroups55=2	2.064	.000
AgeGroups55=3	1.587	.000
AgeGroups55=4	0.679	.091
AgeGroups55=5	0.433	.312
AgeGroups55=6	0.766	.101
AgeGroups55=7	-0.000	.999
AgeGroups55=8	0.926	.052
AgeGroups55=9	0[a]	
Jaw_=0	0.310	.045
Jaw_=1	0[a]	

Probability distribution:Binomial
Link function:Logit

[a]This coefficient is set to zero because it
redundant.

Fixed Coefficients

Target:Level C
Reference Category:0

Model Term	Coefficient	Std.Error	t	Sig.	Exp(Coefficient)
Intercept	-3.697	0.365	-10.117	.000	0.025
Sex_=0	0.043	0.187	0.231	.817	1.044
Sex_=1	0[a]				
AgeGroups55=1	1.811	0.427	4.239	.000	6.118
AgeGroups55=2	2.064	0.397	5.206	.000	7.879
AgeGroups55=3	1.587	0.383	4.140	.000	4.889
AgeGroups55=4	0.679	0.402	1.689	.091	1.972
AgeGroups55=5	0.433	0.428	1.010	.312	1.541
AgeGroups55=6	0.766	0.467	1.640	.101	2.151
AgeGroups55=7	-0.000	0.543	-0.001	.999	1.000
AgeGroups55=8	0.926	0.477	1.942	.052	2.524
AgeGroups55=9	0[a]				
Jaw_=0	0.310	0.155	2.006	.045	1.364
Jaw_=1	0[a]				

Probability distribution:Binomial
Link function:Logit

[a] This coefficient is set to zero because it is redundant.

Fixed Coefficients

Target:Level C
Reference Category:0

Model Term	95% Confidence Interval for Exp (Coefficient)	
	Lower	Upper
Intercept	0.012	0.051
Sex_=0	0.724	1.507
Sex_=1		
AgeGroups55=1	2.647	14.143
AgeGroups55=2	3.621	17.146
AgeGroups55=3	2.306	10.368
AgeGroups55=4	0.896	4.338
AgeGroups55=5	0.666	3.568
AgeGroups55=6	0.861	5.373
AgeGroups55=7	0.345	2.900
AgeGroups55=8	0.991	6.429
AgeGroups55=9		
Jaw_=0	1.007	1.848
Jaw_=1		

Probability distribution:Binomial
Link function:Logit

[a] This coefficient is set to zero because it is redundant.

Fixed Coefficients

Target:Level C
Reference Category:0

Model Term	Coefficient	Std.Error	t	Sig.	95% Confidence Interval	
					Lower	Upper
Intercept	-3.697	0.365	-10.117	.000	-4.413	-2.980
Sex_=0	0.043	0.187	0.231	.817	-0.323	0.410
Sex_=1	0[a]					
AgeGroups55=1	1.811	0.427	4.239	.000	0.973	2.649
AgeGroups55=2	2.064	0.397	5.206	.000	1.287	2.842
AgeGroups55=3	1.587	0.383	4.140	.000	0.835	2.339
AgeGroups55=4	0.679	0.402	1.689	.091	-0.109	1.467
AgeGroups55=5	0.433	0.428	1.010	.312	-0.407	1.272
AgeGroups55=6	0.766	0.467	1.640	.101	-0.150	1.681
AgeGroups55=7	-0.000	0.543	-0.001	.999	-1.065	1.065
AgeGroups55=8	0.926	0.477	1.942	.052	-0.009	1.861
AgeGroups55=9	0[a]					
Jaw_=0	0.310	0.155	2.006	.045	0.007	0.614
Jaw_=1	0[a]					

Probability distribution:Binomial
Link function:Logit

[a] This coefficient is set to zero because it is redundant.

Random Effect Covariances

Target: Level C

Covariance

Negative Positive

	Intercept
**Intercept**	0.000

Covariance Structure:Variance components
Subject Specification:ID

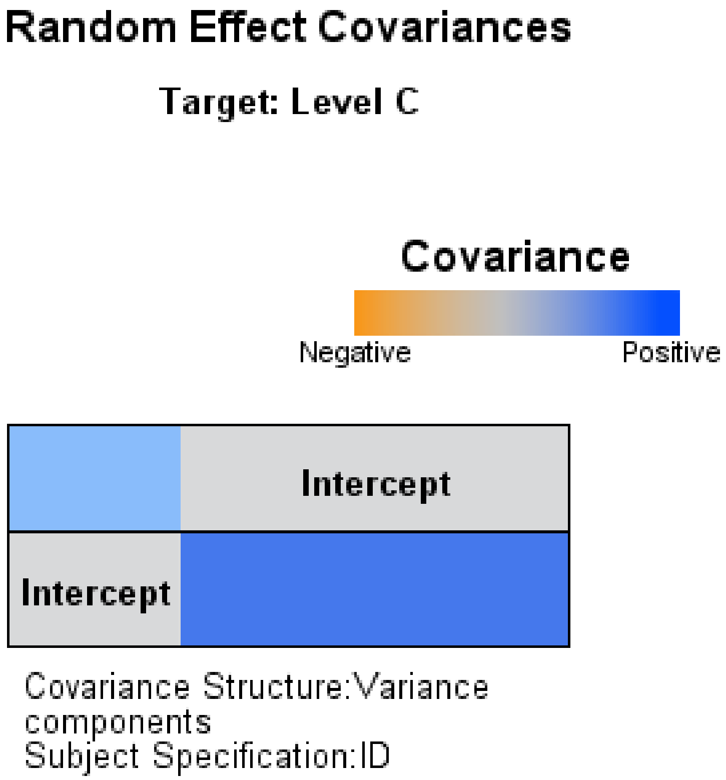
Random Effect Covariances
Target: Level C
Covariance
Negative
Positive
Intercept
Intercept
Covariance Structure:Variance components
Subject Specification:ID

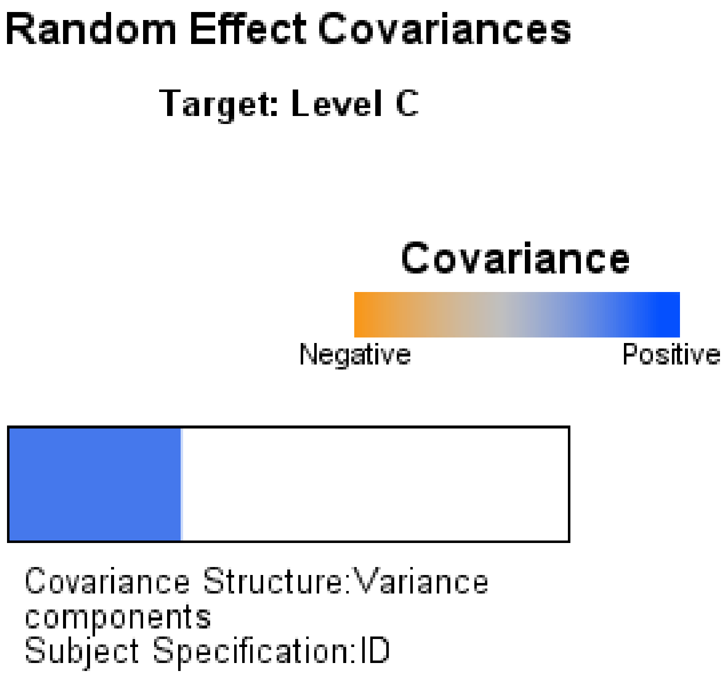
Random Effect Covariances
Target: Level C
Covariance
Negative
Positive
Covariance Structure:Variance components
Subject Specification:ID

Covariance Parameters

Target:Level C

**Covariance Parameters**	**Residual Effect**	0
	**Random Effects**	1
**Design Matrix Columns**	**Fixed Effects**	14
	**Random Effects**	1[a]
**Common Subjects**		1,979

Common subjects are based on the subject specifications for the residual and random effects and are used to chunk the data for better performance.

[a] This is the number of columns per common subject.

Residual Effect	Estimate	Std.Error	Z	Sig.	95% Confidence Interval	
					Lower	Upper
**Variance**	1.000	.	.	.	.	.

Covariance Structure:Scaled Identity
Subject Specification:(None)

Covariance Parameters

Target:Level C

**Covariance Parameters**	**Residual Effect**	0
	**Random Effects**	1
**Design Matrix Columns**	**Fixed Effects**	14
	**Random Effects**	1[a]
**Common Subjects**		1,979

Common subjects are based on the subject specifications for the residual and random effects and are used to chunk the data for better performance.

[a] This is the number of columns per common subject.

Random Effect	Estimate	Std.Error	Z	Sig.	95% Confidence Interval	
					Lower	Upper
**Var(Intercept)**	0.000[a]					

Covariance Structure:Variance components
Subject Specification:ID
[a] This parameter is redundant.

Estimated Means: Significant Effects

Target: Level C

Estimated means charts for significant effects (p<.05) are displayed. Up to ten effects are displayed, beginning with the top three-way effects. Effects shown contain categorical predictors only.

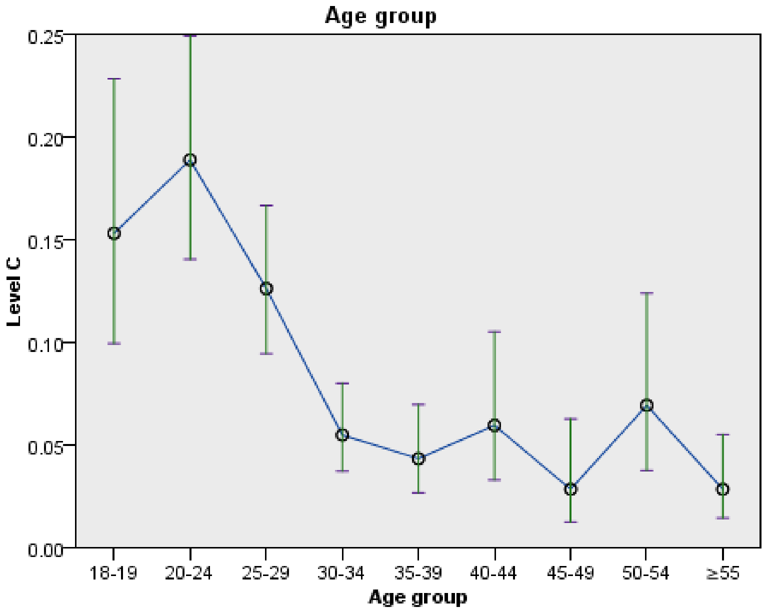

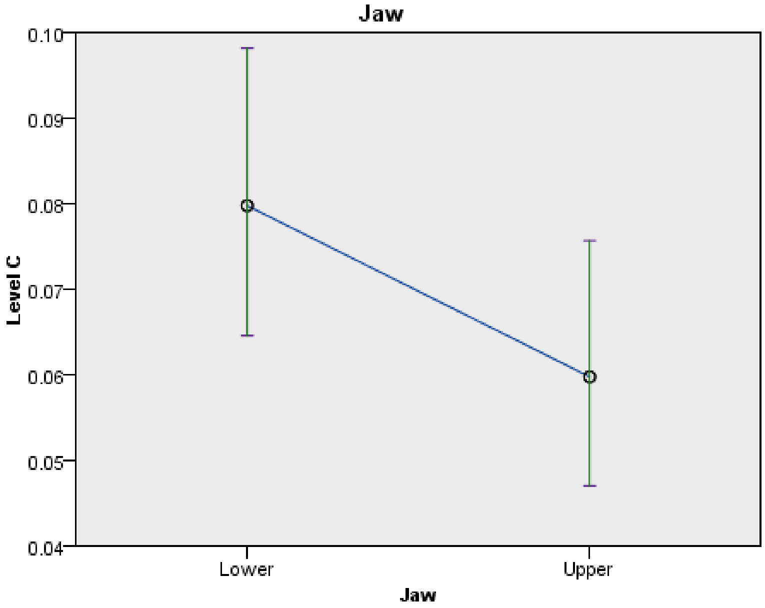

Generalized Linear Mixed Model

Generalized linear mixed models predict a continuous or categorical target based on one or more predictors. These models extend linear models to allow:

- Correlated data structures, including clustered and longitudinal designs.
- Non-normal targets such as binary, categorical, and count data.

- ⊞ Data Structure
- ⊟ Fields and Effects
  - ⊟ Target
    - Target: CDi
    - Use number of trials as denominator: false
    - ⊞ Customize reference category: true
    - Distribution: Binomial
    - Link function: Logit
  - ⊞ Fixed Effects
  - ⊞ Random Effects
- ⊞ Build Options
- ⊞ Training Summary

Model Summary

Target: Ma

**Target**		Ma
**Probability Distribution**		Binomial
**Link Function**		Logit
**Information Criterion**	**Akaike Corrected**	12,702.153
	**Bayesian**	12,707.931

Information criteria are based on the -2 log pseudo likelihood (12,700.152) and are used to compare models. Models with smaller information criterion values fit better. When comparing models using pseudo likelihood values, caution should be used because different data transformations may be used across the models.

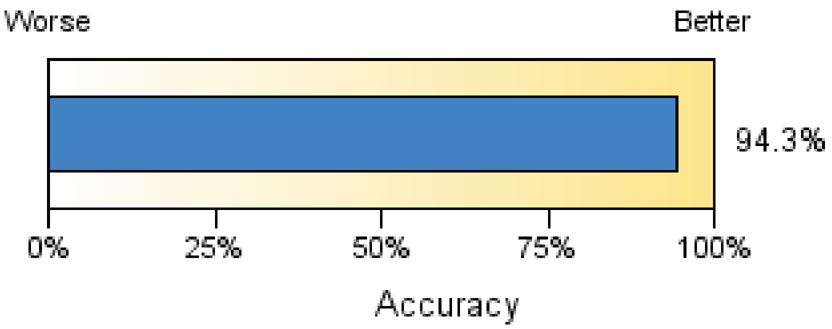

Data Structure

Target: Ma

	Subjects	Target
	Patient ID	Ma
Data For First Subject	1	0
Total Number of Levels	1970	

Classification

Target:Ma

Overall Percent Correct =94.3%

Observed	Predicted	
	1	0
1	0.0%	100.0%
0	0.0%	100.0%

Row Percent

- 100.00
- 80.00
- 60.00
- 40.00
- 20.00
- 0.00

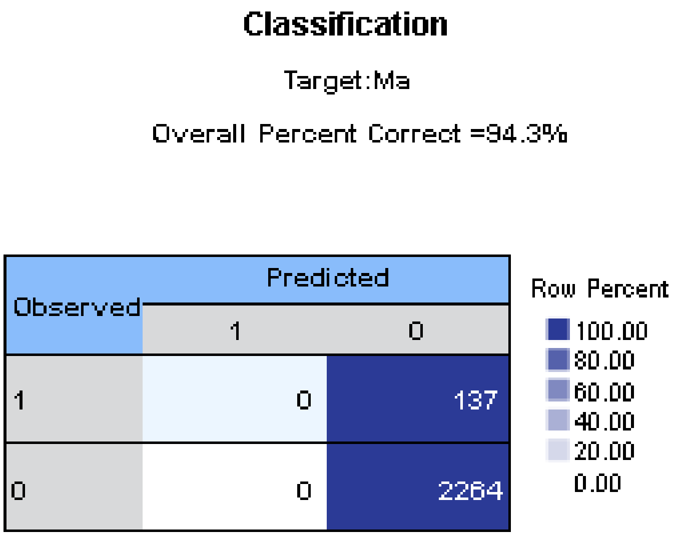
Classification
Target:Ma
Overall Percent Correct =94.3%
Predicted
Observed
1
0
1
0
137
0
0
2264
Row Percent
100.00
80.00
60.00
40.00
20.00
0.00

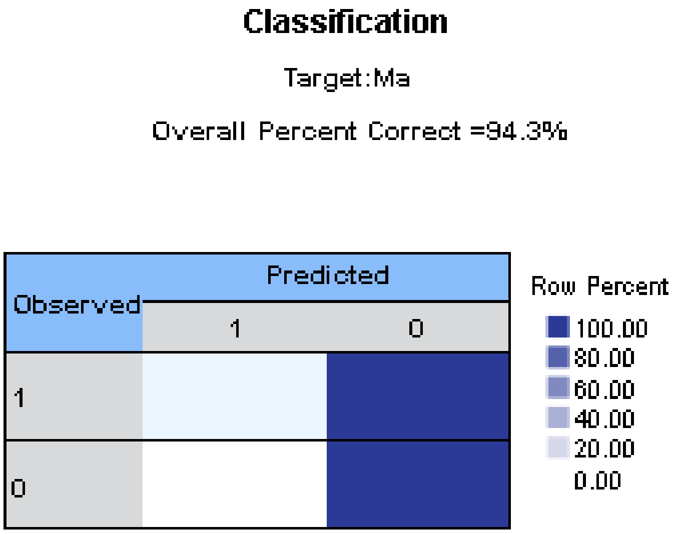
Classification
Target:Ma
Overall Percent Correct =94.3%
Predicted
Observed
1
0
1
0
Row Percent
100.00
80.00
60.00
40.00
20.00
0.00

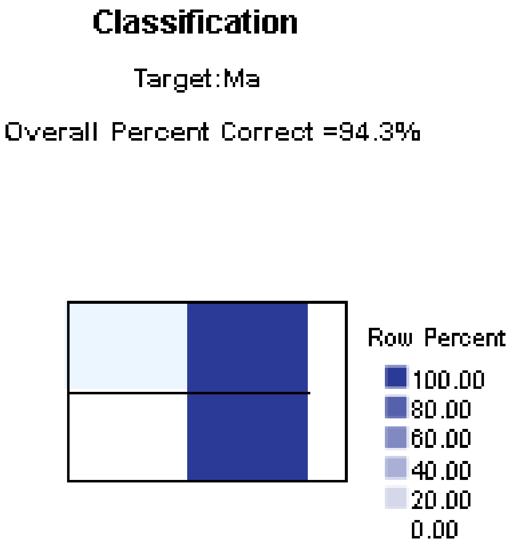
Classification
Target:Ma
Overall Percent Correct =94.3%
Row Percent
100.00
80.00
60.00
40.00
20.00
0.00

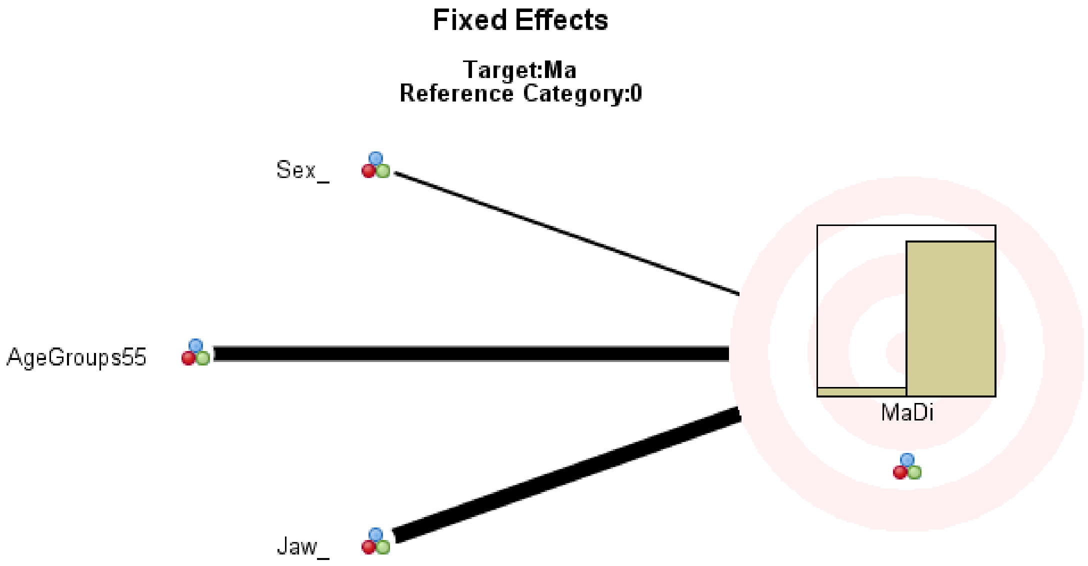
Fixed Effects
Target:Ma
Reference Category:0
Sex_
AgeGroups55
Jaw_
MaDi

Fixed Effects

Target:Ma
Reference Category:0

Source	F	df1	df2	Sig.
Corrected Model	8.037	10	2,390	.000

Probability distribution:Binomial
Link function:Logit

Fixed Effects

Target:Ma
Reference Category:0

Source	F	df1	df2	Sig.
Corrected Model ▼	8.037	10	2,390	.000
Sex_	2.246	1	2,390	.134
AgeGroups55	3.686	8	2,390	.000
Jaw_	51.913	1	2,390	.000

Probability distribution:Binomial
Link function:Logit

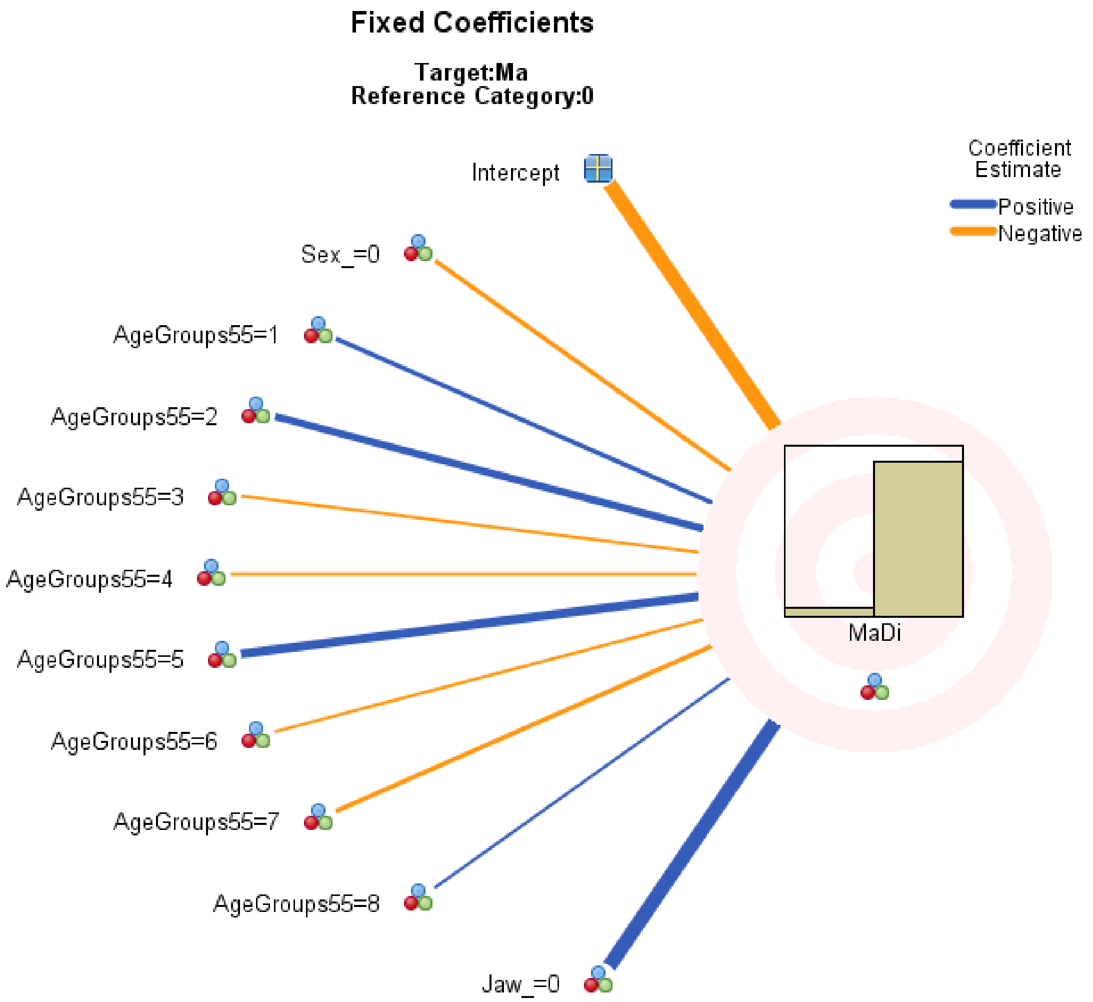
Fixed Coefficients
Target:Ma
Reference Category:0
Coefficient Estimate
Positive
Negative
Intercept
Sex_=0
AgeGroups55=1
AgeGroups55=2
AgeGroups55=3
AgeGroups55=4
AgeGroups55=5
AgeGroups55=6
AgeGroups55=7
AgeGroups55=8
Jaw_=0
MaDi

Fixed Coefficients

Target:Ma
Reference Category:0

Model Term	Coefficient	Sig.	Exp(Coefficient)
Intercept	-3.547	.000	0.029
Sex_=0	-0.294	.134	0.745
Sex_=1	0[a]		
AgeGroups55=1	0.578	.145	1.783
AgeGroups55=2	0.769	.028	2.157
AgeGroups55=3	-0.095	.786	0.910
AgeGroups55=4	-0.071	.828	0.932
AgeGroups55=5	0.808	.007	2.244
AgeGroups55=6	-0.049	.905	0.952
AgeGroups55=7	-0.637	.178	0.529
AgeGroups55=8	0.304	.455	1.355
AgeGroups55=9	0[a]		
Jaw_=0	1.355	.000	3.877
Jaw_=1	0[a]		

Probability distribution:Binomial
Link function:Logit

[a] This coefficient is set to zero because it is redundant.

Fixed Coefficients

Target:Ma
Reference Category:0

Model Term	Coefficient	Sig.
Intercept	-3.547	.000
Sex_=0	-0.294	.134
Sex_=1	0[a]	
AgeGroups55=1	0.578	.145
AgeGroups55=2	0.769	.028
AgeGroups55=3	-0.095	.786
AgeGroups55=4	-0.071	.828
AgeGroups55=5	0.808	.007
AgeGroups55=6	-0.049	.905
AgeGroups55=7	-0.637	.178
AgeGroups55=8	0.304	.455
AgeGroups55=9	0[a]	
Jaw_=0	1.355	.000
Jaw_=1	0[a]	

Probability distribution:Binomial
Link function:Logit

[a]This coefficient is set to zero because it
redundant.

Fixed Coefficients

Target:Ma
Reference Category:0

Model Term	Coefficient	Std.Error	t	Sig.	Exp(Coefficient)
Intercept	-3.547	0.297	-11.936	.000	0.029
Sex_=0	-0.294	0.196	-1.499	.134	0.745
Sex_=1	0[a]				
AgeGroups55=1	0.578	0.396	1.459	.145	1.783
AgeGroups55=2	0.769	0.350	2.195	.028	2.157
AgeGroups55=3	-0.095	0.350	-0.271	.786	0.910
AgeGroups55=4	-0.071	0.324	-0.217	.828	0.932
AgeGroups55=5	0.808	0.298	2.714	.007	2.244
AgeGroups55=6	-0.049	0.410	-0.119	.905	0.952
AgeGroups55=7	-0.637	0.473	-1.347	.178	0.529
AgeGroups55=8	0.304	0.406	0.748	.455	1.355
AgeGroups55=9	0[a]				
Jaw_=0	1.355	0.188	7.205	.000	3.877
Jaw_=1	0[a]				

Probability distribution:Binomial
Link function:Logit

[a] This coefficient is set to zero because it is redundant.

Fixed Coefficients

Target:Ma
Reference Category:0

Model Term	95% Confidence Interval for Exp (Coefficient)	
	Lower	Upper
Intercept	0.016	0.052
Sex_=0	0.507	1.095
Sex_=1		
AgeGroups55=1	0.820	3.878
AgeGroups55=2	1.085	4.286
AgeGroups55=3	0.458	1.805
AgeGroups55=4	0.493	1.760
AgeGroups55=5	1.251	4.025
AgeGroups55=6	0.426	2.129
AgeGroups55=7	0.209	1.337
AgeGroups55=8	0.611	3.003
AgeGroups55=9		
Jaw_=0	2.681	5.606
Jaw_=1		

Probability distribution:Binomial
Link function:Logit

[a] This coefficient is set to zero because it is redundant.

Fixed Coefficients

Target:Ma
Reference Category:0

Model Term	Coefficient	Std.Error	t	Sig.	95% Confidence Interval	
					Lower	Upper
Intercept	-3.547	0.297	-11.936	.000	-4.129	-2.964
Sex_=0	-0.294	0.196	-1.499	.134	-0.679	0.091
Sex_=1	0[a]					
AgeGroups55=1	0.578	0.396	1.459	.145	-0.199	1.355
AgeGroups55=2	0.769	0.350	2.195	.028	0.082	1.455
AgeGroups55=3	-0.095	0.350	-0.271	.786	-0.780	0.591
AgeGroups55=4	-0.071	0.324	-0.217	.828	-0.707	0.566
AgeGroups55=5	0.808	0.298	2.714	.007	0.224	1.392
AgeGroups55=6	-0.049	0.410	-0.119	.905	-0.853	0.755
AgeGroups55=7	-0.637	0.473	-1.347	.178	-1.563	0.290
AgeGroups55=8	0.304	0.406	0.748	.455	-0.493	1.100
AgeGroups55=9	0[a]					
Jaw_=0	1.355	0.188	7.205	.000	0.986	1.724
Jaw_=1	0[a]					

Probability distribution:Binomial
Link function:Logit

[a] This coefficient is set to zero because it is redundant.

Random Effect Covariances

Target: Ma

Covariance

Negative Positive

	Intercept
**Intercept**	0.000

Covariance Structure:Variance components
Subject Specification:ID

Random Effect Covariances

Target: Ma

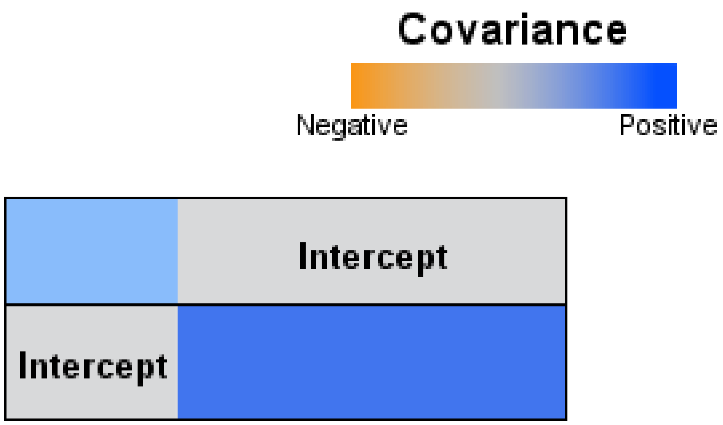

Covariance Structure:Variance components
Subject Specification:ID

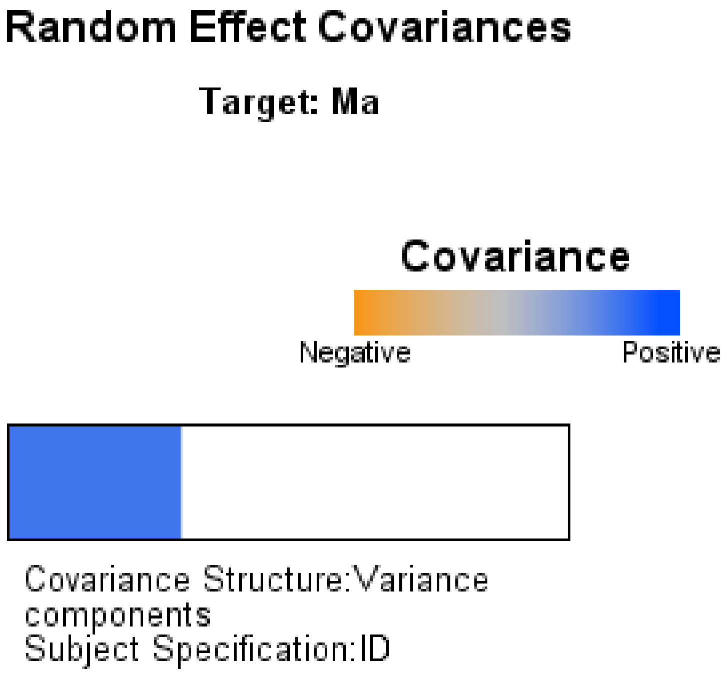
Random Effect Covariances
Target: Ma
Covariance
Negative
Positive
Covariance Structure:Variance components
Subject Specification:ID

Covariance Parameters

Target:Ma

**Covariance Parameters**	**Residual Effect**	0
	**Random Effects**	1
**Design Matrix Columns**	**Fixed Effects**	14
	**Random Effects**	1[a]
**Common Subjects**		1,970

Common subjects are based on the subject specifications for the residual and random effects and are used to chunk the data for better performance.

[a] This is the number of columns per common subject.

Residual Effect	Estimate	Std.Error	Z	Sig.	95% Confidence Interval	
					Lower	Upper
**Variance**	1.000	.	.	.	.	.

Covariance Structure:Scaled Identity
Subject Specification:(None)

Covariance Parameters

Target:Ma

**Covariance Parameters**	**Residual Effect**	0
	**Random Effects**	1
**Design Matrix Columns**	**Fixed Effects**	14
	**Random Effects**	1[a]
**Common Subjects**		1,970

Common subjects are based on the subject specifications for the residual and random effects and are used to chunk the data for better performance.

[a]This is the number of columns per common subject.

Random Effect	Estimate	Std.Error	Z	Sig.	95% Confidence Interval	
					Lower	Upper
**Var(Intercept)**	0.000[a]					

Covariance Structure:Variance components
Subject Specification:ID
[a]This parameter is redundant.

Estimated Means: Significant Effects

Target: Ma

Estimated means charts for significant effects (p<.05) are displayed. Up to ten effects are displayed, beginning with the top three-way effects. Effects shown contain categorical predictors only.

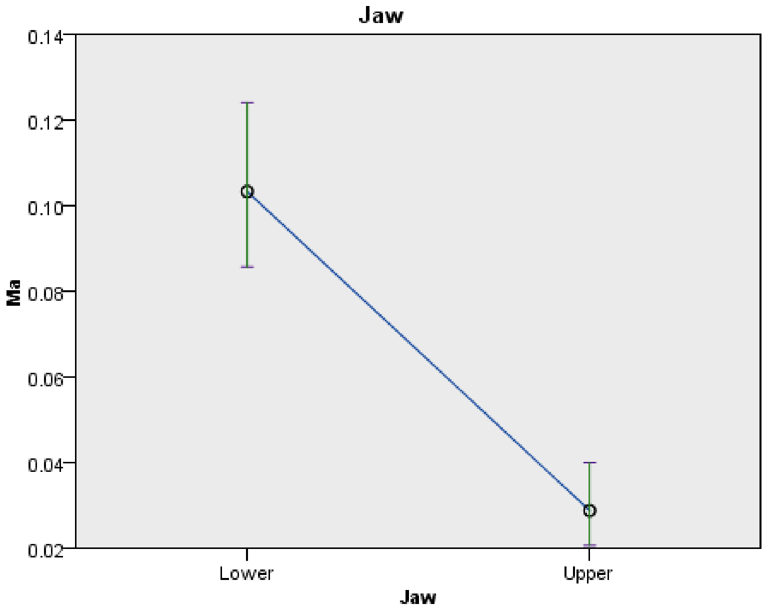

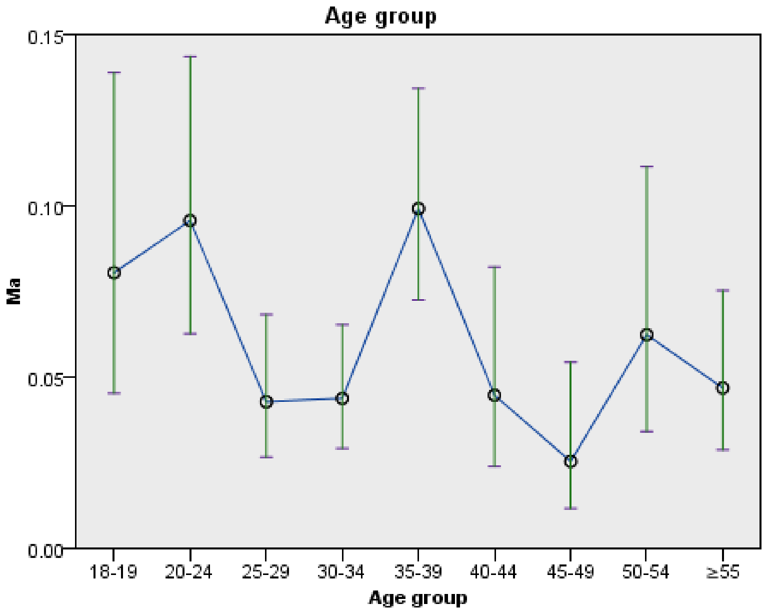

Generalized Linear Mixed Model

Generalized linear mixed models predict a continuous or categorical target based on one or more predictors. These models extend linear models to allow:

- Correlated data structures, including clustered and longitudinal designs.
- Non-normal targets such as binary, categorical, and count data.

- Data Structure
- Fields and Effects
  - Target
    - Target: MaDi
    - Use number of trials as denominator: false
    - Customize reference category: true
    - Distribution: Binomial
    - Link function: Logit
  - Fixed Effects
  - Random Effects
- Build Options
- Training Summary

Model Summary

Target: Da

**Target**		Da
**Probability Distribution**		Binomial
**Link Function**		Logit
**Information Criterion**	**Akaike Corrected**	13,149.904
	**Bayesian**	13,155.685

Information criteria are based on the -2 log pseudo likelihood (13,147.902) and are used to compare models. Models with smaller information criterion values fit better. When comparing models using pseudo likelihood values, caution should be used because different data transformations may be used across the models.

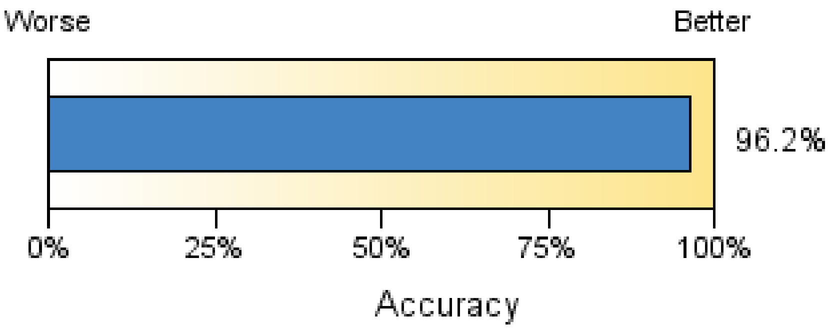

Data Structure

Target: Da

	Subjects	Target
	Patient ID	Da
Data For First Subject	1	0
Total Number of Levels	1979	

Classification

Target:Da

Overall Percent Correct =96.2%

Observed	Predicted	
	1	0
1	0.0%	100.0%
0	0.0%	100.0%

Row Percent

100.00
80.00
60.00
40.00
20.00
0.00

Classification

Target:Da

Overall Percent Correct =96.2%

Observed	Predicted	
	1	0
1	0	91
0	0	2319

Row Percent

- 100.00
- 80.00
- 60.00
- 40.00
- 20.00
- 0.00

Classification

Target:Da

Overall Percent Correct =96.2%

Observed	Predicted	
	1	0
1		
0		

Row Percent

- 100.00
- 80.00
- 60.00
- 40.00
- 20.00
- 0.00

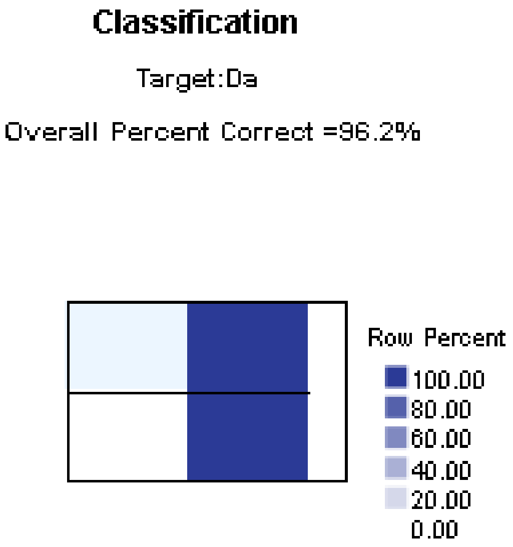
Classification
Target:Da
Overall Percent Correct =96.2%
Row Percent
100.00
80.00
60.00
40.00
20.00
0.00

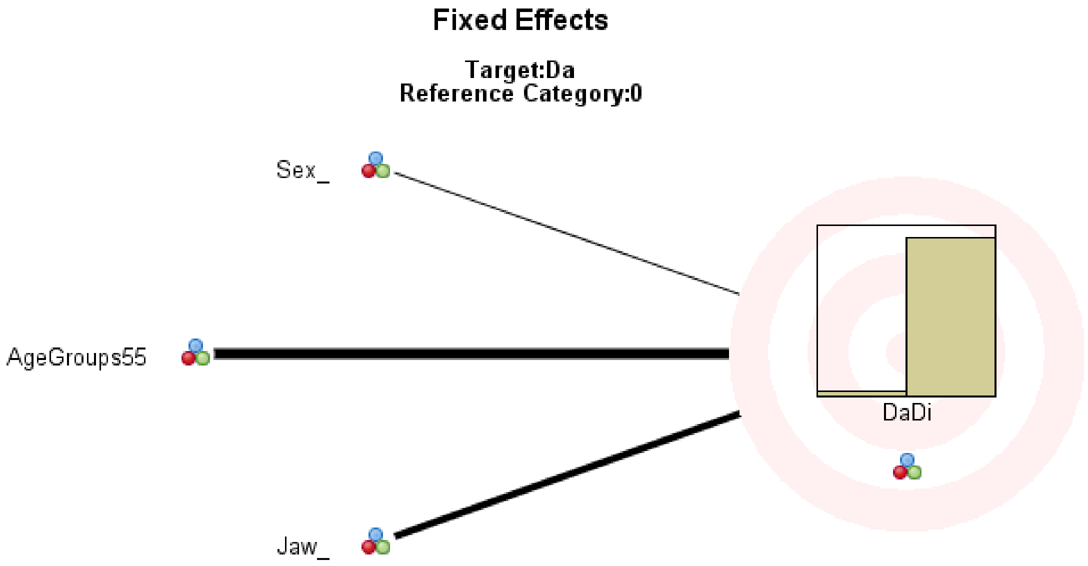
Fixed Effects
Target:Da
Reference Category:0
Sex_
AgeGroups55
Jaw_
DaDi

Fixed Effects

Target:Da
Reference Category:0

Source	F	df1	df2	Sig.
Corrected Model	3.109	10	2,399	.001

Probability distribution:Binomial
Link function:Logit

Fixed Effects

Target:Da
Reference Category:0

Source	F	df1	df2	Sig.
Corrected Model ▼	3.109	10	2,399	.001
Sex_	0.060	1	2,399	.807
AgeGroups55	3.253	8	2,399	.001
Jaw_	5.175	1	2,399	.023

Probability distribution:Binomial
Link function:Logit

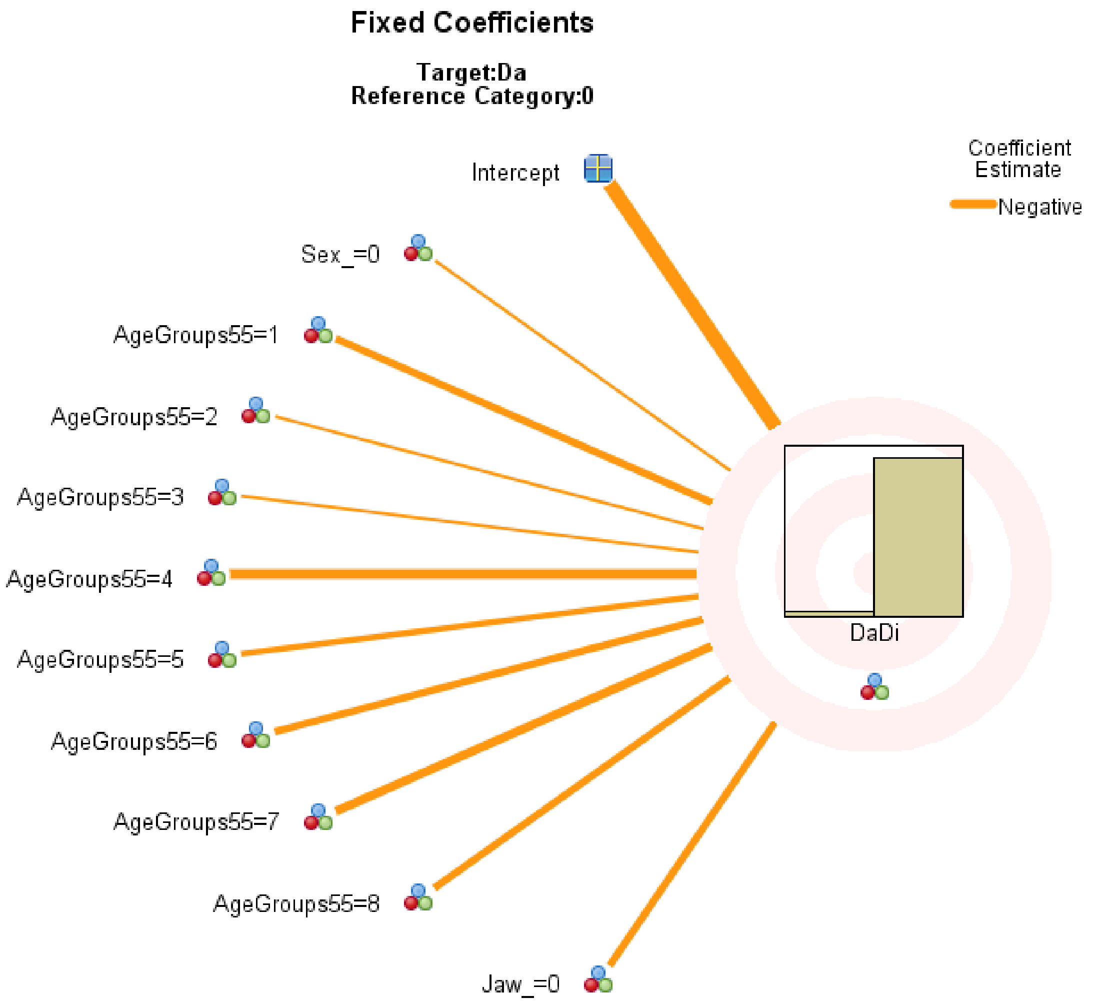
Fixed Coefficients
Target:Da
Reference Category:0
Coefficient Estimate
Negative
Intercept
Sex_=0
AgeGroups55=1
AgeGroups55=2
AgeGroups55=3
AgeGroups55=4
AgeGroups55=5
AgeGroups55=6
AgeGroups55=7
AgeGroups55=8
Jaw_=0
DaDi

Fixed Coefficients

Target:Da
Reference Category:0

Model Term	Coefficient	Sig.	Exp(Coefficient)
Intercept	-2.081	.000	0.125
Sex_=0	-0.052	.807	0.950
Sex_=1	0[a]		
AgeGroups55=1	-1.220	.033	0.295
AgeGroups55=2	-0.201	.551	0.818
AgeGroups55=3	-0.099	.725	0.906
AgeGroups55=4	-1.071	.001	0.343
AgeGroups55=5	-0.566	.062	0.568
AgeGroups55=6	-1.223	.012	0.294
AgeGroups55=7	-1.219	.009	0.296
AgeGroups55=8	-1.214	.024	0.297
AgeGroups55=9	0[a]		
Jaw_=0	-0.418	.023	0.659
Jaw_=1	0[a]		

Probability distribution:Binomial
Link function:Logit

[a] This coefficient is set to zero because it is redundant.

Fixed Coefficients

Target:Da
Reference Category:0

Model Term	Coefficient	Sig.
Intercept	-2.081	.000
Sex_=0	-0.052	.807
Sex_=1	0[a]	
AgeGroups55=1	-1.220	.033
AgeGroups55=2	-0.201	.551
AgeGroups55=3	-0.099	.725
AgeGroups55=4	-1.071	.001
AgeGroups55=5	-0.566	.062
AgeGroups55=6	-1.223	.012
AgeGroups55=7	-1.219	.009
AgeGroups55=8	-1.214	.024
AgeGroups55=9	0[a]	
Jaw_=0	-0.418	.023
Jaw_=1	0[a]	

Probability distribution:Binomial
Link function:Logit

[a]This coefficient is set to zero because it redundant.

Fixed Coefficients

Target:Da
Reference Category:0

Model Term	Coefficient	Std.Error	t	Sig.	Exp(Coefficient)
Intercept	-2.081	0.227	-9.154	.000	0.125
Sex_=0	-0.052	0.211	-0.245	.807	0.950
Sex_=1	0[a]				
AgeGroups55=1	-1.220	0.571	-2.135	.033	0.295
AgeGroups55=2	-0.201	0.337	-0.597	.551	0.818
AgeGroups55=3	-0.099	0.281	-0.352	.725	0.906
AgeGroups55=4	-1.071	0.327	-3.272	.001	0.343
AgeGroups55=5	-0.566	0.303	-1.870	.062	0.568
AgeGroups55=6	-1.223	0.485	-2.519	.012	0.294
AgeGroups55=7	-1.219	0.463	-2.632	.009	0.296
AgeGroups55=8	-1.214	0.536	-2.265	.024	0.297
AgeGroups55=9	0[a]				
Jaw_=0	-0.418	0.184	-2.275	.023	0.659
Jaw_=1	0[a]				

Probability distribution:Binomial
Link function:Logit

[a] This coefficient is set to zero because it is redundant.

Fixed Coefficients

Target:Da
Reference Category:0

Model Term	95% Confidence Interval for Exp (Coefficient)	
	Lower	Upper
Intercept	0.080	0.195
Sex_=0	0.628	1.437
Sex_=1		
AgeGroups55=1	0.096	0.905
AgeGroups55=2	0.422	1.584
AgeGroups55=3	0.523	1.571
AgeGroups55=4	0.180	0.651
AgeGroups55=5	0.313	1.028
AgeGroups55=6	0.114	0.763
AgeGroups55=7	0.119	0.733
AgeGroups55=8	0.104	0.850
AgeGroups55=9		
Jaw_=0	0.459	0.944
Jaw_=1		

Probability distribution:Binomial
Link function:Logit

[a] This coefficient is set to zero because it is redundant.

Fixed Coefficients

Target:Da
Reference Category:0

Model Term	Coefficient	Std.Error	t	Sig.	95% Confidence Interval	
					Lower	Upper
Intercept	-2.081	0.227	-9.154	.000	-2.527	-1.635
Sex_=0	-0.052	0.211	-0.245	.807	-0.466	0.362
Sex_=1	0[a]					
AgeGroups55=1	-1.220	0.571	-2.135	.033	-2.341	-0.100
AgeGroups55=2	-0.201	0.337	-0.597	.551	-0.863	0.460
AgeGroups55=3	-0.099	0.281	-0.352	.725	-0.649	0.452
AgeGroups55=4	-1.071	0.327	-3.272	.001	-1.712	-0.429
AgeGroups55=5	-0.566	0.303	-1.870	.062	-1.160	0.028
AgeGroups55=6	-1.223	0.485	-2.519	.012	-2.174	-0.271
AgeGroups55=7	-1.219	0.463	-2.632	.009	-2.127	-0.311
AgeGroups55=8	-1.214	0.536	-2.265	.024	-2.266	-0.163
AgeGroups55=9	0[a]					
Jaw_=0	-0.418	0.184	-2.275	.023	-0.778	-0.058
Jaw_=1	0[a]					

Probability distribution:Binomial
Link function:Logit

[a] This coefficient is set to zero because it is redundant.

Random Effect Covariances

Target: Da

Covariance

Negative Positive

	Intercept
**Intercept**	0.000

Covariance Structure:Variance components
Subject Specification:ID

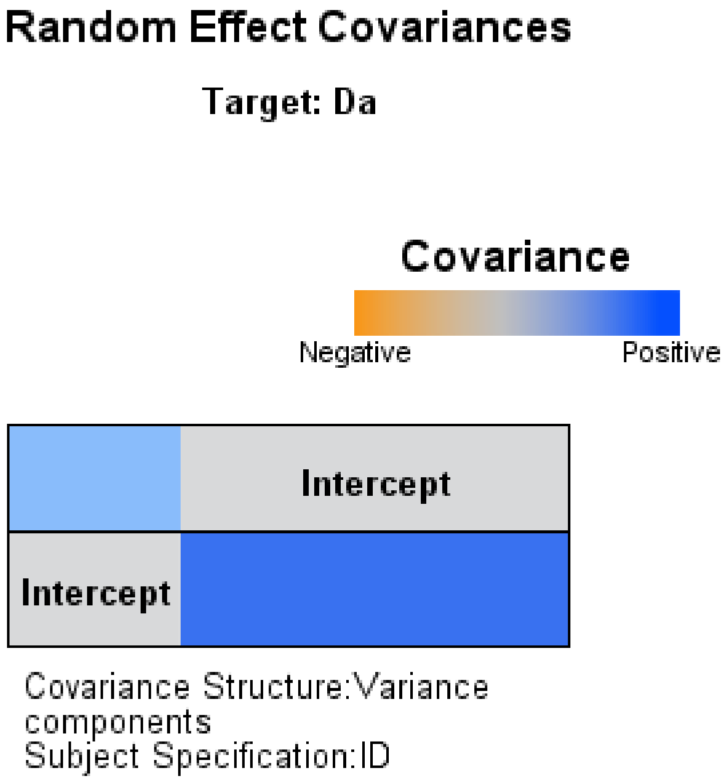
Random Effect Covariances
Target: Da
Covariance
Negative
Positive
Intercept
Intercept
Covariance Structure:Variance components
Subject Specification:ID

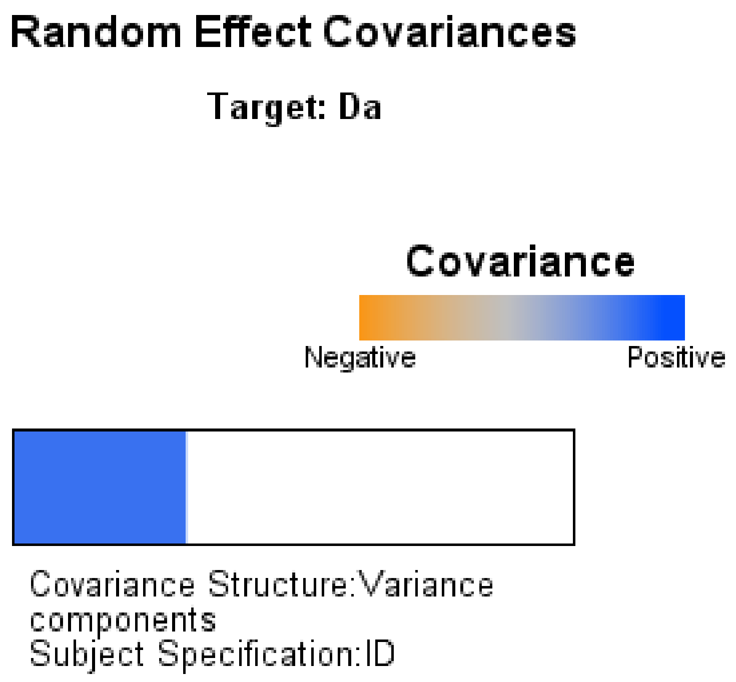
Random Effect Covariances
Target: Da
Covariance
Negative
Positive
Covariance Structure:Variance components
Subject Specification:ID

Covariance Parameters

Target:Da

**Covariance Parameters**	**Residual Effect**	0
	**Random Effects**	1
**Design Matrix Columns**	**Fixed Effects**	14
	**Random Effects**	1[a]
**Common Subjects**		1,979

Common subjects are based on the subject specifications for the residual and random effects and are used to chunk the data for better performance.

[a]This is the number of columns per common subject.

Residual Effect	Estimate	Std.Error	Z	Sig.	95% Confidence Interval	
					Lower	Upper
**Variance**	1.000	.	.	.	.	.

Covariance Structure:Scaled Identity
Subject Specification:(None)

Estimated Means: Significant Effects

Target: Da

Estimated means charts for significant effects (p<.05) are displayed. Up to ten effects are displayed, beginning with the top three-way effects. Effects shown contain categorical predictors only.

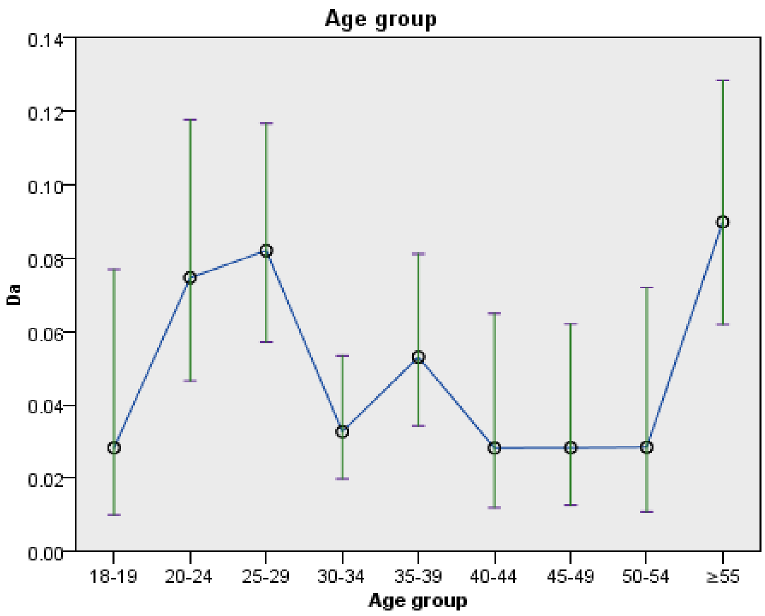

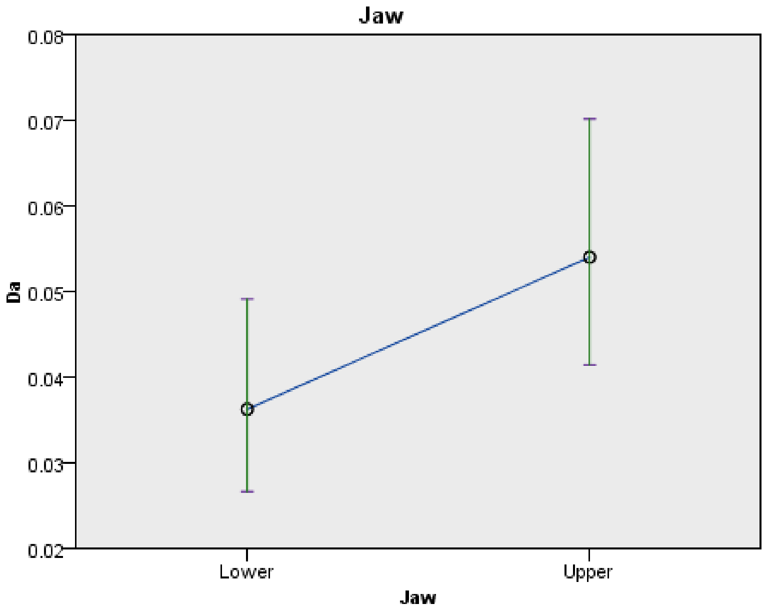

Generalized Linear Mixed Model

Generalized linear mixed models predict a continuous or categorical target based on one or more predictors. These models extend linear models to allow:

- Correlated data structures, including clustered and longitudinal designs.
- Non-normal targets such as binary, categorical, and count data.

- ⊞ Data Structure
- ⊟ Fields and Effects
  - ⊟ Target
    - Target: DaDi
    - Use number of trials as denominator: false
    - ⊞ Customize reference category: true
    - Distribution: Binomial
    - Link function: Logit
  - ⊞ Fixed Effects
  - ⊞ Random Effects
- ⊞ Build Options
- ⊞ Training Summary

Model Summary

Target: V

**Target**		V
**Probability Distribution**		Binomial
**Link Function**		Logit
**Information Criterion**	**Akaike Corrected**	10,563.898
	**Bayesian**	10,569.679

Information criteria are based on the -2 log pseudo likelihood (10,561.896) and are used to compare models. Models with smaller information criterion values fit better. When comparing models using pseudo likelihood values, caution should be used because different data transformations may be used across the models.

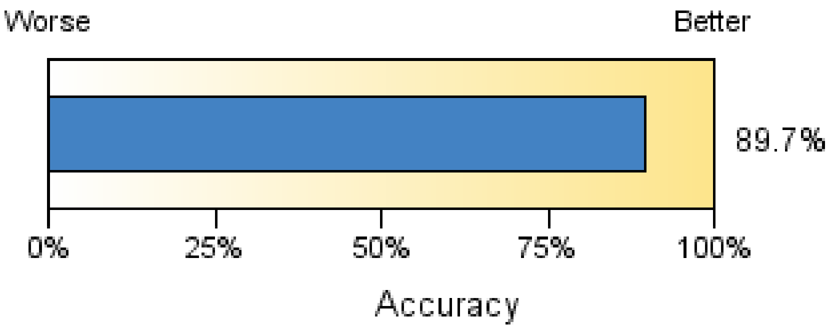

Data Structure

Target: V

	Subjects	Target
	Patient ID	V
Data For First Subject	1	0
Total Number of Levels	1979	

Classification

Target:V

Overall Percent Correct =89.7%

Observed	Predicted	
	1	0
1	94.1%	5.9%
0	15.4%	84.6%

Row Percent

100.00
80.00
60.00
40.00
20.00
0.00

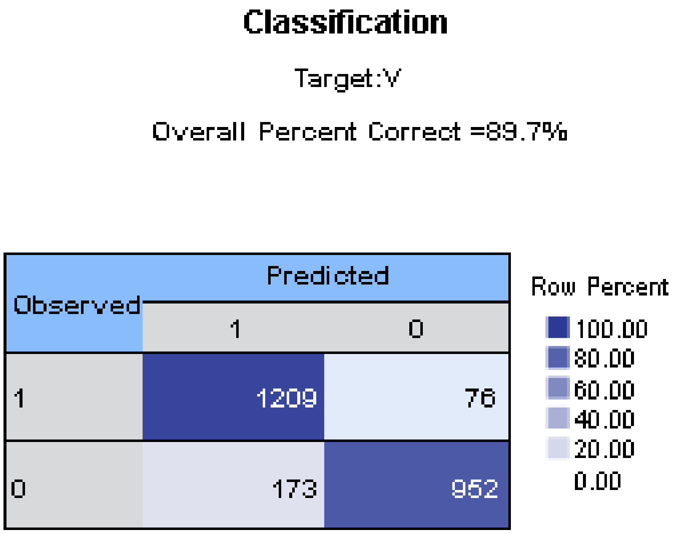
Classification
Target:V
Overall Percent Correct =89.7%
Predicted
Observed
1
0
1
1209
76
0
173
952
Row Percent
100.00
80.00
60.00
40.00
20.00
0.00

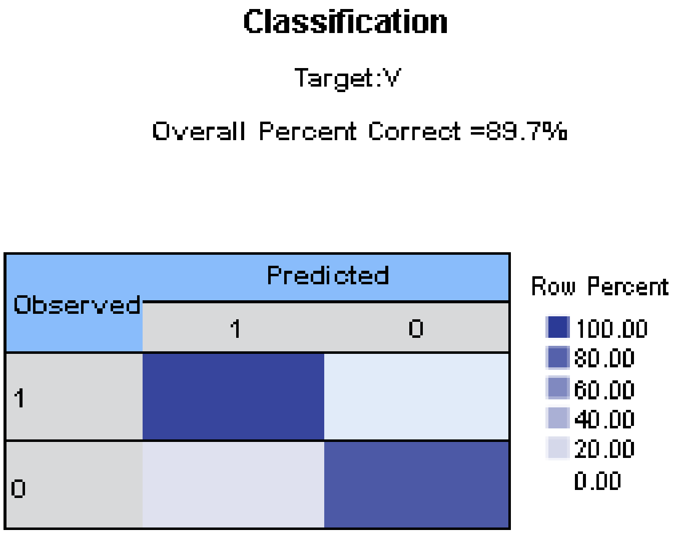
Classification
Target:V
Overall Percent Correct =89.7%
Predicted
Observed
1
0
1
0
Row Percent
100.00
80.00
60.00
40.00
20.00
0.00

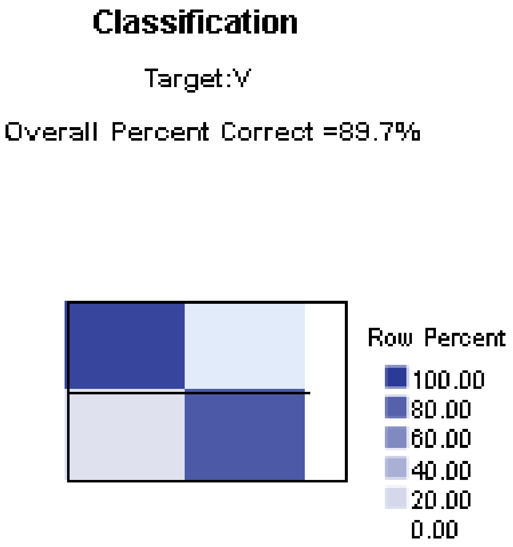
Classification
Target:V
Overall Percent Correct =89.7%
Row Percent
100.00
80.00
60.00
40.00
20.00
0.00

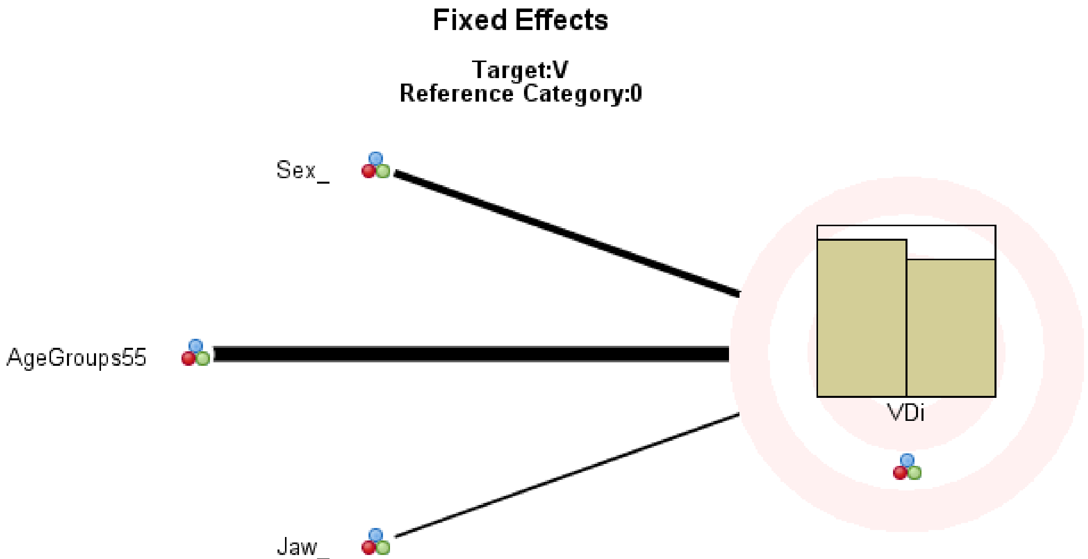
Fixed Effects
Target:V
Reference Category:0
Sex_
AgeGroups55
Jaw_
VDi

Fixed Effects

Target:V
Reference Category:0

Source	F	df1	df2	Sig.
Corrected Model	16.574	10	2,399	.000

Probability distribution:Binomial
Link function:Logit

Fixed Effects

Target:V
Reference Category:0

Source	F	df1	df2	Sig.
Corrected Model ▼	16.574	10	2,399	.000
Sex_	5.485	1	2,399	.019
AgeGroups55	20.176	8	2,399	.000
Jaw_	1.046	1	2,399	.306

Probability distribution:Binomial
Link function:Logit

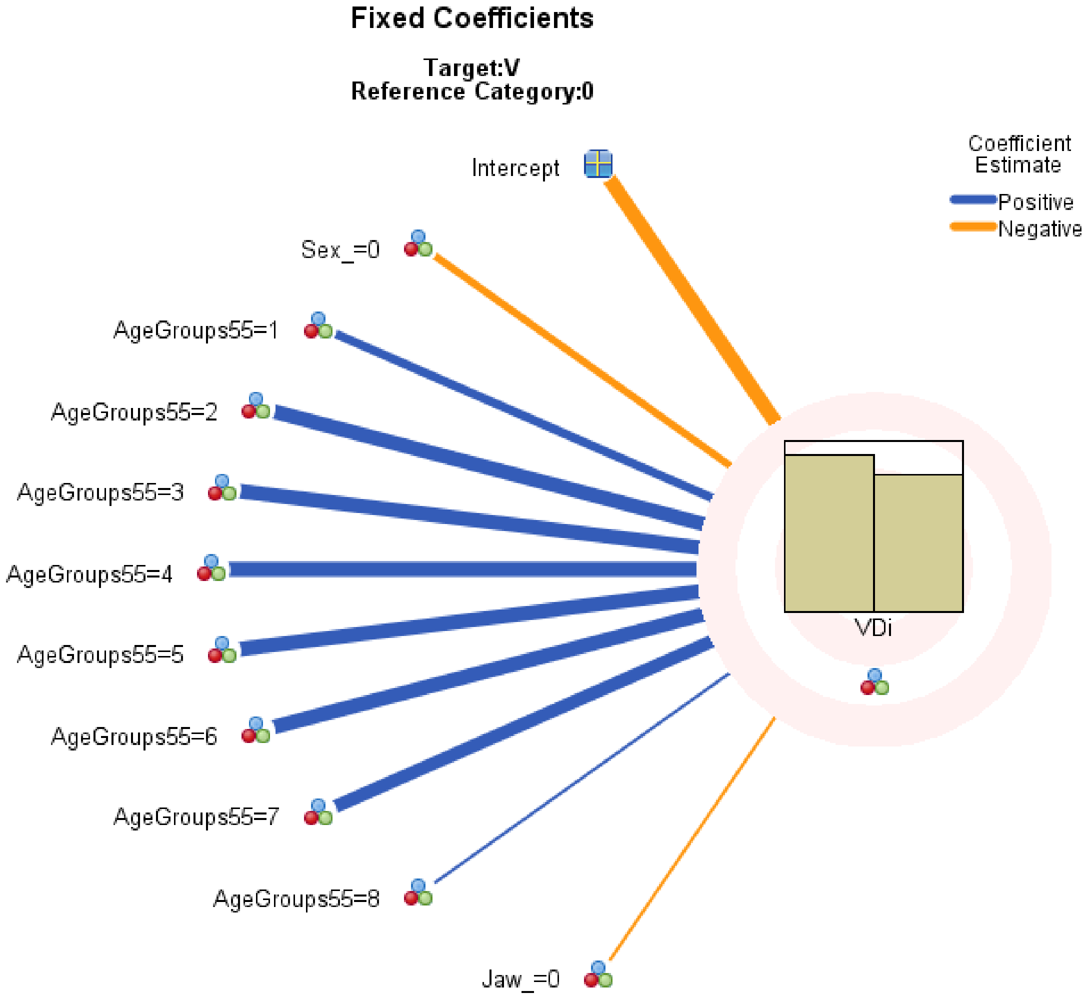
Fixed Coefficients
Target:V
Reference Category:0
Coefficient Estimate
Positive
Negative
Intercept
Sex_=0
AgeGroups55=1
AgeGroups55=2
AgeGroups55=3
AgeGroups55=4
AgeGroups55=5
AgeGroups55=6
AgeGroups55=7
AgeGroups55=8
Jaw_=0
VDi

Fixed Coefficients

Target:V
Reference Category:0

Model Term	Coefficient	Sig.	Exp(Coefficient)
Intercept	-1.017	.000	0.362
Sex_=0	-0.273	.019	0.761
Sex_=1	0[a]		
AgeGroups55=1	0.718	.008	2.051
AgeGroups55=2	1.714	.000	5.549
AgeGroups55=3	1.766	.000	5.849
AgeGroups55=4	1.708	.000	5.516
AgeGroups55=5	2.104	.000	8.200
AgeGroups55=6	0.971	.000	2.639
AgeGroups55=7	0.965	.000	2.625
AgeGroups55=8	0.302	.261	1.353
AgeGroups55=9	0[a]		
Jaw_=0	-0.106	.306	0.900
Jaw_=1	0[a]		

Probability distribution:Binomial
Link function:Logit

[a] This coefficient is set to zero because it is redundant.

Fixed Coefficients

Target:V
Reference Category:0

Model Term	Coefficient	Sig.
Intercept	-1.017	.000
Sex_=0	-0.273	.019
Sex_=1	0[a]	
AgeGroups55=1	0.718	.008
AgeGroups55=2	1.714	.000
AgeGroups55=3	1.766	.000
AgeGroups55=4	1.708	.000
AgeGroups55=5	2.104	.000
AgeGroups55=6	0.971	.000
AgeGroups55=7	0.965	.000
AgeGroups55=8	0.302	.261
AgeGroups55=9	0[a]	
Jaw_=0	-0.106	.306
Jaw_=1	0[a]	

Probability distribution:Binomial
Link function:Logit

[a] This coefficient is set to zero because it
redundant.

Fixed Coefficients

Target:V
Reference Category:0

Model Term	Coefficient	Std.Error	t	Sig.	Exp(Coefficient)
Intercept	-1.017	0.182	-5.603	.000	0.362
Sex_=0	-0.273	0.117	-2.342	.019	0.761
Sex_=1	0[a]				
AgeGroups55=1	0.718	0.273	2.635	.008	2.051
AgeGroups55=2	1.714	0.234	7.310	.000	5.549
AgeGroups55=3	1.766	0.214	8.272	.000	5.849
AgeGroups55=4	1.708	0.202	8.437	.000	5.516
AgeGroups55=5	2.104	0.220	9.585	.000	8.200
AgeGroups55=6	0.971	0.242	4.006	.000	2.639
AgeGroups55=7	0.965	0.267	3.608	.000	2.625
AgeGroups55=8	0.302	0.269	1.125	.261	1.353
AgeGroups55=9	0[a]				
Jaw_=0	-0.106	0.103	-1.023	.306	0.900
Jaw_=1	0[a]				

Probability distribution:Binomial
Link function:Logit

[a] This coefficient is set to zero because it is redundant.

Fixed Coefficients

Target:V
Reference Category:0

Model Term	95% Confidence Interval for Exp (Coefficient)	
	Lower	Upper
Intercept	0.253	0.516
Sex_=0	0.606	0.957
Sex_=1		
AgeGroups55=1	1.202	3.499
AgeGroups55=2	3.504	8.787
AgeGroups55=3	3.848	8.890
AgeGroups55=4	3.709	8.204
AgeGroups55=5	5.332	12.612
AgeGroups55=6	1.641	4.245
AgeGroups55=7	1.554	4.434
AgeGroups55=8	0.799	2.291
AgeGroups55=9		
Jaw_=0	0.735	1.102
Jaw_=1		

Probability distribution:Binomial
Link function:Logit

[a] This coefficient is set to zero because it is redundant.

Fixed Coefficients

Target:V
Reference Category:0

Model Term	Coefficient ▼	Std.Error	t	Sig.	95% Confidence Interval	
					Lower	Upper
Intercept	-1.017	0.182	-5.603	.000	-1.373	-0.661
Sex_=0	-0.273	0.117	-2.342	.019	-0.501	-0.044
Sex_=1	0[a]					
AgeGroups55=1	0.718	0.273	2.635	.008	0.184	1.253
AgeGroups55=2	1.714	0.234	7.310	.000	1.254	2.173
AgeGroups55=3	1.766	0.214	8.272	.000	1.348	2.185
AgeGroups55=4	1.708	0.202	8.437	.000	1.311	2.105
AgeGroups55=5	2.104	0.220	9.585	.000	1.674	2.535
AgeGroups55=6	0.971	0.242	4.006	.000	0.495	1.446
AgeGroups55=7	0.965	0.267	3.608	.000	0.441	1.489
AgeGroups55=8	0.302	0.269	1.125	.261	-0.225	0.829
AgeGroups55=9	0[a]					
Jaw_=0	-0.106	0.103	-1.023	.306	-0.308	0.097
Jaw_=1	0[a]					

Probability distribution:Binomial
Link function:Logit

[a] This coefficient is set to zero because it is redundant.

Random Effect Covariances

Target: V

Covariance

Negative — Positive

	Intercept
**Intercept**	1.214

Covariance Structure:Variance components
Subject Specification:ID

Random Effect Covariances

Target: V

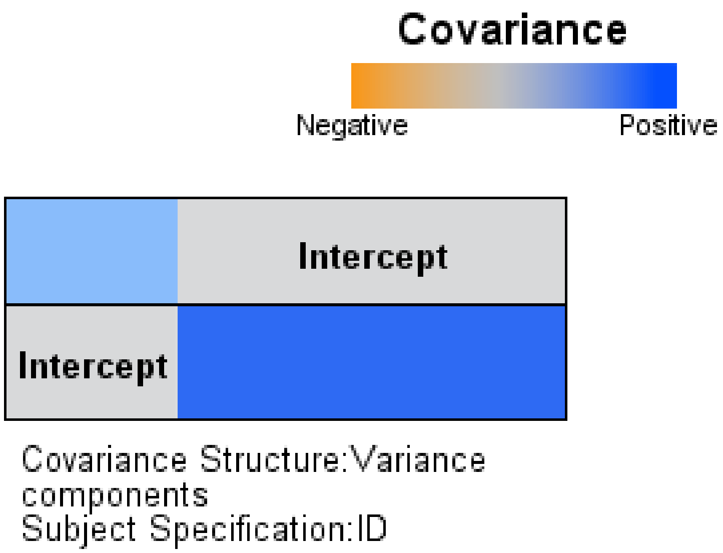

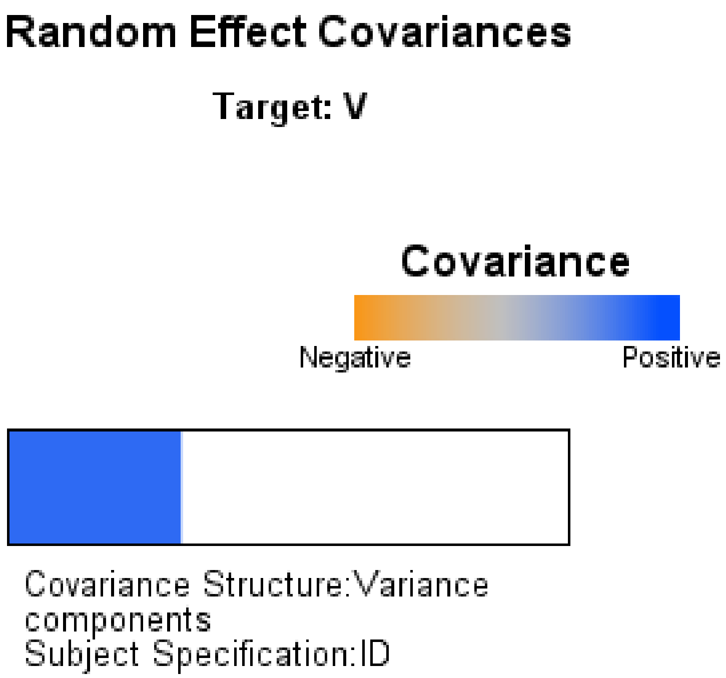
Random Effect Covariances
Target: V
Covariance
Negative
Positive
Covariance Structure:Variance components
Subject Specification:ID

Covariance Parameters

Target:V

Covariance Parameters	Residual Effect	0
	Random Effects	1
Design Matrix Columns	Fixed Effects	14
	Random Effects	1[a]
Common Subjects		1,979

Common subjects are based on the subject specifications for the residual and random effects and are used to chunk the data for better performance.

[a]This is the number of columns per common subject.

Residual Effect	Estimate	Std.Error	Z	Sig.	95% Confidence Interval	
					Lower	Upper
Variance	1.000	.	.	.	.	.

Covariance Structure:Scaled Identity
Subject Specification:(None)

Estimated Means: Significant Effects

Target: V

Estimated means charts for significant effects (p<.05) are displayed. Up to ten effects are displayed, beginning with the top three-way effects. Effects shown contain categorical predictors only.

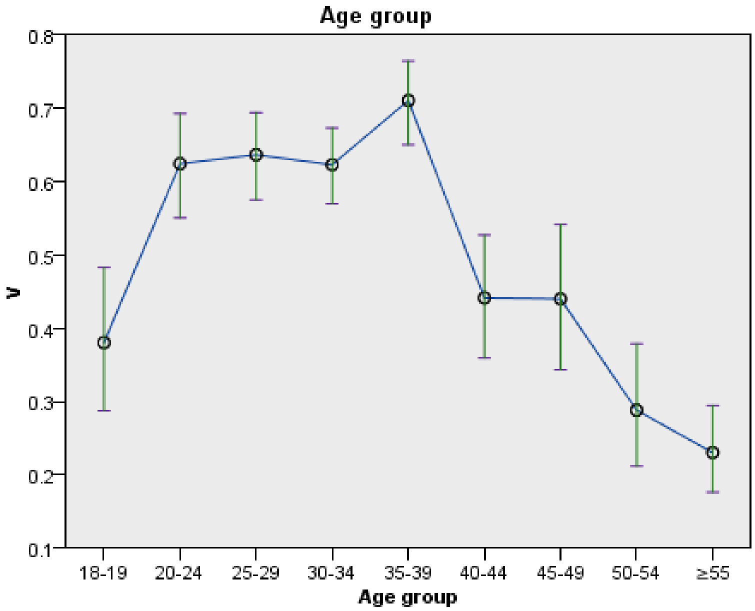

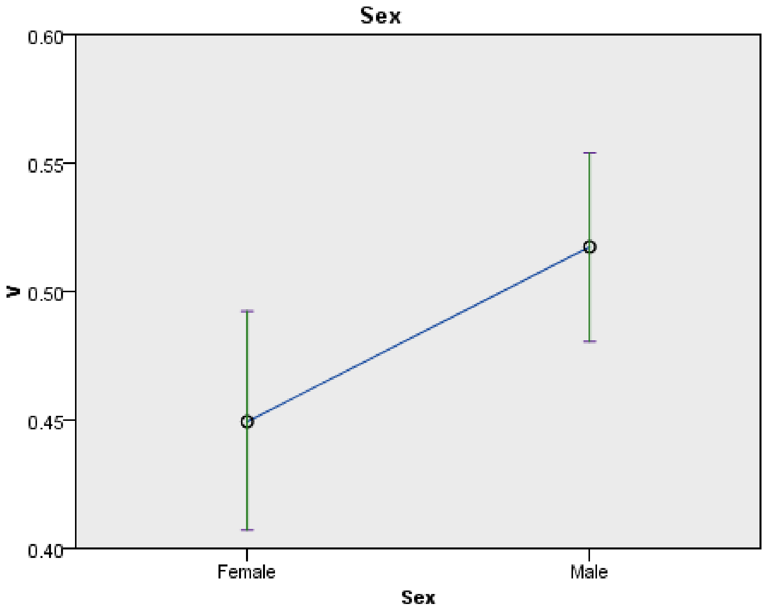

Generalized Linear Mixed Model

Generalized linear mixed models predict a continuous or categorical target based on one or more predictors. These models extend linear models to allow:

- Correlated data structures, including clustered and longitudinal designs.
- Non-normal targets such as binary, categorical, and count data.

- Data Structure
- Fields and Effects
  - Target
    - Target: VDi
    - Use number of trials as denominator: false
    - Customize reference category: true
    - Distribution: Binomial
    - Link function: Logit
  - Fixed Effects
  - Random Effects
- Build Options
- Training Summary

Model Summary

Target: H

**Target**		H
**Probability Distribution**		Binomial
**Link Function**		Logit
**Information Criterion**	**Akaike Corrected**	13,045.207
	**Bayesian**	13,050.989

Information criteria are based on the -2 log pseudo likelihood (13,043.206) and are used to compare models. Models with smaller information criterion values fit better. When comparing models using pseudo likelihood values, caution should be used because different data transformations may be used across the models.

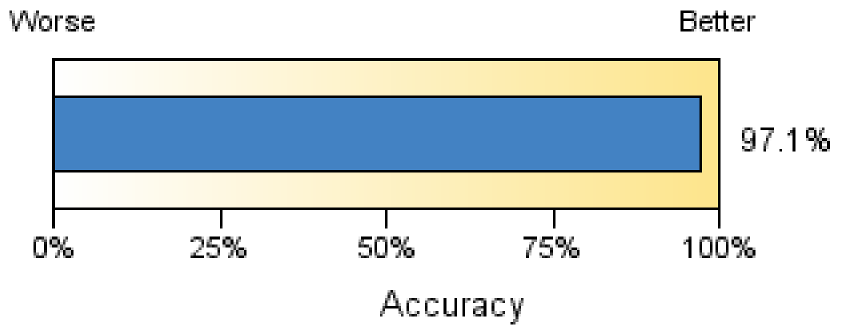

Data Structure

Target: H

	Subjects	Target
	Patient ID	H
Data For First Subject	1	0
Total Number of Levels	1979	

Classification

Target:H

Overall Percent Correct =97.1%

Observed	Predicted	
	1	0
1	0.0%	100.0%
0	0.0%	100.0%

Row Percent

100.00
80.00
60.00
40.00
20.00
0.00

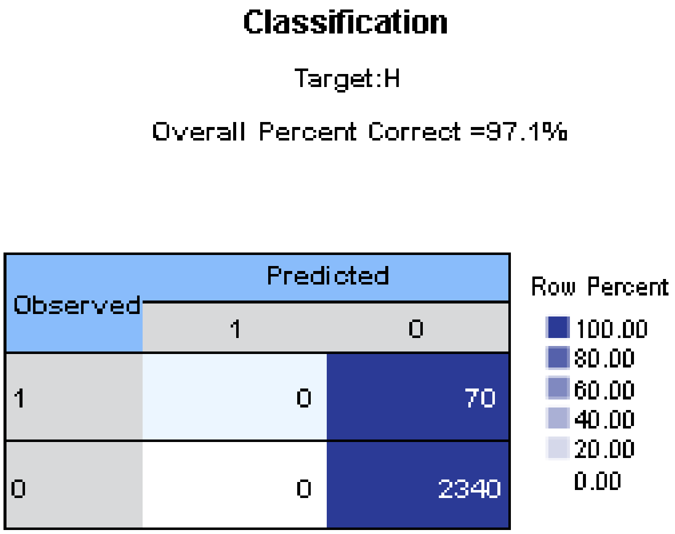
Classification
Target:H
Overall Percent Correct =97.1%
Predicted
Observed
1
0
1
0
70
0
0
2340
Row Percent
100.00
80.00
60.00
40.00
20.00
0.00

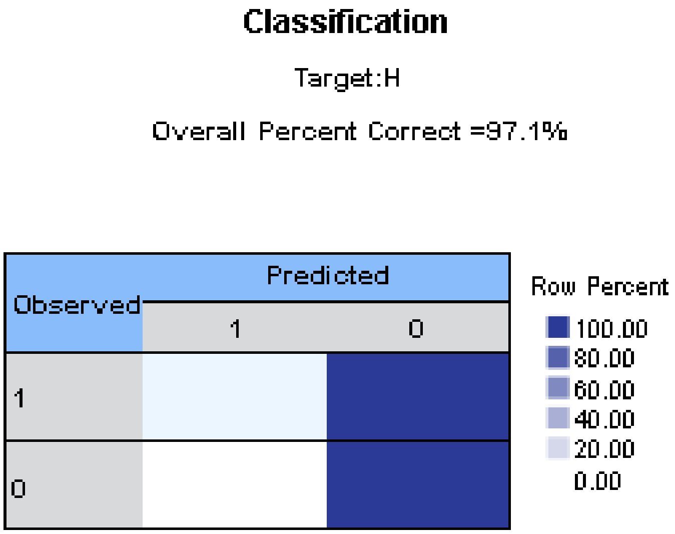
Classification
Target:H
Overall Percent Correct =97.1%
Predicted
Observed
1
0
1
0
Row Percent
100.00
80.00
60.00
40.00
20.00
0.00

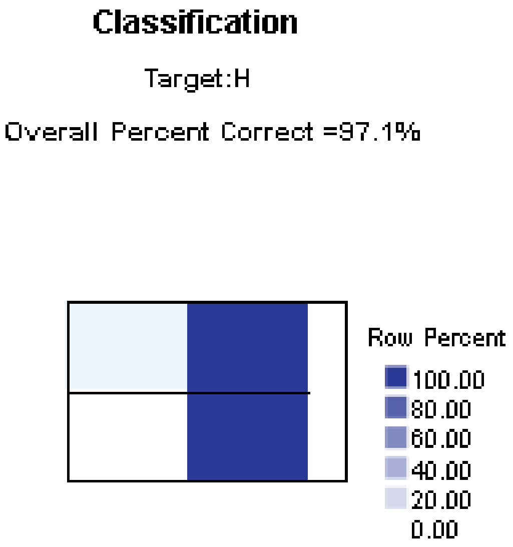

Classification
Target:H
Overall Percent Correct =97.1%
Row Percent
100.00
80.00
60.00
40.00
20.00
0.00

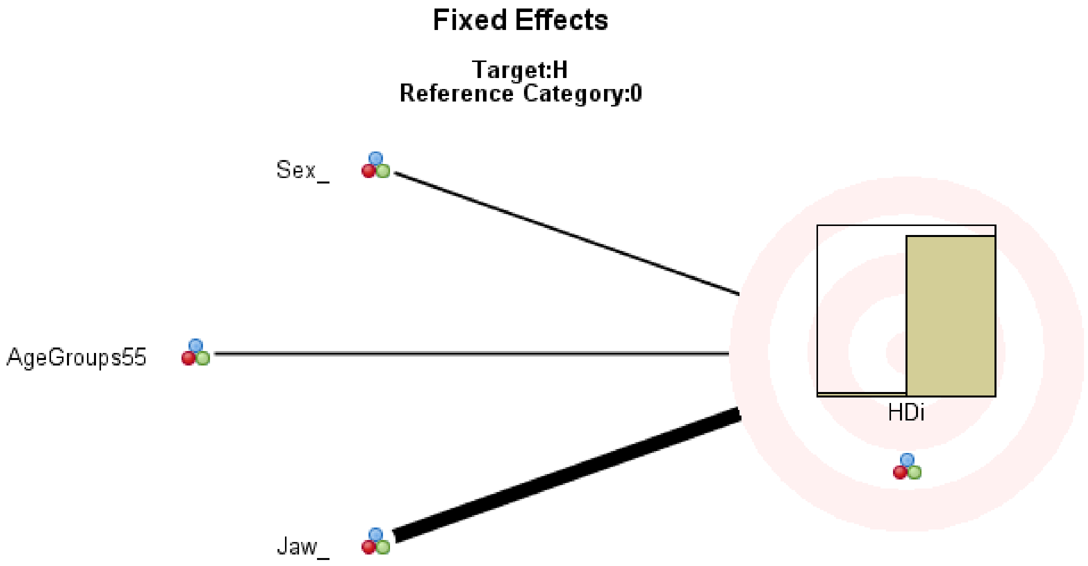

Fixed Effects
Target:H
Reference Category:0
Sex_
AgeGroups55
Jaw_
HDi

Fixed Effects

Target:H
Reference Category:0

Source	F	df1	df2	Sig.
Corrected Model	2.782	10	2,399	.002

Probability distribution:Binomial
Link function:Logit

Fixed Effects

Target:H
Reference Category:0

Source	F	df1	df2	Sig.
Corrected Model ▼	2.782	10	2,399	.002
Sex_	0.004	1	2,399	.952
AgeGroups55	1.267	8	2,399	.256
Jaw_	17.818	1	2,399	.000

Probability distribution:Binomial
Link function:Logit

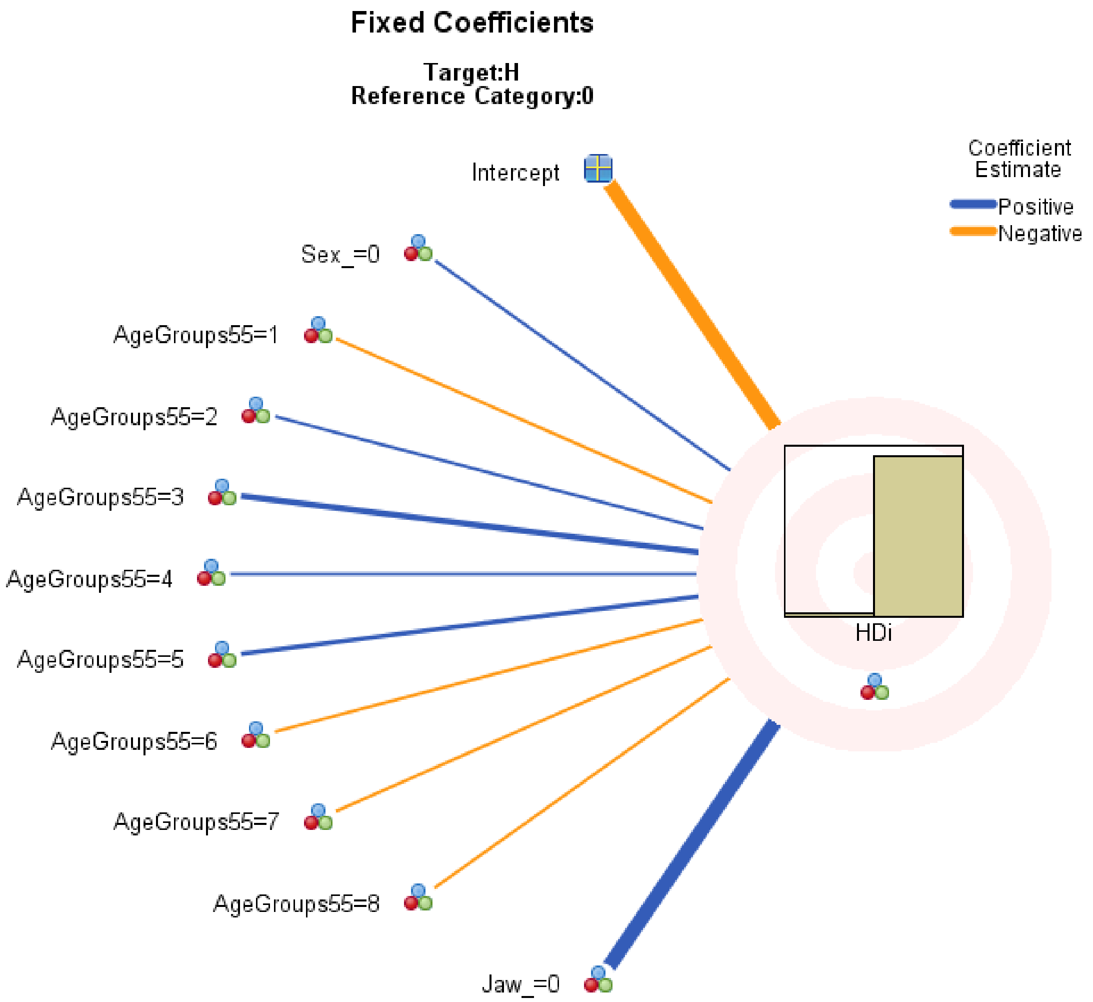

Fixed Coefficients
Target:H
Reference Category:0
Coefficient Estimate
Positive
Negative
Intercept
Sex_=0
AgeGroups55=1
AgeGroups55=2
AgeGroups55=3
AgeGroups55=4
AgeGroups55=5
AgeGroups55=6
AgeGroups55=7
AgeGroups55=8
Jaw_=0
HDi

Fixed Coefficients

Target:H
Reference Category:0

Model Term	Coefficient	Sig.	Exp(Coefficient)
Intercept	-3.765	.000	0.023
Sex_=0	0.013	.952	1.013
Sex_=1	0[a]		
AgeGroups55=1	-0.231	.704	0.794
AgeGroups55=2	0.503	.232	1.653
AgeGroups55=3	0.648	.083	1.911
AgeGroups55=4	0.196	.606	1.217
AgeGroups55=5	0.524	.162	1.688
AgeGroups55=6	-0.041	.935	0.960
AgeGroups55=7	-0.232	.651	0.793
AgeGroups55=8	-0.232	.687	0.793
AgeGroups55=9	0[a]		
Jaw_=0	0.869	.000	2.384
Jaw_=1	0[a]		

Probability distribution:Binomial
Link function:Logit

[a] This coefficient is set to zero because it is redundant.

Fixed Coefficients

Target:H
Reference Category:0

Model Term	Coefficient	Sig.
Intercept	-3.765	.000
Sex_=0	0.013	.952
Sex_=1	0[a]	
AgeGroups55=1	-0.231	.704
AgeGroups55=2	0.503	.232
AgeGroups55=3	0.648	.083
AgeGroups55=4	0.196	.606
AgeGroups55=5	0.524	.162
AgeGroups55=6	-0.041	.935
AgeGroups55=7	-0.232	.651
AgeGroups55=8	-0.232	.687
AgeGroups55=9	0[a]	
Jaw_=0	0.869	.000
Jaw_=1	0[a]	

Probability distribution:Binomial
Link function:Logit

[a]This coefficient is set to zero because it
redundant.

Fixed Coefficients

Target:H
Reference Category:0

Model Term	Coefficient	Std.Error	t	Sig.	Exp(Coefficient)
Intercept	-3.765	0.349	-10.795	.000	0.023
Sex_=0	0.013	0.221	0.061	.952	1.013
Sex_=1	0[a]				
AgeGroups55=1	-0.231	0.609	-0.380	.704	0.794
AgeGroups55=2	0.503	0.420	1.196	.232	1.653
AgeGroups55=3	0.648	0.374	1.733	.083	1.911
AgeGroups55=4	0.196	0.379	0.516	.606	1.217
AgeGroups55=5	0.524	0.374	1.399	.162	1.688
AgeGroups55=6	-0.041	0.502	-0.081	.935	0.960
AgeGroups55=7	-0.232	0.512	-0.452	.651	0.793
AgeGroups55=8	-0.232	0.576	-0.403	.687	0.793
AgeGroups55=9	0[a]				
Jaw_=0	0.869	0.206	4.221	.000	2.384
Jaw_=1	0[a]				

Probability distribution:Binomial
Link function:Logit

[a] This coefficient is set to zero because it is redundant.

Fixed Coefficients

Target:H
Reference Category:0

Model Term	95% Confidence Interval for Exp (Coefficient)	
	Lower	Upper
Intercept	0.012	0.046
Sex_=0	0.657	1.562
Sex_=1		
AgeGroups55=1	0.241	2.619
AgeGroups55=2	0.725	3.769
AgeGroups55=3	0.918	3.978
AgeGroups55=4	0.578	2.560
AgeGroups55=5	0.810	3.517
AgeGroups55=6	0.358	2.570
AgeGroups55=7	0.291	2.165
AgeGroups55=8	0.256	2.455
AgeGroups55=9		
Jaw_=0	1.592	3.570
Jaw_=1		

Probability distribution:Binomial
Link function:Logit

[a] This coefficient is set to zero because it is redundant.

Fixed Coefficients

Target:H
Reference Category:0

Model Term	Coefficient	Std.Error	t	Sig.	95% Confidence Interval	
					Lower	Upper
Intercept	-3.765	0.349	-10.795	.000	-4.449	-3.081
Sex_=0	0.013	0.221	0.061	.952	-0.419	0.446
Sex_=1	0[a]					
AgeGroups55=1	-0.231	0.609	-0.380	.704	-1.425	0.963
AgeGroups55=2	0.503	0.420	1.196	.232	-0.321	1.327
AgeGroups55=3	0.648	0.374	1.733	.083	-0.085	1.381
AgeGroups55=4	0.196	0.379	0.516	.606	-0.548	0.940
AgeGroups55=5	0.524	0.374	1.399	.162	-0.210	1.258
AgeGroups55=6	-0.041	0.502	-0.081	.935	-1.026	0.944
AgeGroups55=7	-0.232	0.512	-0.452	.651	-1.235	0.772
AgeGroups55=8	-0.232	0.576	-0.403	.687	-1.363	0.898
AgeGroups55=9	0[a]					
Jaw_=0	0.869	0.206	4.221	.000	0.465	1.273
Jaw_=1	0[a]					

Probability distribution:Binomial
Link function:Logit

[a]This coefficient is set to zero because it is redundant.

Random Effect Covariances

Target: H

Covariance

Negative Positive

	Intercept
**Intercept**	0.000

Covariance Structure:Variance components
Subject Specification:ID

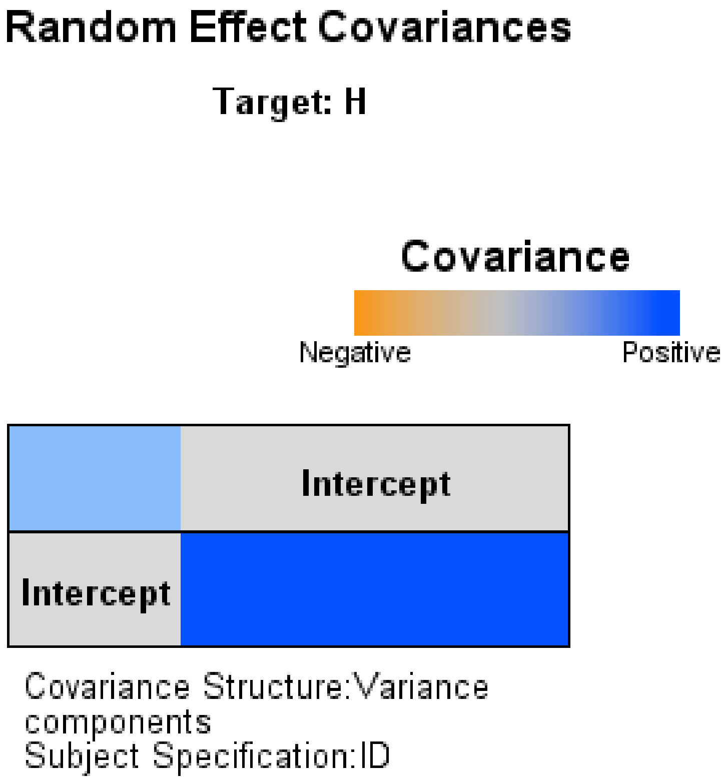
Random Effect Covariances
Target: H
Covariance
Negative
Positive
Intercept
Intercept
Covariance Structure:Variance components
Subject Specification:ID

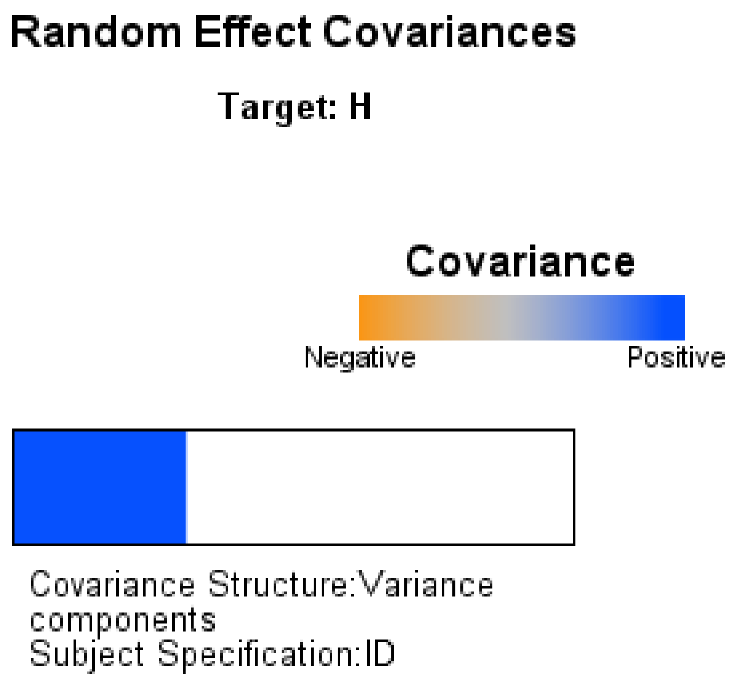
Random Effect Covariances
Target: H
Covariance
Negative
Positive
Covariance Structure:Variance components
Subject Specification:ID

Covariance Parameters

Target:H

Covariance Parameters	Residual Effect	0
	Random Effects	1
Design Matrix Columns	Fixed Effects	14
	Random Effects	1[a]
Common Subjects		1,979

Common subjects are based on the subject specifications for the residual and random effects and are used to chunk the data for better performance.

[a] This is the number of columns per common subject.

Residual Effect	Estimate	Std.Error	Z	Sig.	95% Confidence Interval	
					Lower	Upper
Variance	1.000	.	.	.	.	.

Covariance Structure:Scaled Identity
Subject Specification:(None)

Estimated Means: Significant Effects

Target: H

Estimated means charts for significant effects (p<.05) are displayed. Up to ten effects are displayed, beginning with the top three-way effects. Effects shown contain categorical predictors only.

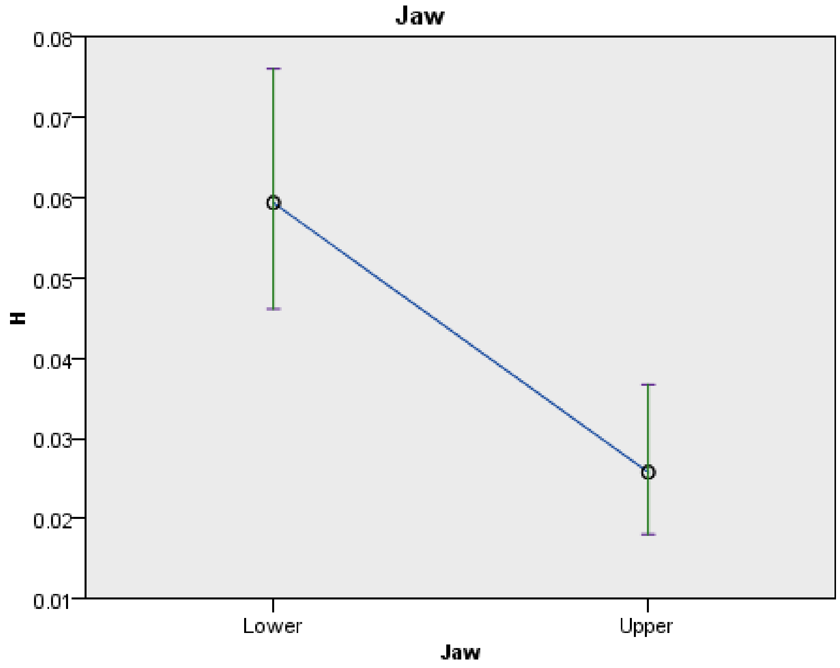

Generalized Linear Mixed Model

Generalized linear mixed models predict a continuous or categorical target based on one or more predictors. These models extend linear models to allow:

- Correlated data structures, including clustered and longitudinal designs.
- Non-normal targets such as binary, categorical, and count data.

- Data Structure
- Fields and Effects
  - Target
    - Target: HDi
    - Use number of trials as denominator: false
    - Customize reference category: true
    - Distribution: Binomial
    - Link function: Logit
  - Fixed Effects
  - Random Effects
- Build Options
- Training Summary

Model Summary

Target: T

**Target**		T
**Probability Distribution**		Binomial
**Link Function**		Logit
**Information Criterion**	**Akaike Corrected**	13,051.927
	**Bayesian**	13,057.708

Information criteria are based on the -2 log pseudo likelihood (13,049.925) and are used to compare models. Models with smaller information criterion values fit better. When comparing models using pseudo likelihood values, caution should be used because different data transformations may be used across the models.

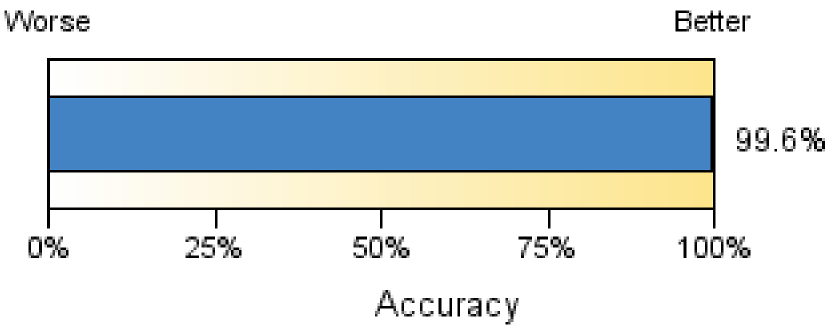

Data Structure

Target: T

	Subjects	Target
	Patient ID	T
Data For First Subject	1	0
Total Number of Levels	1979	

Classification

Target:T

Overall Percent Correct =99.6%

Observed	Predicted	
	1	0
1	0.0%	100.0%
0	0.0%	100.0%

Row Percent

100.00
80.00
60.00
40.00
20.00
0.00

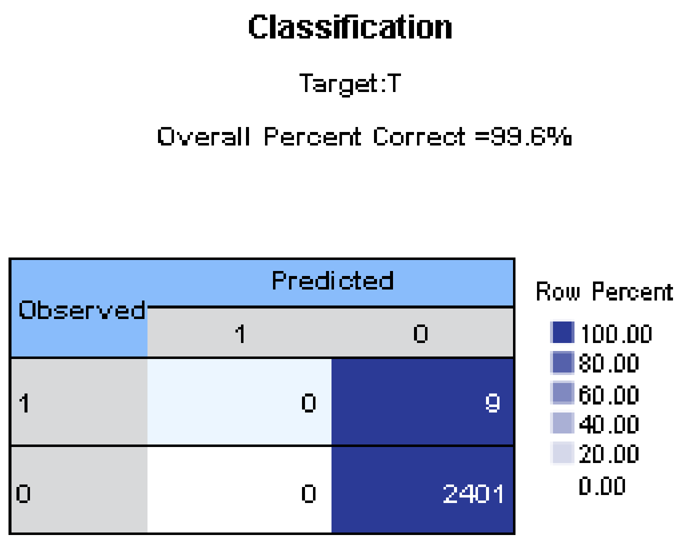
Classification
Target:T
Overall Percent Correct =99.6%
Predicted
Observed
1
0
1
0
9
0
0
2401
Row Percent
100.00
80.00
60.00
40.00
20.00
0.00

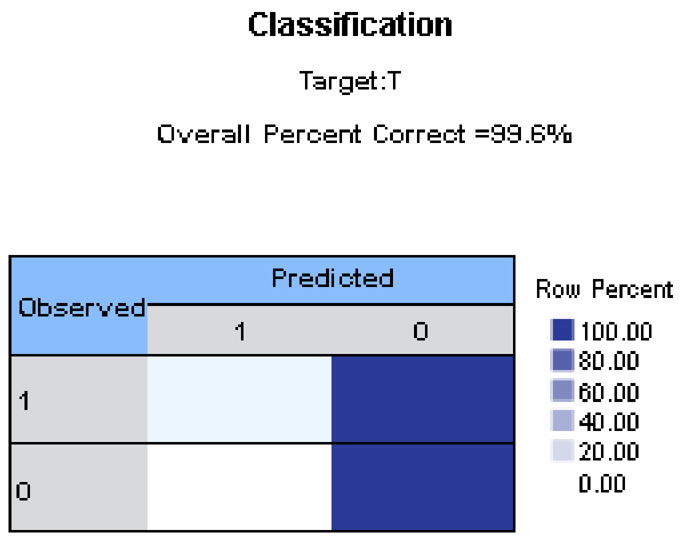
Classification
Target:T
Overall Percent Correct =99.6%
Predicted
Observed
1
0
1
0
Row Percent
100.00
80.00
60.00
40.00
20.00
0.00

Classification

Target:T

Overall Percent Correct =99.6%

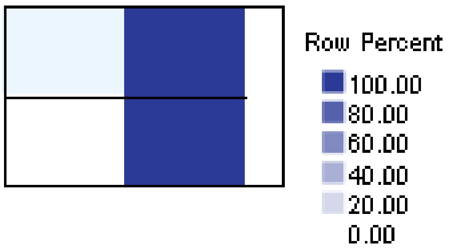

Fixed Effects

Target:T
Reference Category:0

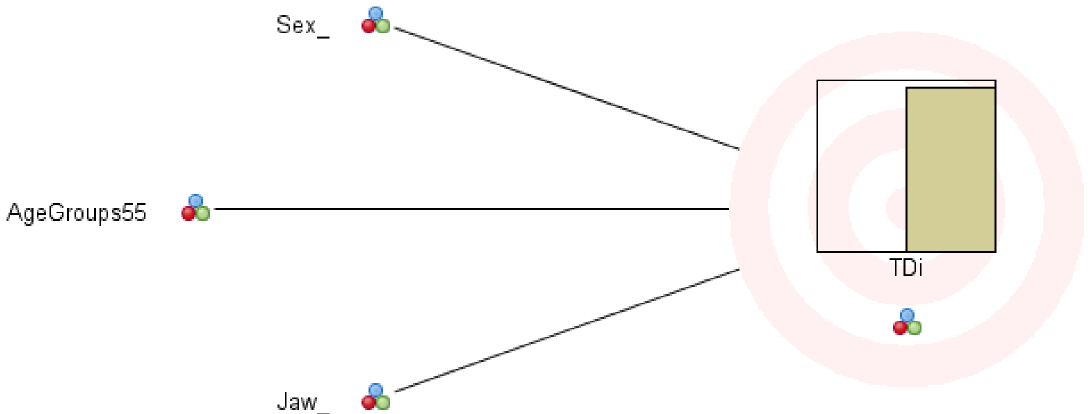

Fixed Effects

Target:T
Reference Category:0

Source	F	df1	df2	Sig.
Corrected Model	0.787	10	2,399	.642

Probability distribution:Binomial
Link function:Logit

Fixed Effects

Target:T
Reference Category:0

Source	F	df1	df2	Sig.
Corrected Model ▼	0.787	10	2,399	.642
Sex_	0.472	1	2,399	.492
AgeGroups55	0.873	8	2,399	.538
Jaw_	0.313	1	2,399	.576

Probability distribution:Binomial
Link function:Logit

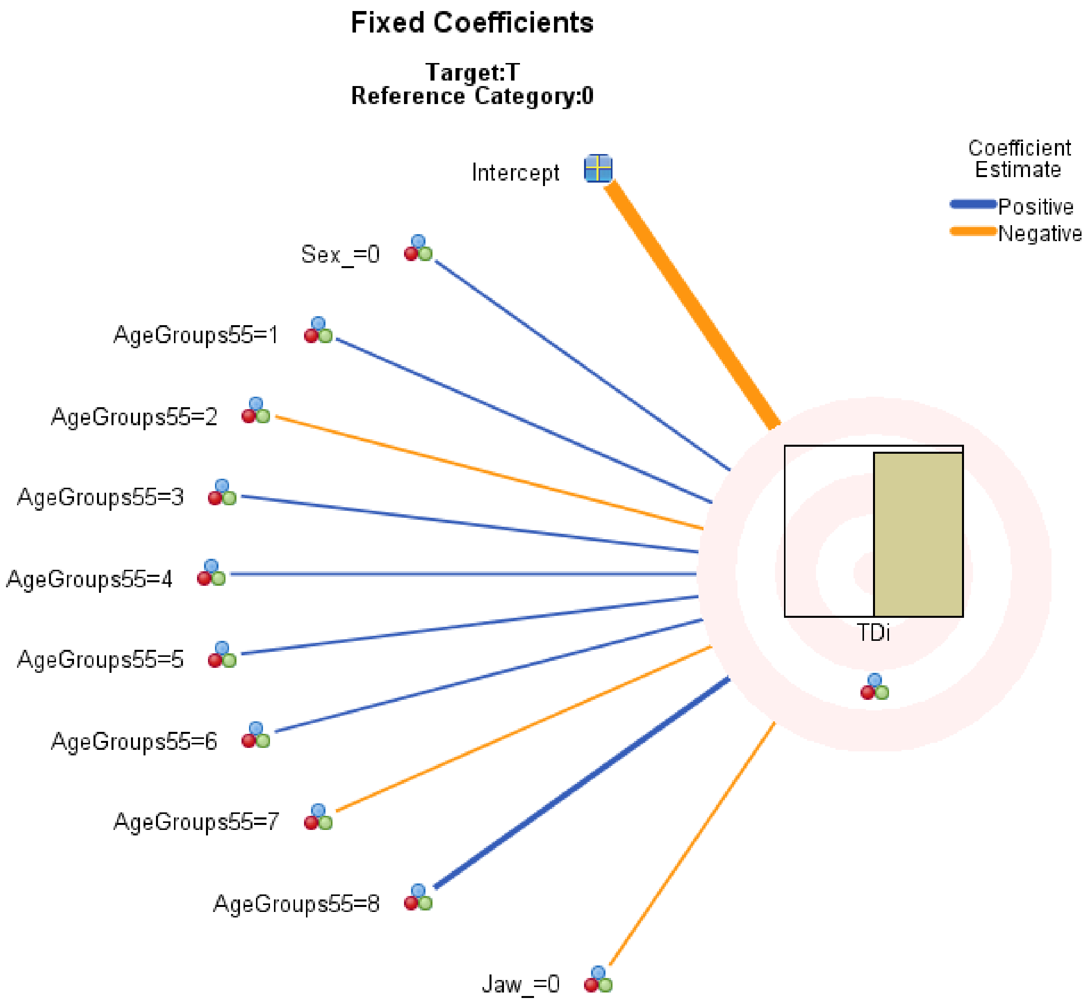
Fixed Coefficients
Target:T
Reference Category:0
Coefficient Estimate
Positive
Negative
Intercept
Sex_=0
AgeGroups55=1
AgeGroups55=2
AgeGroups55=3
AgeGroups55=4
AgeGroups55=5
AgeGroups55=6
AgeGroups55=7
AgeGroups55=8
Jaw_=0
TDi

Fixed Coefficients

Target:T
Reference Category:0

Model Term	Coefficient	Sig.	Exp(Coefficient)
Intercept	-3.522	.000	0.030
Sex_=0	0.176	.492	1.193
Sex_=1	0[a]		
AgeGroups55=1	0.003	.997	1.003
AgeGroups55=2	-0.109	.836	0.897
AgeGroups55=3	0.006	.990	1.006
AgeGroups55=4	0.005	.991	1.005
AgeGroups55=5	0.025	.957	1.025
AgeGroups55=6	0.010	.985	1.010
AgeGroups55=7	-0.002	.997	0.998
AgeGroups55=8	0.913	.056	2.492
AgeGroups55=9	0[a]		
Jaw_=0	-0.131	.576	0.877
Jaw_=1	0[a]		

Probability distribution:Binomial
Link function:Logit

[a] This coefficient is set to zero because it is redundant.

Fixed Coefficients

Target:T
Reference Category:0

Model Term	Coefficient	Sig.
Intercept	-3.522	.000
Sex_=0	0.176	.492
Sex_=1	0[a]	
AgeGroups55=1	0.003	.997
AgeGroups55=2	-0.109	.836
AgeGroups55=3	0.006	.990
AgeGroups55=4	0.005	.991
AgeGroups55=5	0.025	.957
AgeGroups55=6	0.010	.985
AgeGroups55=7	-0.002	.997
AgeGroups55=8	0.913	.056
AgeGroups55=9	0[a]	
Jaw_=0	-0.131	.576
Jaw_=1	0[a]	

Probability distribution:Binomial
Link function:Logit

[a] This coefficient is set to zero because it redundant.

Fixed Coefficients

Target:T
Reference Category:0

Model Term	Coefficient	Std.Error	t	Sig.	Exp(Coefficient)
Intercept	-3.522	0.380	-9.278	.000	0.030
Sex_=0	0.176	0.257	0.687	.492	1.193
Sex_=1	0[a]				
AgeGroups55=1	0.003	0.639	0.004	.997	1.003
AgeGroups55=2	-0.109	0.526	-0.207	.836	0.897
AgeGroups55=3	0.006	0.475	0.013	.990	1.006
AgeGroups55=4	0.005	0.445	0.012	.991	1.005
AgeGroups55=5	0.025	0.463	0.054	.957	1.025
AgeGroups55=6	0.010	0.563	0.018	.985	1.010
AgeGroups55=7	-0.002	0.544	-0.004	.997	0.998
AgeGroups55=8	0.913	0.478	1.912	.056	2.492
AgeGroups55=9	0[a]				
Jaw_=0	-0.131	0.235	-0.559	.576	0.877
Jaw_=1	0[a]				

Probability distribution:Binomial
Link function:Logit

[a] This coefficient is set to zero because it is redundant.

Fixed Coefficients

Target:T
Reference Category:0

Model Term	95% Confidence Interval for Exp (Coefficient)	
	Lower	Upper
Intercept	0.014	0.062
Sex_=0	0.721	1.975
Sex_=1		
AgeGroups55=1	0.286	3.513
AgeGroups55=2	0.320	2.516
AgeGroups55=3	0.397	2.551
AgeGroups55=4	0.420	2.404
AgeGroups55=5	0.413	2.544
AgeGroups55=6	0.335	3.048
AgeGroups55=7	0.344	2.899
AgeGroups55=8	0.977	6.356
AgeGroups55=9		
Jaw_=0	0.553	1.390
Jaw_=1		

Probability distribution:Binomial
Link function:Logit

[a] This coefficient is set to zero because it is redundant.

Fixed Coefficients

Target:T
Reference Category:0

Model Term	Coefficient	Std.Error	t	Sig.	95% Confidence Interval	
					Lower	Upper
Intercept	-3.522	0.380	-9.278	.000	-4.266	-2.777
Sex_=0	0.176	0.257	0.687	.492	-0.327	0.680
Sex_=1	0[a]					
AgeGroups55=1	0.003	0.639	0.004	.997	-1.251	1.256
AgeGroups55=2	-0.109	0.526	-0.207	.836	-1.141	0.923
AgeGroups55=3	0.006	0.475	0.013	.990	-0.924	0.937
AgeGroups55=4	0.005	0.445	0.012	.991	-0.867	0.877
AgeGroups55=5	0.025	0.463	0.054	.957	-0.884	0.934
AgeGroups55=6	0.010	0.563	0.018	.985	-1.094	1.115
AgeGroups55=7	-0.002	0.544	-0.004	.997	-1.068	1.064
AgeGroups55=8	0.913	0.478	1.912	.056	-0.024	1.849
AgeGroups55=9	0[a]					
Jaw_=0	-0.131	0.235	-0.559	.576	-0.592	0.329
Jaw_=1	0[a]					

Probability distribution:Binomial
Link function:Logit

[a] This coefficient is set to zero because it is redundant.

Random Effect Covariances

Target: T

Covariance

Negative Positive

	Intercept
**Intercept**	0.000

Covariance Structure:Variance components
Subject Specification:ID

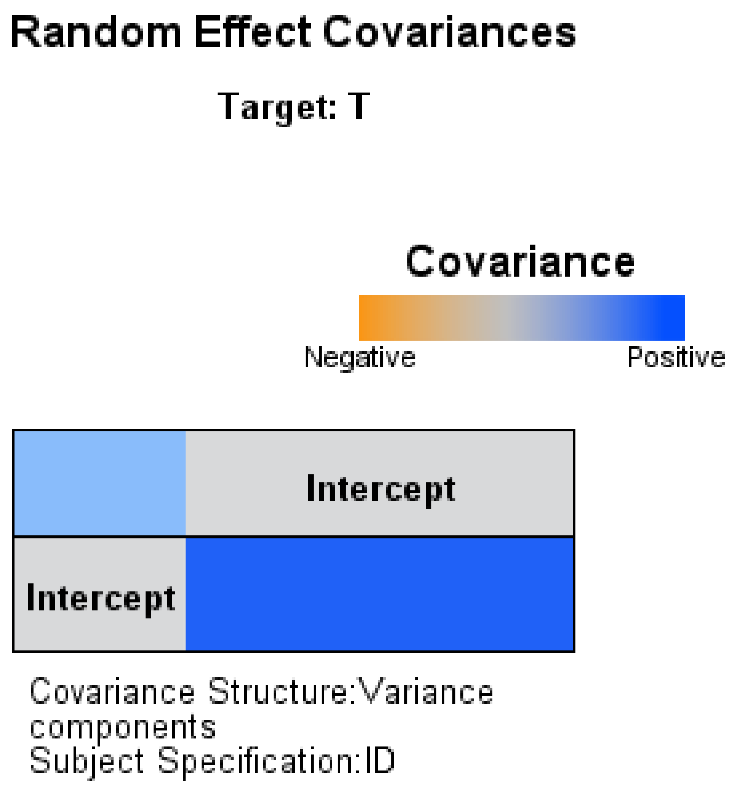
Random Effect Covariances
Target: T
Covariance
Negative
Positive
Intercept
Intercept
Covariance Structure:Variance components
Subject Specification:ID

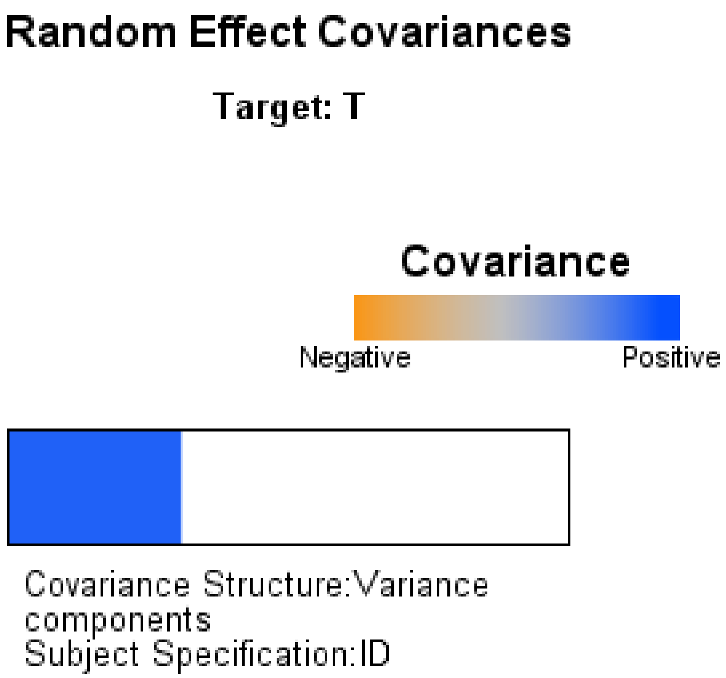
Random Effect Covariances
Target: T
Covariance
Negative
Positive
Covariance Structure:Variance components
Subject Specification:ID

Covariance Parameters

Target:T

**Covariance Parameters**	**Residual Effect**	0
	**Random Effects**	1
**Design Matrix Columns**	**Fixed Effects**	14
	**Random Effects**	1[a]
**Common Subjects**		1,979

Common subjects are based on the subject specifications for the residual and random effects and are used to chunk the data for better performance.

[a] This is the number of columns per common subject.

Residual Effect	Estimate	Std.Error	Z	Sig.	95% Confidence Interval	
					Lower	Upper
**Variance**	1.000	.	.	.	.	.

Covariance Structure:Scaled Identity
Subject Specification:(None)

Generalized Linear Mixed Model

Generalized linear mixed models predict a continuous or categorical target based on one or more predictors. These models extend linear models to allow:

- Correlated data structures, including clustered and longitudinal designs.
- Non-normal targets such as binary, categorical, and count data.

- Data Structure
- Fields and Effects
  - Target
    - Target: TDi
    - Use number of trials as denominator: false
    - Customize reference category: true
    - Distribution: Binomial
    - Link function: Logit
  - Fixed Effects
  - Random Effects
- Build Options
- Training Summary
